# Exploring a general multi-pronged activation strategy for natural product discovery in Actinomycetes

**DOI:** 10.1038/s42003-023-05648-7

**Published:** 2024-01-06

**Authors:** Dillon W. P. Tay, Lee Ling Tan, Elena Heng, Nadiah Zulkarnain, Kuan Chieh Ching, Mario Wibowo, Elaine Jinfeng Chin, Zann Yi Qi Tan, Chung Yan Leong, Veronica Wee Pin Ng, Lay Kien Yang, Deborah C. S. Seow, Yi Wee Lim, Winston Koh, Lokanand Koduru, Yoganathan Kanagasundaram, Siew Bee Ng, Yee Hwee Lim, Fong Tian Wong

**Affiliations:** 1 Institute of Sustainability for Chemicals, Energy and Environment (ISCE2), Agency for Science, Technology and Research (A*STAR), 8 Biomedical Grove, #07-01 Neuros Building, Singapore, 138665 Republic of Singapore; 2 Molecular Engineering Lab, Institute of Molecular and Cell Biology (IMCB), Agency for Science, Technology and Research (A*STAR), 61 Biopolis Drive, #07-06, Proteos, Singapore, 138673 Republic of Singapore; 3 Singapore Institute of Food and Biotechnology Innovation (SIFBI), Agency for Science, Technology and Research (A*STAR), 31 Biopolis Way, #01-02, Nanos, Singapore, 138669 Republic of Singapore; 4 Bioinformatics Institute (BII), Agency of Science, Technology and Research (A*STAR), 30 Biopolis Street, #07-01, Matrix, Singapore, 138671 Republic of Singapore; 5 Synthetic Biology Translational Research Program, Yong Loo Lin School of Medicine, National University of Singapore, 10 Medical Drive, Singapore, 117597 Republic of Singapore

**Keywords:** Applied microbiology, Genetic engineering, Metabolic engineering

## Abstract

Natural products possess significant therapeutic potential but remain underutilized despite advances in genomics and bioinformatics. While there are approaches to activate and upregulate natural product biosynthesis in both native and heterologous microbial strains, a comprehensive strategy to elicit production of natural products as well as a generalizable and efficient method to interrogate diverse native strains collection, remains lacking. Here, we explore a flexible and robust integrase-mediated multi-pronged activation approach to reliably perturb and globally trigger antibiotics production in actinobacteria. Across 54 actinobacterial strains, our approach yielded 124 distinct activator-strain combinations which consistently outperform wild type. Our approach expands accessible metabolite space by nearly two-fold and increases selected metabolite yields by up to >200-fold, enabling discovery of Gram-negative bioactivity in tetramic acid analogs. We envision these findings as a gateway towards a more streamlined, accelerated, and scalable strategy to unlock the full potential of Nature’s chemical repertoire.

## Abstract (web summary)

A multi-pronged activation approach applied to 54 actinobacterial strains doubles accessible metabolite space and enables discovery of gram-negative bioactivity, offering insights to harness nature’s chemical potential. The approach enhances or triggers natural product production across all 54 native actinobacterial strains, doubling total metabolite production and enabling the discovery of a new tetramic acid analog with gram-negative bioactivity. Insights from this large-scale study could lead to a more streamlined approach to unlock the full potential of nature’s chemical resources.

## Introduction

Natural products (NPs) are a family of highly diverse molecules that have been a valuable source of bioactive compounds with demonstrated applications in food^1^, agriculture^2^, and most prominently in therapeutics^3^, where an estimated 50% of non-biologics are NP, NP-derived or NP mimics^4^. However, the discovery of bioactive NPs via traditional bioactivity-guided screening methods has been a low-yielding investment since the end of the golden age of exploration in the early 1960s^3^. With the advent of omics science and recent computational developments, NP research has experienced a revival, bringing about a new era of optimism and discovery^5^.

The recent rapid rise in genomic data collection and automated bioinformatics^6,7^ has led to an exponential increase in the number and diversity of annotated biosynthetic gene clusters (BGCs) demonstrating Nature’s chemical potential^7,8^. However, microbes have evolved to react to environmental cues and consequently, most encoded BGCs are expected to be cryptic^9^ under lab conditions. To unlock the chemical repertoire of Nature’s biodiversity and tap into this vast hidden resource represented by silent and poorly expressing BGCs, a variety of genetic and non-genetic strategies have been developed to activate and upregulate NP biosyntheses in native and heterologous microbial strains^10–13^. While advances in heterologous-based technologies and synthetic biology have enabled the examination of a large number of BGCs^14,15^, successful production rates range from 24% to 69%. Similarly, precise targeting and overexpression of biosynthetic pathways in native strains are not always successful. This led us to hypothesize the presence of global inhibitions within strains beyond specific biosynthetic pathways. From a metabolic engineering perspective, the reduced productivity exhibited by these native strains under laboratory conditions could possibly be attributed to constraints on precursors for secondary metabolites^16,17^, transcriptionally silent biosynthetic gene clusters^18,19^, or being in an unsuitable phase/media for secondary metabolite production^20–23^. Therefore, permanently enhancing these native strains for secondary metabolite production while capitalizing on their naturally evolved regulatory and metabolic processes for biosynthesis could offer a more efficient approach to access extensive untapped chemical diversity.

**Fig. 1:**
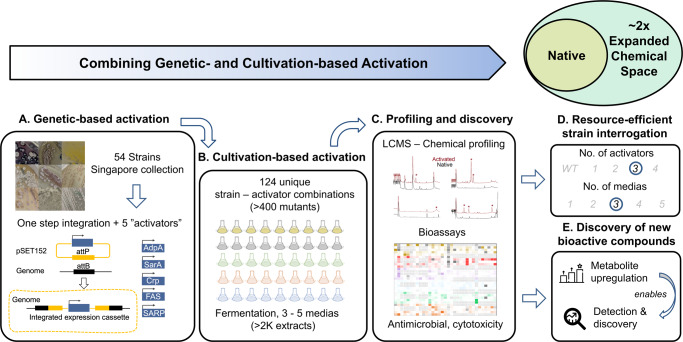
Schematics of a multi-pronged general activation strategy for native microbial strains toward natural product discovery. **A** Microbial strains are genetically edited via one-step phiC31 integrase protocol for heterologous expression of genes for activation and/or upregulation of secondary metabolites. **B** Genetic-based activation is complemented with cultivation-based (one strain many compounds, OSMAC) activation where a range of fermentation media (with varied nutrient compositions) is employed to probe the full biosynthetic potential of the genetically edited mutants. **C** Extracts are profiled both chemically and with bioassays to characterize and discover new bioactive metabolite space. Bioactivity assays include antibacterial assays (*Acinetobacter baumannii, Klebsiella aerogenes, Pseudomonas aeruginosa* and *Staphylococcus aureus Rosenbach*), antifungal assays (*Aspergillus fumigatus*) and mammalian cytotoxicity assays (A549 human lung carcinoma cells). **D** In depth analysis of LCMS data allowed us to interrogate the effects of media and activators where we found the combination of 3 regulators and 3 media were sufficient to produce maximal metabolites production. **E** Bioactivity profile comparison between mutants and native strains revealed that the expanded chemical space was accompanied by increased or novel bioactivities, which were used for new NP discovery.

Here, we demonstrate a highly robust, flexible, and efficient approach featuring one-step integrase-mediated genetic activation with “one strain many compounds” OSMAC requirements^10,22,23^ to globally perturb and upregulate silent and/or low-yielding secondary metabolites (Fig. 1) across a range of 54 diverse actinobacterial strains^24^. Our approach exploits the well-established *Streptomyces* phage-derived phiC31 integrase to enable consistent gene editing across actinobacterial strains without dependence on genomic information^17^. This allows for integration and subsequent constitutive expression of a library of “activators” for secondary metabolism and antibiotics production under a strong constitutive promoter. This scalable approach has enabled us to examine a set of unique actinobacterial strains, comprising mainly *Streptomyces* and *Micromonospora* (Table S1) isolated from both soil and marine environments in Singapore (Natural organism library, NOL)^25^. Through molecular networking analyses, we observe an approximate two-fold expansion in metabolite space with activation, and >200-fold upregulation of selected secondary metabolite production in actinomycetes. Finally, by deploying differential biological profiling analysis, our multi-pronged general activation strategy for NP discovery has enabled us to uncover and characterize the first example of growth inhibitory activity against *Acinetobacter baumannii* in tetramic acid-derived molecules.

## Results and discussion

### A robust phiC31 integrase-mediated strategy for native strain activation

To examine positive upregulation within the native strains, we elected to employ a multi-pronged strategy to investigate the effects of (1) modulating the native strain environment, (2) regulation targeting biosynthetic gene clusters, and (3) increasing substrate flux. To obtain this objective, we introduced “activators” to upregulate and tune the native strain environment including conserved morphological regulators: cyclic AMP receptor protein (Crp)^16^ and A-factor dependent protein A (AdpA) that affect sporulation and morphology^21^ while regulating balances between primary and secondary metabolites. The potential of a sporulation and antibiotics-related gene A protein (SarA)^20^ for secondary metabolite activation was also investigated for similar functions to Crp and AdpA. Additionally, we investigated effect of the highly efficient *Streptomyces* antibiotic regulatory protein (SARP, RedD) as pathway-specific activators of secondary metabolite biosynthesis^18,19^. Finally, we also included the fatty acyl CoA synthase (FAS) gene which has been recently demonstrated to mobilize triacylglycerols (TAG) flux for increased antibiotics production^17^. To overexpress these within native strains, genetic editing is an attractive option as it confers stability. Moreover, within the actinobacteria, we have previously observed moderate success with genetic editing^11–13^.

**Fig. 2:**
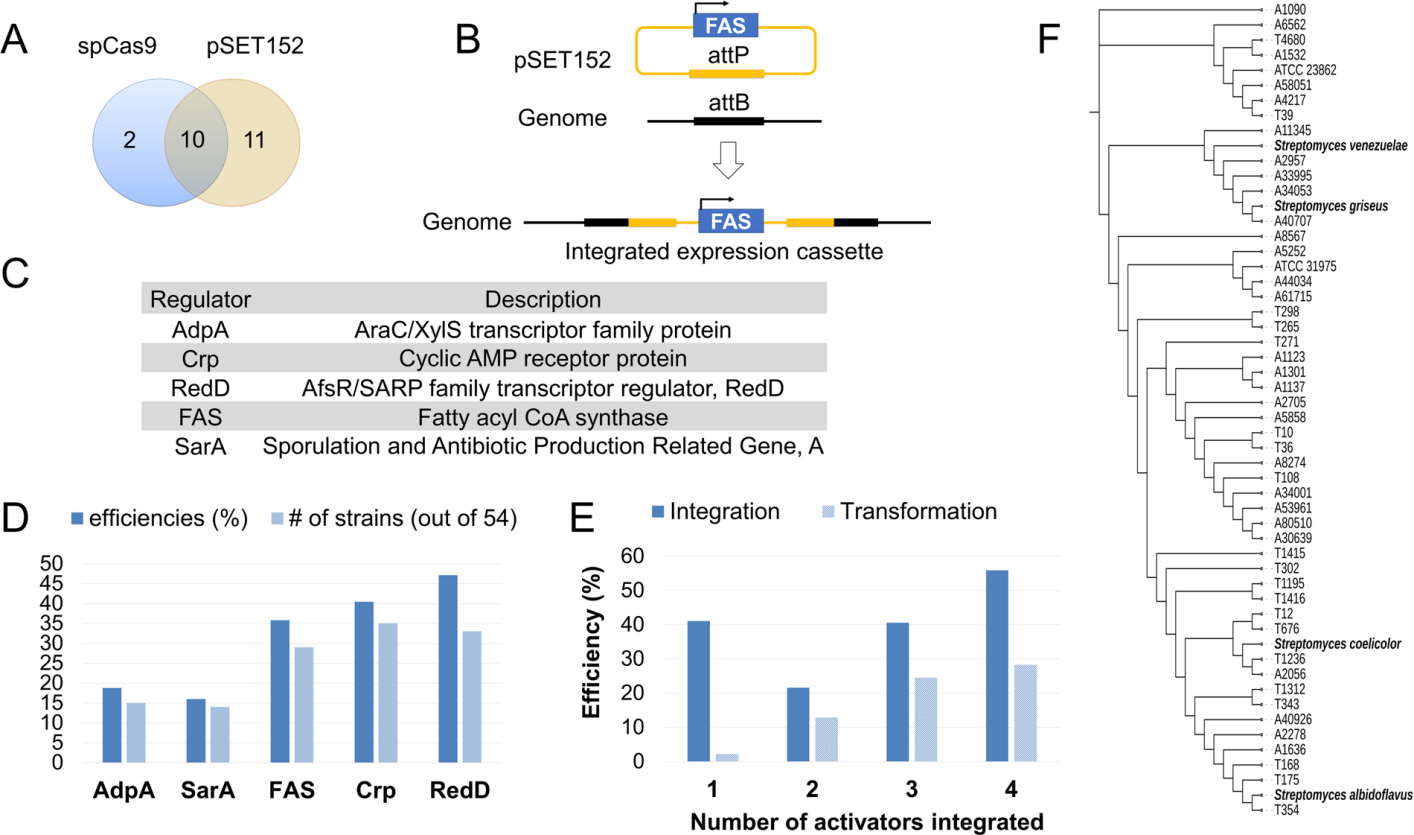
Strain editing. **A** Transformation success rate of 23 unique actinobacterial strains by pSET152 (empty vector) and pCRISP-omyces (no sgRNA, pCRISP-omyces, Addgene #61737). Mutants are screened for presence of plasmids. **B** Schematics of phiC31 integration of FAS overexpression cassette. **C** Table of regulators examined and their abbreviations. **D** Average integration efficiencies of overexpression cassettes in 54 strains respectively to each activator. Y-axes: dark blue bar refers to the average integration efficiency (%) across all strains with the same activator integrated. Integration efficiencies (%) is given by the percentage of correctly integrated mutants over total exconjugants screened for the respective activator per strain (this includes 0%); light blue bar refers to the number of strains with integrated cassette out of 54 strains. **E** Comparison of average integration efficiencies (%, dark blue) and transformation efficiencies (%, light blue) across 54 strains. 54 strains are categorized into the number of activators that were successfully integrated into the strains. 12 strains have 1 activator integrated, 22 strains have 2 activators, 11 strains have 3 activators, and 9 strains have 4 activators. Y-axes: dark blue bar refers to the average integration efficiency (%) across all strains with the same number of activators integrated. Integration efficiency (%) for each strain is calculated as the percentage of correctly integrated mutants over total exconjugants screened for each strain per activator. Transformation efficiencies refer to the percentage of (number of exconjugants observed from integration across 5 activators)/1000 per strain. Average of transformation and integration efficiencies were taken across the different groups of strains. Detailed information can be found in Table S4. (F) Phylogenetic tree construction was as follows. 16S rRNA sequences of 50 strains (Table S1) and 4 model *Streptomyces* (*Streptomyces venezuelae*, *Streptomyces griseus*, *Streptomyces albidoflavus* and *Streptomyces coelicolor*) were aligned using Mafft (v7.505)^71^. The alignment file was used to generate the phylogenetic tree using IQ-TREE (v2.2.0.3)^72^ with default options and visualized using iTOL v5^73^.

Amongst the genetic editing strategies in actinomycetes, CRISPR-Cas mediated editing protocols are highly efficient and have worked consistently^26,27^. In another genetic editing strategy for *Streptomyces*, integrase, derived from actinophage phiC31, utilizes attB sites widespread within actinobacteria for unidirectional recombination^28^. Recent studies have shown phiC31 integrase to also work in Gram-negative microbes^29^, flies^30^, stem cells^31^ and even plants^32^. A key bottleneck for gene editing is the transformation or accessibility of foreign DNA. Thus to compare these two strategies, we first interrogated the transformation efficiencies of CRISPR-Cas (pCRISPomyces-2, Addgene #61737) and phiC31 integrase (pSET152)^28^. Our initial survey of 23 unique actinomycetes strains found that phiC31 integration vector (pSET152) could be integrated by 21 strains, whilst only 12 of these strains successfully incorporated SpCas9 containing pCRISPomyces-2 (Table S2, Fig. 2A). Based on these findings, we opted for phiC31 integration as a reliable and versatile genetic activation strategy for the integration of cassettes that overexpress genes responsible for activating and upregulating secondary metabolite production.

To activate secondary metabolite production, we first generated a library of phiC31 integration plasmids with various overexpression cassettes, where each cassette will consist of 1 “activator” of interest under strong constitutive promoter kasO*p^33^ (Figs. 1A and 2, Table S3). Within the 54 microbial strains examined, the efficiencies at which these five respective overexpression cassettes were integrated varied depending on accessibility of the individual strain and activator (Table S4, Fig. 2D and E). Across the integrations, regulators that are known to adversely affect sporulation seem to have significantly lowered integration efficiencies, possibly due to their impact on growth (Fig. 2D). Successful integration of a single activator into the strain is contingent upon both transformation and integration efficiencies (Fig. 2E). In some cases, only one activator could be integrated, this was often attributed to low transformation efficiencies, with typically less than ten exconjugants observed per integration. Conversely, in strains where two activators could be integrated, transformation efficiencies were at least six times higher on average. As transformation efficiencies and integration efficiencies increased, we also observed a greater likelihood of integrating activators into the strains. Interestingly, we did not encounter any strains that could integrate all five activators. Despite these difficulties, all 54 strains studied have at least one mutant produced after a single round of conjugation. In total, 459 mutants were obtained from integrating 54 strains with 5 regulators. These consisted of 1–15 mutants from a total of 124 unique strain-activator combinations (Table S5), where each mutant would only have one overexpression cassette of an activator integrated.

In our initial analyses, we noticed differences in the diversity and abundance of metabolites among mutants with the same integration cassette (Figs. S1, S2). Despite possible non-specificity of phiC31 integration^34^, we found that among a group of mutants for strain A1123 (Table S1, Fig. S3), only a single integration site was consistently observed. These led us to hypothesize that minor mutations might have occurred via multiple serial transferring of colonies as part of the integration protocol^35,36^, that were subsequently amplified and may contribute to differences in secondary metabolite production between mutants (Figs. S1, S2)^37^. This initial observation from the A1123 set of mutants emphasized the importance of profiling and screening all mutants generated in this study.

### Activated strains have almost doubled metabolite space

**Fig. 3:**
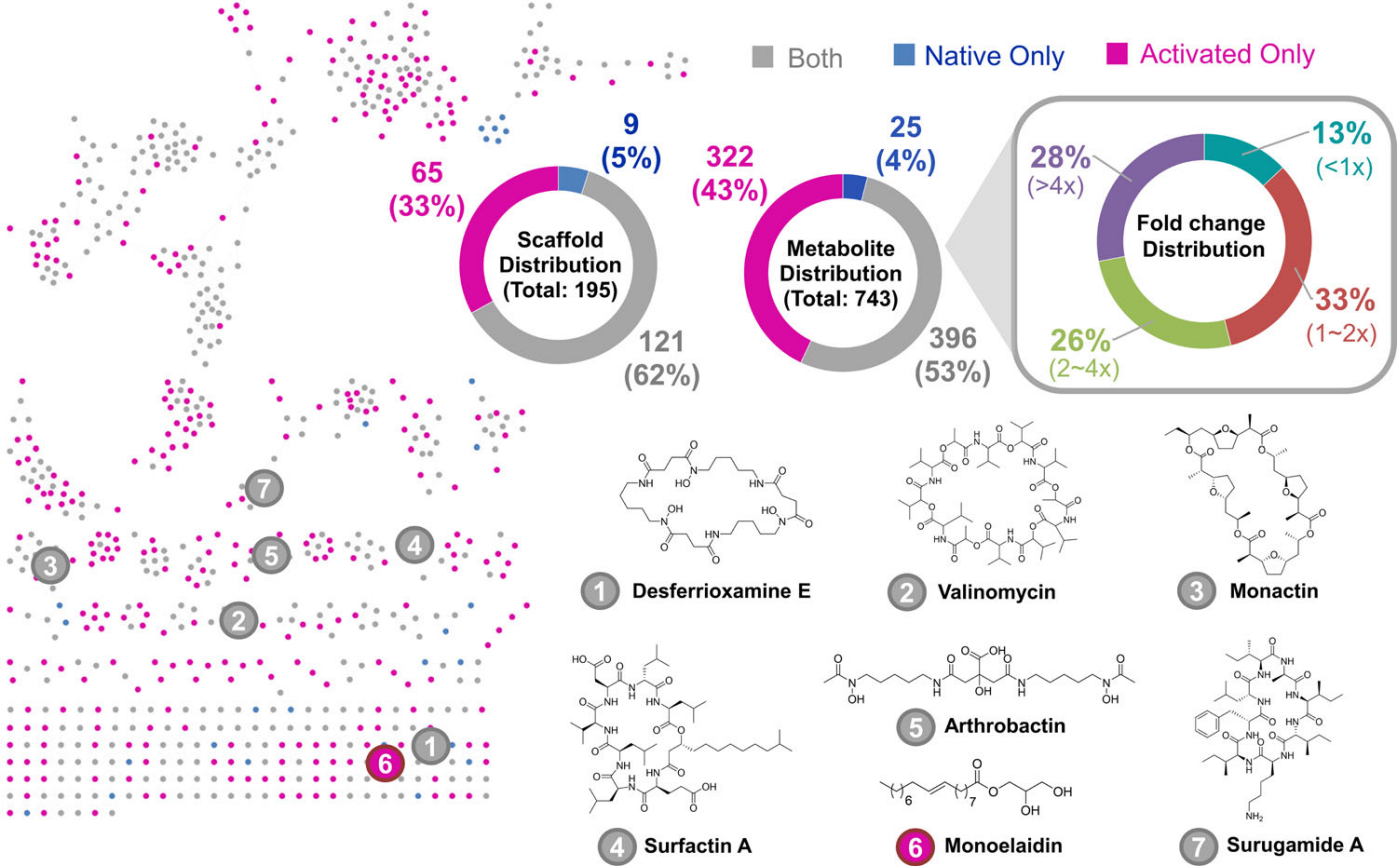
Global natural products social molecular networking (GNPS) analysis of tandem mass spectra of 2138 fermentation extracts from 54 actinobacterial strains and their 459 mutants in 3–5 media. 743 unique metabolites arranged in 69 clusters (with 2 or more metabolites) and 126 orphans were identified (signal intensity > 10^3^). Each cluster and individual orphan were counted as unique scaffolds. Scaffold and metabolite distributions describe the number of scaffolds or metabolites found in native strains only (blue), in activated strains only (magneta), and in both native and activated strains (gray). Fold change distribution of the metabolites depict the fold change (<1x to >4x) of the metabolite signal intensity between the native and activated strains. Chemical structures of metabolites 1-7 were elucidated by matching against GNPS spectral libraries. Surfactin was further verified by NMR characterization (Figs. S34–S39).

To understand metabolite perturbation within activated strains, generated mutants and native strains were fermented in 3-5 media (Tables S6, S7) and the resulting 2138 fermentation extracts analyzed with liquid chromatography-tandem mass spectrometry (LC-MS/MS). Surprisingly, all 124 activator-strains (or 459 mutants) demonstrated enhanced metabolite production with respect to their native strain in at least one media. Comparative MS/MS profile analyses revealed only 37 (1.7%) activated strain extracts (from 15 parent strains) with no new or upregulated metabolites over the parent strain fermented in the same media. Out of these 37 extracts, 21 (57%) were from CA07LB media (Table S7), indicating the importance of media conditions on metabolite production. Global natural products social molecular networking (GNPS)^38^ analysis of the analyzed extracts revealed 743 unique metabolites arranged in 69 clusters (with 2 or more metabolites) and 126 orphan metabolites (Fig. 3). In this context, we consider networked clusters of metabolites and individual orphan metabolites as unique scaffolds. Unique scaffolds have cosine similarities of <0.7 and <6 matched peaks with each other—indicating different fragment ions and disparate core structures. In summary, we observed an approximately 50% increase in unique scaffolds (from 130 to 195) and a 1.8-fold increase in new metabolites due to our multi-pronged activation approach. Specifically, 322 new metabolites were solely detected in the activated strains. While out of the 421 metabolites observed in the native strains, 396 (or 94%) were also present in the activated strains. Overall, this suggests our comprehensive multi-pronged activation approach can effectively enhance NP production, preserving most inherent chemical diversity while nearly doubling the output of unique metabolites under laboratory conditions.

It should be noted that the increase in the proportion of novel scaffolds (~1.5 fold) for these activated strains did not match the larger increase in novel metabolites (~1.8 fold). This suggests that a portion (~20–30%) of the observed novel metabolites produced due to activation were likely analogs of existing scaffolds. Although we cannot rule out that some of these metabolites may be shunt products, intermediates from BGCs, or compounds derived from primary metabolism, the significant expansion of chemical space together with observations of novel bioactivity (*vide infra*) strongly suggests successful upregulation of silent or poorly expressing BGCs by the incorporated regulators. Besides, majority (87%) of the overlapping metabolites produced by both native and activated strains were upregulated (>1x fold change in metabolite signal intensity) with increases in yield of up to 203-fold for certain metabolites. Notably, amongst the 7 characterized metabolites (Fig. 3), many new producers were observed in 5 of the metabolites due to strain activation (Table S8). Significant upregulation of poorly produced metabolites is critical to enable their further characterization and isolation, allowing for more comprehensive interrogation of accessible chemical space.

### Combinatorial effects of media and regulator for maximal chemical space access

**Fig. 4:**
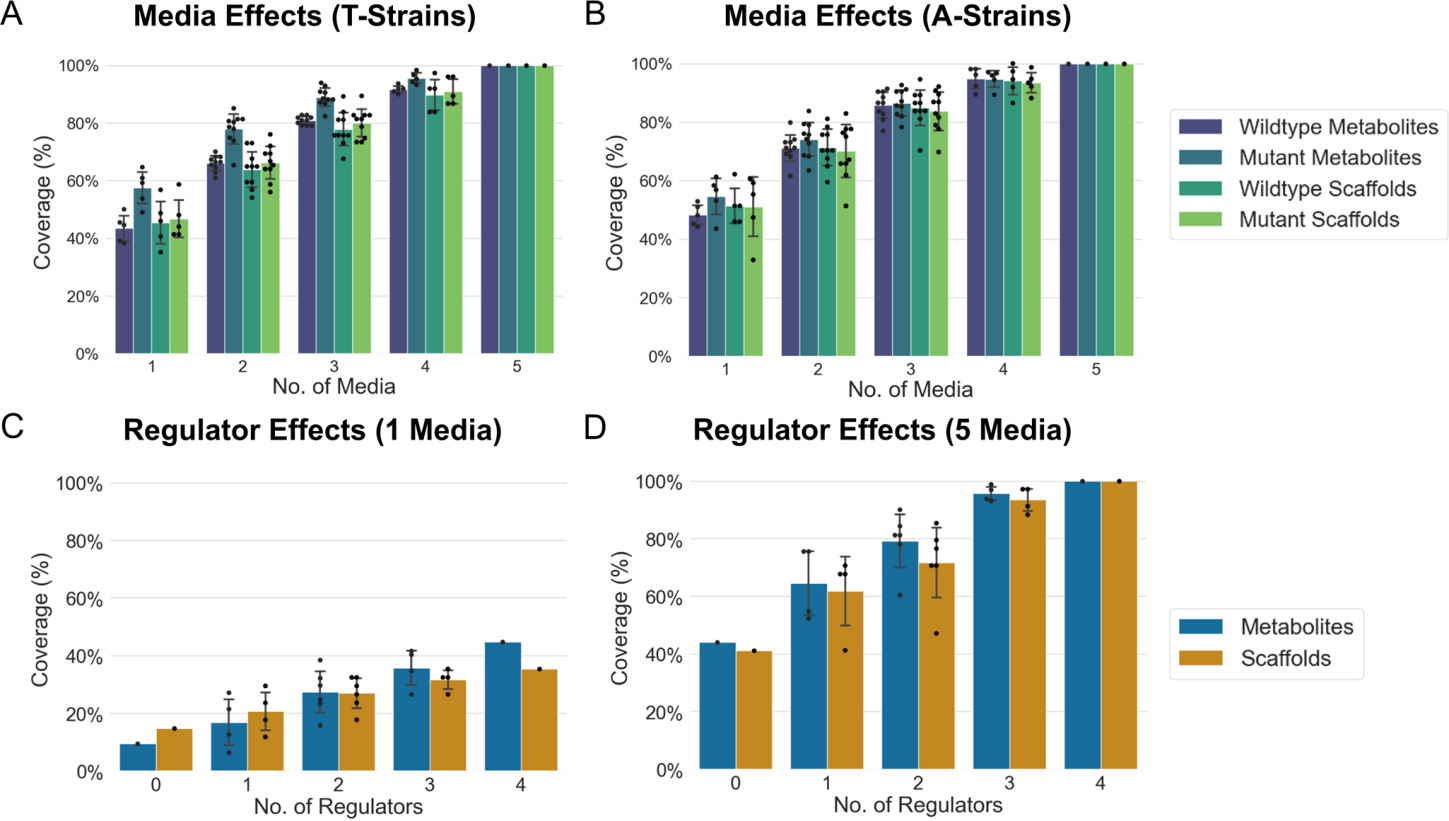
Media and regulator effects on accessible chemical space. Media effects analyses separated by environmental origin and performed on tandem mass spectral data from (**A**) 8 native T-strains of marine origin (T108, T265, T298, T343, T354, T676, T1236, T4680, Table S1) and their 99 mutant clones (Table S5) as well as (**B**) 17 native A-strains of terrestrial origin (A1090, A1123, A1137, A1301, A2056, A2705, A4217, A5252, A8274, A8567, A11345, A33995, A34053, A44034, A58051, A61715, A80510, Table S1) and their 177 mutant clones (Table S5). Regulator effects analyses were performed on tandem mass spectra from (**C**) a single media (CA08LB) and (**D**) across 5 media (CA02LB, CA07LB, CA08LB, CA09LB, CA10LB) were done on 4 strains (A61715, T1312, T1415, T343, Table S1) that have mutants with AdpA, Crp, RedD, or FAS integrated (Table S5). Marine actinomycetes (T1312, T1415, T343) have 40 g/L sea salt added in their media. Coverage refers to the accessible chemical space of metabolites or scaffolds of the native or activated mutant strains detected by liquid chromatography-tandem mass spectrometry averaged across all possible combinations. Coverage (%) is calculated by relative portion of scaffolds or metabolites produced with respect to the maximum observed metabolites or scaffolds in 5 media (wild-type values are relative to wild type in 5 media and mutant values are relative to mutant in 5 media) for media effects or relative with respect to 4 regulators in 5 media for regulator effects. Error bars of 1 standard deviation are given for different combinations of media or regulators. In this context, we have defined unique scaffolds as networked clusters of metabolites and individual orphan metabolites, which have cosine similarities of <0.7 and <6 matched peaks.

Without a priori knowledge of the strains, it was important to employ a “one strain many compounds” (OSMAC) cultivation-based strategy to ensure that cultivation conditions were optimal to meet the strains’ biosynthetic potential. The strains were fermented in 3-5 media (Table S7) with various carbon sources, salts, and trace elements. For marine-derived strains, sea salt (M) was also added to the media. Despite enforced overexpression of integrated regulators through a strong constitutive promoter, cross-strain media analysis affirmed the importance of media optimization (Fig. 4A, B). Accessible chemical space (by metabolites or scaffolds) doubled when the terrestrial (A-strains) and marine (T-strains) were fermented in 5 media compared to 1 media. Due to diminishing returns with the addition of more types of media, a cost-effective OSMAC cultivation-based interrogation could stop at 3 media, which would provide approximately 80% of the accessible chemical space coverage within a strain collection afforded by all 5 media.

An in-depth analysis of the effect of multiple global regulators (AdpA, Crp, RedD, and FAS) in selected microbial strains (A61715, T1312, T1415, T343, Table S1) as a strategy alone and in combination with cultivation-based activation (Fig. 4C) demonstrates their synergy to unlock novel chemical space. A strategy employing only cultivation-based activation or only genetic-based activation gave similar increases in unique metabolites from 9% to 44% or to 36%, respectively. However, when genetic and cultivated-based strategies are employed in combination, there is a significant increase in metabolic potential of 1.5-2.7-fold compared to the individual strategies or >10-fold compared to fermentation of a native strain in a single media. However, significant variance (±11-12%) in chemical space coverage between individual regulators or combinations of 2 regulators (Fig. 4C) highlights the importance of regulator selection. For cost-effective multi-regulator interrogation of strains, a combination of 3 regulators is suggested as it would cover roughly 95% of the metabolite space provided by 4 regulators. Together, our results imply that a combination of activation and cultivation-based strategies is critical in accessing targeted metabolite spaces of interest in a high throughput and generalized manner.

### Is there a universal regulator?

**Fig. 5:**
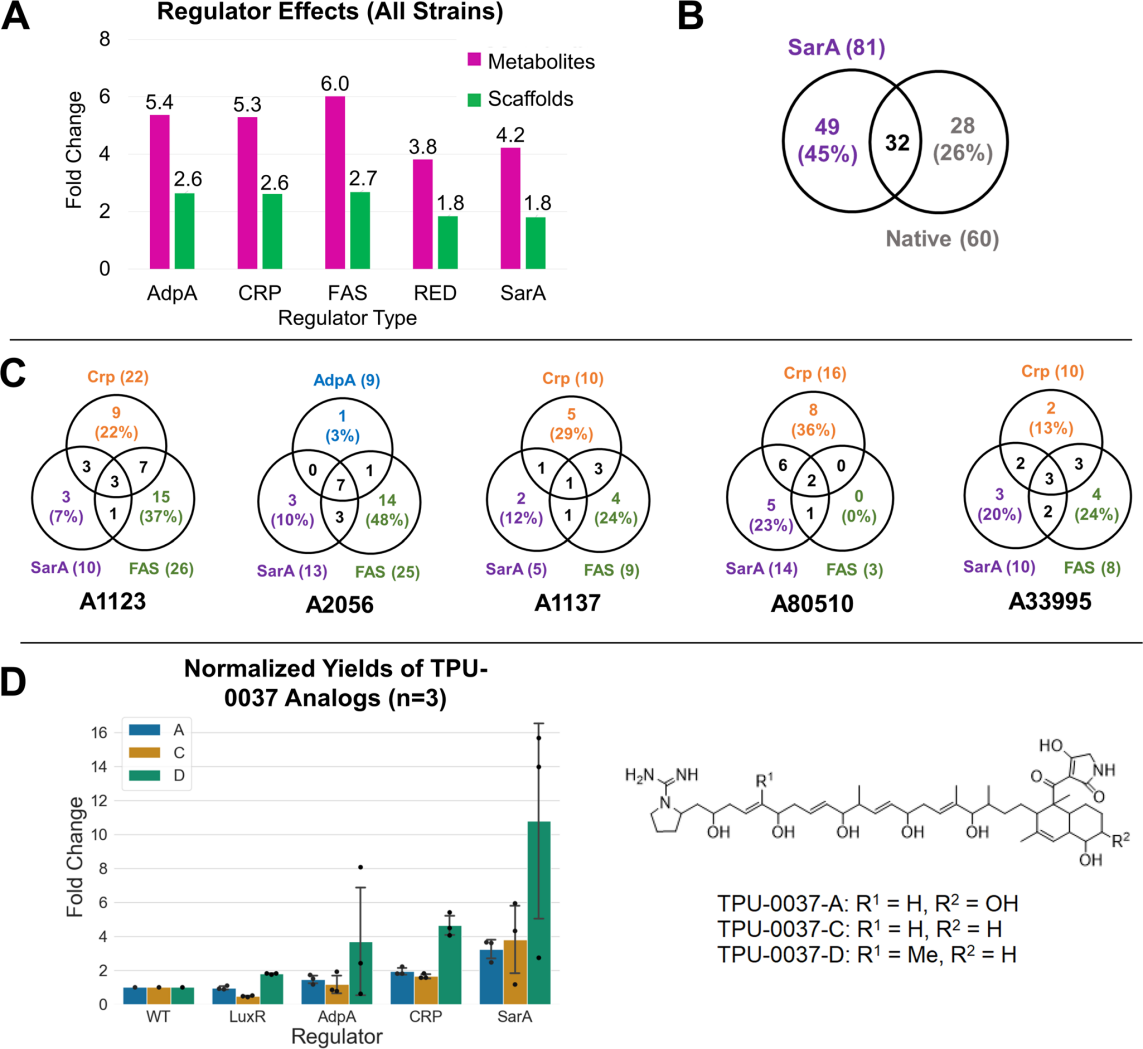
Further analyses on impact of regulators. **A** Regulator effects analyses of tandem mass spectra data from 124 strain-regulator combinations (number of examples: AdpA – 15, CRP – 36, FAS – 27, RedD – 33, SarA – 13). Fold change refers to the ratio of metabolites or scaffolds produced by all mutant strains with that regulator relative to the metabolites or scaffolds produced by their corresponding native strains across all available media. **B** Distribution of unique metabolites produced by the 5 native strains and their SarA mutants across 5 media observed in high resolution mass spectra (MS1). (To note, the sum of the SarA metabolites in Fig. 5C is higher than the total number of SarA metabolites reported here due to 3 duplicate metabolites). **C** Comparison of new metabolites produced under different global regulators SarA, FAS, Crp and AdpA across 5 strains (A1123, A1137, A2056, A33995 and A80510, Table S1) and 84 mutants observed in high-resolution mass spectra (MS1). The number of metabolites observed under each of the 4 global regulators are given in brackets, metabolites in the overlapped sections indicate that they can be induced by either regulator. **D** Upregulation of TPU-0037 analogs in A80510 and respective mutant strains observed in tandem mass spectra, and chemical structures of TPU-0037 analogs. Normalized yields were calculated as the average of *n* = 3 independent mutants. Fold change refers to the cross-media comparison of the highest normalized yields of TPU-0037 analogs in mutant strains relative to the native. Error bars are shown for 1 standard deviation.

A cross-media, cross-strain comparison of the 124 unique strain-regulator combinations (Table S5) suggests regulators that affect native strain environment such as AdpA, Crp, and the regulator that changes substrates flux, FAS, are slightly more prolific than RedD or SarA in increasing the chemical space in terms of metabolites or scaffolds (Fig. 5A). An in-depth analysis of the effect of global regulators (SarA, AdpA, FAS, and Crp) in selected microbial strains (A1123, A1137, A2056, A33995, A80510, Table S1) on their respective metabolites production demonstrate there is varied distribution of unique metabolites across regulators in the 5 strains (Fig. 5C). Together, this emphasizes that regulation is highly strain and activator dependent and thus unfortunately, there is not a “one size fits all” regulator.

We also took this study as an opportunity to evaluate a putative global activator, SarA. In previous studies, SarA was shown to significantly affect actinorhodin production^20,39^. Similar to AdpA^21^, there are also observations of SarA effect on morphological differentiations^21^. Although SarA may not consistently produce as many new metabolites as FAS, Crp or RedD (Fig. 5A), it has allowed us to access unique chemical space not observed in other regulators (Fig. 5B, C). We noted the appearance of 49 unique metabolites (base peak signal intensity abundance ≥ 10^5^) in 5 SarA-activated strains that are not produced by native strains (Fig. 5B), suggesting that SarA overexpression was sufficient to significantly extend chemical space. Amongst the 32 metabolites produced in both native and SarA-activated strains, half of these were upregulated by up to 18-fold (Fig. S4).

To compare our general activation approach with a pathway-specific one, we focused on TPU-0037 analog^40^ production in strain A80510 (Table S1). In A80510 mutants, we noted increased bioactivity against *Staphylococcus aureus* related to increased production of TPU-0037 analogs A, C and D (0. 5D, S5–S8)^40^. As a comparison, we integrated an overexpression cassette of LuxR activator specific to A80510’s lydicamycin BGC under kasO*p (Fig. S9) and evaluated its yields against our other activators. It is noteworthy that with pathway-specific LuxR overexpression, upregulation of production yields was not as substantial as compared with Crp or SarA (~2-fold vs ~4-fold to 12-fold, Fig. 5D). This suggests that although pathway-specific activators may be useful^13^, limitations to NP synthesis and activation could be pleiotropic ones beyond the BGC^41^.

### Discovery of Gram-negative bioactivity for tetramic acid analogs

**Fig. 6:**
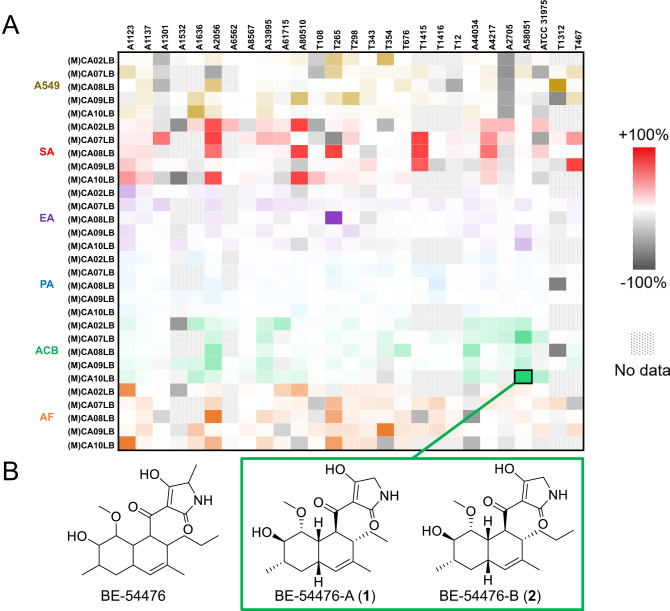
Discovery of new bioactive tetramic acid compounds. **A** Differential bioactivity profiling of microbial strains integrated with FAS expression cassette compared to their native strains. A549 (brown) = cell cytotoxicity against human lung carcinoma cells. SA (red) = antibacterial activity against *Staphylococcus aureus*. EA (purple) = antibacterial activity against *Klebsiella aerogenes*. PA (blue) = antibacterial activity against *Pseudomonas aeruginosa*. ACB (green) = antibacterial activity against *Acinetobacter baumannii*. AF (orange) = antifungal activity against *Aspergillus fumigatus*. **B** Chemical structures of antibiotic BE-54476 and the novel bioactive analogs BE-54476-A (**1**) and BE-54476-B (**2**).

To prioritize for bioactive NP discovery, antimicrobial and cytotoxic abilities of the extracts of fermented strains were also tested. Comparison of the bioactivity profiles of mutants against native strains (Fig. 6A, S5–S6, S10–S11) revealed that expansion of chemical space was accompanied by increased or novel bioactivities under identical media conditions. In FAS integrated mutants of strain A58051 (Table S1), enhanced *Acinetobacter baumannii* growth inhibitory activity was used to identify, isolate, and characterize two new tetramic acid compounds, **1** (named BE-54476-A) and **2** (named BE-54476-B) (Fig. 6). Our multi-pronged activation strategy led to significantly increased production of **1** and **2** compared to the native strain (Fig. S12). Under optimal media conditions, **1** and **2** were isolated at yields of 3.5 mg/L and 2.3 mg/L respectively and fully characterized (Figs. S13–S33, Table S9). **1** and **2** were active against Gram-negative *Acinetobacter baumannii* (ATCC® 19606™) (Table S10, Fig. S32), with MIC_50_ of 9.8 µM and 6.9 µM respectively. Other structurally similar tetramic acid analogs have also been reported to exhibit anti-tumor activity or antimicrobial activity mainly against Gram-positive bacteria^39,42–45^. Interestingly, the absence of a methyl group from BE-54476 is sufficient to produce Gram-negative bioactivity in such scaffolds. As an indication of the uniqueness of these metabolites, high-resolution mass spectrometry scans among ~2 K actinomycetes within the NOL Singapore collection yielded no other evidence of compounds **1** and **2**.

## Conclusion

The comprehensive study presented here demonstrates a versatile and effective approach for natural product discovery through multi-pronged activation. Our highly flexible and robust one-step integrase-based protocol has achieved successful integration of a library of “activators” across a diverse set of 54 actinomycete strains. Using a combination of media and strain engineering, we have observed remarkable upregulation of secondary metabolites, including near-doubling of accessible metabolite space along with consistent upregulation of these secondary metabolites by up to 203-fold—within over 98% of the tested activated strain fermentation extracts under controlled laboratory conditions. Regarding the challenges in engineering native strains, it is important to highlight that among a possible 270 permutations (comprising 54 strains and 5 activators), we managed to generate functional engineered strains of only 124 activator-strain combinations after a single round of conjugation. However, all activator-strain combinations have enhanced metabolite production (fold change >1) with respect to its native strain in at least one media. In contrast, when employing heterologous engineering methods, although refactoring and engineering techniques may be highly efficient, the resulting strains do not consistently yield the anticipated metabolites^14,15,46,47^. The insights gleaned from this study also extend to analyses on the impact of “activators” and growth media on strain enhancement. From this work, we have identified the minimal conditions for maximizing accessible chemical space, allowing for resource-efficient interrogation of the chemical potential of strains. Finally, the application of our multi-pronged activation approach has enabled our discovery of new tetramic acid analogs displaying the first example of gram-negative bioactivity within their compound class. In the current landscape of increasingly accessible, rapidly improving automation^48^ and genetic tools, our approach provides the blueprint needed for a streamlined, accelerated, and scalable strategy to effectively and permanently enhance native strains for NP production, interrogate cryptic BGCs, and circumvent time-consuming traditional requirements of precise engineering, refactoring, and assembly^14,15,49,50^. Moving forward, we envision that our multi-pronged activation strategy, together with high throughput data analytics, will serve as key enabling technologies to drive actionable insights that enable the full utilization of Nature’s chemical repertoire.

## Materials and methods

### Vectors containing integration cassette

Overexpression cassettes consist of the respective genes (Table S3) under strong constitutive kasO* promoter. The integration plasmid was derived by cloning the overexpression cassette into pSET152^28^. More details are given in Supporting information 2 (Table S11 and Figs. S41–S46). The gene of interest was first PCR amplified from *Streptomyces coelicolor* or *Streptomyces lividians* and cloned into a vector carrying the kasO* promoter via Gibson Assembly. The gene fragment together with the kasO* promoter was then ligated into pSET152 via the XbaI cut site to give the integration plasmids (Table 1).

**Table 1:** Plasmids used in this study.

Plasmids	Description	Origin
pSET152	Site-specific integration vector, contains *phi*C31 integrase, attP, and oriT (RK2), apramycin-resistant cassette	^28^
pCRP63	pSET152- kasO*p-SCO2792 (AdpA)	This work
pCRP65	pSET152- kasO*p-SCO3571 (Crp)	This work
pCRP67	pSET152- kasO*p-SCO4069 (SarA)	This work
pCRP178	pSET152- kasO*p-SCO6196 (FAS)	This work
npC697	pSET152- kasO*p-SLIV09220 (RedD)	This work

### Conjugation

WM6026 and WM3780 *E.coli* donor strains (Table 2) were used to perform conjugation experiments with R2 agar without sucrose. Spore preparation and conjugation protocols follow Zhang et al.^51^. Apramycin selection was used to select for integrated mutants. The genetically integrated mutants were screened via PCR using primers flanking the kasO* promoter and the overexpression cassettes (Table 3, Fig. S40) with wild-type genome as negative control. Sanger sequencing was used to confirm the integration of the overexpression cassettes.

**Table 2:** Strains used in this study.

Strains	Description	Origin
OmniMAX™	*Escherichia coli* for plasmid preparation and cloning	ThermoFisher Scientific, USA
WM6026	Auxotrophic *Escherichia coli* that requires diaminopimelic acid supplementation, for conjugation with *Streptomyces*	^69^
WM3780	*DNA methylase-deficient Escherichia coli*, for conjugation with *Streptomyces*	^70^

**Table 3:** Primers used in screening of exconjugants.

Primers ID	Sequence	Primer region
1	tgttcacattcgaacggtctctgc	kasO*p N-terminus
2	acacgactggatactgacttttcacactagtgaacgccggacgggctgagtgtg	SCO2792 (AdpA) C-terminus
3	acacgactggatactgacttttcacactagtcaggggcgcgctccgtaccg	SCO6196 (FAS) C-terminus
4	acacgactggatactgacttttcacactagtcagcgggagcgcttggccagtc	SCO3571 (Crp) C-terminus
5	acacgactggatactgacttttcacactagtcagaccagacgcaccggcttctcg	SCO4069 (SarA) C-terminus
6	acagctatgacatgattacgaattcgatcaggcgctgagcaggctggtgtc	SLIV09220 (RedD) C-terminus

### Fermentation and extraction of terrestrial and marine actinomycetes

Wild-type strains and edited mutants were cultured on ISP2 plates [malt extract 10 g/L, Bacto yeast extract 4 g/L, glucose 4 g/L, Bacto agar 20 g/L] at 28 °C for 5 days. Three agar plugs of 5 mm diameter from the culture plate were then used to inoculate into 250 mL Erlenmeyer flasks each containing 50 mL SV2 seed media [glucose 15 g/L, glycerol 15 g/L, soya peptone 15 g/L, calcium carbonate 1 g/L, pH 7.0] and incubated for 4 days at 28 ^o^C, with shaking at 200 rpm. A volume of 2.5 mL of the homogenized seed cultures were then inoculated into 250 mL Erlenmeyer flasks each containing 50 mL of ferment medium, CA02LB, CA07LB, CA08LB, CA09LB or CA10LB (Table S7). Marine actinomycetes strains were fermented in the same media with the addition of 40 g/L sea salt. All the cultures were fermented at 28 °C for 9 days shaking at 200 rpm with 50 mm throw. At the end of the incubation periods, cultures were freeze dried. The lyophilized samples were extracted overnight with methanol. The extract mixture was passed through cellulose filter paper (Whatman Grade 4, 1004-185) and the filtrate was then dried using rotary evaporator. Media compositions are given in Table S7.

### Liquid Chromatography-Tandem Mass Spectrometry (LC-MS/MS)

The extracts were analyzed on an Agilent 1290 Infinity LC System coupled to an Agilent 6540 accurate-mass quadrupole time-of-flight (QTOF) mass spectrometer. 5 µL of extract was injected into a Waters Acquity UPLC BEH C_18_ column, 2.1 ×50 mm, 1.7 µm. Mobile phases were water (A) and acetonitrile (B), both with 0.1 % formic acid. The analysis was performed at flow rate of 0.5 mL/min, under gradient elution of 2% B to 100% B in 8 min. Both MS1 and MS/MS data were acquired in positive electrospray ionization (ESI) mode. The typical QTOF operating parameters were as follows: sheath gas nitrogen, 12 L/min at 325 °C; drying gas nitrogen flow, 12 L/min at 350 °C; nebulizer pressure, 50 psi; nozzle voltage, 1.5 kV; capillary voltage, 4 kV. Lock masses in positive ion mode: purine ion at *m/z* 121.0509 and HP-0921 ion at *m/z* 922.0098.

### Global Natural Products Social Molecular Networking (GNPS)^38^

MSConvert v3.0.22198-0867718 from Proteowizard^52^ was used for initial processing of raw liquid chromatography-tandem mass spectrometry (LC-MS/MS) data into open-source mascot generic format (.mgf). All tandem mass spectra (MS/MS) signals with intensity values below 1000 signal intensity were removed as background correction. Classical molecular networking was performed on the MS/MS spectra using the online workflow from the GNPS website (http://gnps.ucsd.edu, accessed November 2022). All peaks in a ±17 Da around the precursor ion mass were deleted to remove residual precursor ions, and peaks not in the top 6 most intense peaks in a ±50 Da window were filtered out. The precursor ion mass tolerance was set to 0.02 Da and an MS/MS fragment ion tolerance of 0.02 Da. A network was then created where edges were filtered to have a cosine score above 0.7 and more than 6 matched peaks. Further, edges between two nodes were kept in the network if and only if each of the nodes appeared in each other’s respective top 10 most similar nodes. Finally, the maximum size of a molecular family was set to unlimited. The spectra in the network were then searched against GNPS’ spectral libraries. The library spectra were filtered in the same manner as the input data. All matches kept between network spectra and library spectra were required to have a score above 0.7 and at least 6 matched peaks.

Metabolites **1**–**7** were elucidated by matching against GNPS spectral libraries. Surfactin was further verified by NMR characterization (Figs. S34–S39). TPU-0037 analog, TPU-0037-A was previously isolated from A80510 and its chemical structure was confirmed by comparison of NMR data with literature values^40^, related analogs TPU-0037-C and TPU-0037-D were putatively assigned through high resolution mass spectrometry (HRMS) matching with literature values^40^ and molecular networking (Figs. S7–S8).

### Metabolite production fold change comparison between wildtype and mutated strains

MSConvert v3.0.22198-0867718 from Proteowizard^52^ was used for initial processing of raw liquid chromatography-tandem mass spectrometry (LC-MS/MS) data into open-source mascot generic format (.mgf). All tandem mass spectra (MS/MS) signals with intensity values below 1000 signal intensity were removed as background correction. Metabolite yields from each fermentation extract were referenced from the converted mgf files by comparing the total ion current (TIC) abundance of all sample MS/MS spectra with precursor mass within 0.02 Da and retention time within 0.4 min of the mean values of the unique metabolites identified from Global Natural Products Social Molecular Networking (GNPS) molecular networking^38^ online workflow from the GNPS website (http://gnps.ucsd.edu, accessed November 2022) and taking the highest TIC abundance for each unique metabolite present. Only 359 out of the 396 metabolites identified by GNPS as present in both native and activated strains were identified via this workflow. As each strain were fermented in multiple media, the highest value across all fermentation media for each strain was taken to represent the metabolite yield for that unique metabolite and strain combination. Strains were separated into 2 groups—(i) wild-type strains, and (2) mutated (activated) strains. As multiple strains were observed to produce identical metabolites, the highest metabolite yields across all strains within each group were taken to represent that group’s yield of a given metabolite. These representative metabolite yields for each unique identified metabolite were then compared between the 2 groups with the following formula:

$${Fold}\,{Change}=\frac{{Metabolite}\,{Yield}\, ({Mutant})}{{Metabolite}\,{Yield}\, ({Wildtype})}$$

### Metabolite coverage comparisons (media/activator effects)

Unique metabolites from each fermentation extract were identified and assigned via the Global Natural Products Social Molecular Networking (GNPS) molecular networking^38^ online workflow from the GNPS website (http://gnps.ucsd.edu, accessed November 2022) *vide supra*. Networked clusters of metabolites and orphan (unconnected singular) metabolites were considered as unique scaffolds. Unique scaffolds have cosine similarities of <0.7 and <6 matched peaks when compared against any other unique scaffold. New metabolites due to media/activator effects were identified via spectral comparison of LC-MS/MS between wild-type and modified fermentation extracts. In this way, media components and their respective degradation products present in both would be automatically ruled out as potential false positives.

### Biological assays

The minimum inhibition concentration (MIC) and minimum bactericidal/fungicidal concentration (MBC/MFC) of the isolated compounds against a panel of microbial pathogens were determined using the microbroth dilution method. This was conducted following the Clinical Laboratory Standards Institute (CLSI) guidelines, with the following modifications: antibacterial assays were carried out with *Acinetobacter baumannii* (ATCC® 19606™), *Klebsiella aerogenes* (ATCC® 13048™), *Pseudomonas aeruginosa* (ATCC® 9027™) and *Staphylococcus aureus* Rosenbach (ATCC® 25923™) at 5.5 × 10^5^ cells/mL. Antifungal assays were performed with *Aspergillus fumigatus* (ATCC® 46645™) at 2.5 × 10^4^ spores/mL. Gentamicin (Gibco) and amphotericin (Sigma-Aldrich) were used as the assay controls for the antibacterial and antifungal assays respectively. For MIC determination, the bacterial cells were incubated together with the isolated compounds at 37 °C for 24 h; and at 25 °C for 72 h for the fungal spores. Optical density at 600 nm was then measured using a microplate reader (Tecan Infinite® M1000 Pro) to assess the inhibitory effect of the compounds on microbial growth. Following that, MBC/MFC were then evaluated by transferring 5 µL of the treated culture into fresh media in 384-well microtiter plates. The plates were incubated under the same conditions, and MBC/MFC was determined by measuring the optical density at 600 nm. Primary bioactivity assays were performed in duplicates whilst assays were performed in triplicate for isolated compounds.

For the mammalian cell cytotoxicity assay, A549 human lung carcinoma cells (ATCC® CCL-185™) were seeded at 3.3 × 10^4^ cells/mL. The cells were treated with the compounds for 72 h and incubated at 37 °C in the presence of 5% CO_2_. Puromycin (Sigma-Aldrich) was used as the assay control for cytotoxicity testing. To assess the cytotoxic effect of the compounds on the cells, the microplates were incubated with PrestoBlue™ cell viability reagent (ThermoFisher Scientific, USA) for 2 h, followed by fluorescence reading at excitation 560 nm and emission 590 nm. The analysis of antimicrobial and cytotoxicity activity for their IC_50_ values were carried out with the GraphPad Prism program (GraphPad Software, CA).

### Bioactivity heat maps

Comparison of bioactivity in the 6 biological assays between extracts obtained from mutant-parent pairs. The highest biological assay %inhibition value from all clones of a given activator-strain combination across all media was taken as a representative value to calculate the bioactivity difference from wild type by the following equation:

$$	{Mutant}\,{Assay} \, \% {Inhibition}\,{Value}-{Native}\,{Assay} \, \% {Inhibition}\,{Value}\\ 	 ={Difference}$$

Where the highest biological assay %inhibition value from the native strain extracts across all media from the parent strain of that given activator-strain combination was taken as the representative value for “Native Biological Assay Value”. This was done to represent the perturbation and triggering of antibiotics production due to our multi-pronged activation approach. Bioactivity heat maps were constructed with a 3-color palette with the following set-points:

$${Dark}\,{Grey}=-100\, {Difference}$$

$${White}=0\, {Difference}$$

$${Colour}\, (A549 	={Brown},{SA}={Red},{EA}={Purple},{PA}={Blue},{ACB}\\ 	={Green},{AF}={Orange})=+100\, {Difference}$$

A549 (brown) = cell cytotoxicity against human lung carcinoma cells. SA (red) = antibacterial activity against *Staphylococcus aureus*. EA (purple) = antibacterial activity against *Klebsiella aerogenes*. PA (blue) = antibacterial activity against *Pseudomonas aeruginosa*. ACB (green) = antibacterial activity against *Acinetobacter baumannii*. AF (orange) = antifungal activity against *Aspergillus fumigatus*.

A -100 to +100 range was set to allow finer differentiation of biological activity due to activation. However, values of less than -100 and more than +100 are present.

### BE-54476-A and BE-54476-B: Large scale fermentation and extraction

A100020 mutant was grown on Bennet’s agar (Himedia, M694) plates at 28 °C for 5 days. Three agar plugs of 5 mm diameter from the culture plate were then used to inoculate the mutants into 250 mL Erlenmeyer flasks containing 50 mL of SV2 seed media each and incubated for 4 days at 28 °C, with shaking at 200 rpm. Homogenized seed cultures (2.5 mL) were then inoculated into 250 mL Erlenmeyer flasks each containing 50 mL of ferment medium, CA07LB. All the cultures were fermented at 28 °C for 9 days shaking at 200 rpm with 50 mm throw. At the end of the incubation periods, cultures were harvested and freeze dried. The lyophilized cultures were extracted overnight with methanol. The extract mixture was passed through cellulose filter paper (Whatman Grade 4, 1004-185) and the filtrate was then dried using rotary evaporator.

### BE-54476-A and BE-54476-B: Compound isolation

The dried extracts obtained from 1 L of fermentation were combined and partitioned with 240 mL CH_2_Cl_2_/MeOH/H_2_O in a ratio of 1:1:1. The aqueous MeOH layer was washed with 80 mL CH_2_Cl_2_ (x2) and dried under reduced pressure using a Buchi rotary evaporator. The dried crude extract (6 g) was soaked and resuspended with 20 mL MeOH, sonicated for 5 min and centrifuged to separate the insoluble from the soluble fractions. The supernatants were transferred to 50 mL round bottom flaks and dried using a Buchi rotary evaporator. The dried enriched samples (427 mg) were dissolved in 2.5 mL MeOH, centrifuged and the supernatants were then subjected to C_18_ reversed-phase preparative HPLC purification under the following conditions (solvent A: H_2_O + 0.1% HCOOH, solvent B: MeCN + 0.1% HCOOH; flow rate: 30 mL/min, gradient conditions: 90:10 isocratic for 5 min; followed by 10% to 45% of solvent B over 15 min, 45% to 75% of solvent B over 38 min, 75% to 100% of solvent B over 2 min, and finally isocratic at 100% of solvent B for 12 min) to give 3.5 mg of BE 54476-A (**1**) and 2.3 mg of BE 54476-B (**2**).

### BE-54476-A and BE-54476-B: Chemical analysis

A JASCO P-2000 digital polarimeter was used for specific rotations measurement. NMR spectra were collected using Bruker DRX-400 NMR spectrometer with Cryoprobe. 5-mm BBI (^1^H, G-COSY, multiplicity-edited G-HSQC, and G-HMBC spectra) or BBO (^13^C spectra) probe heads equipped with z-gradients. The ^1^H and ^13^C NMR chemical shifts were referenced to the residual solvent peaks for MeOH-*d*_4_ at δ_H_ 3.31 and δ_C_ 49.0 ppm. For analytical HRMS analysis, an Agilent UHPLC 1290 Infinity coupled to Agilent 6540 accurate-mass quadrupole time-of-flight (QTOF) mass spectrometer equipped with a splitter and an ESI source was used to conduct HPLC-LCMS. For over 8.6 min, under standard gradient condition of 98% water with 0.1% formic acid to 100% acetonitrile with 0.1% formic acid, the analysis was performed with a Acquity UPLC BEH C_18_ 2.1 × 50 mm, 1.7 µm column at flow rate of 0.5 mL/min. Agilent 1260 Infinity Preparative-Scale LC/MS Purification System and Agilent 6130B single quadrupole mass spectrometer with Agilent 5 Prep-C_18_ column (100 × 30 mm, 5 µm, 100 Å) was used to perform preparative HPLC experiment. All solvents for chromatography, specific rotations, and UV were Fisher Chemical HPLC or LCMS grade.

### BE-54476-A and BE-54476-B: structural characterization

BE-54476-A was isolated as a brown amorphous powder and was assigned the molecular formula C_20_H_29_O_5_N following analysis of the (–)-HRESIMS data (*m/z* 362.1968 [M–H]^–^, calcd for C_20_H_8_O_5_N, 362.1973). Optical rotation and UV-Vis data of BE-54476-A are as follows:$\,{[{{{{{\rm{\alpha }}}}}}]}_{D}^{23}$ + 50.8 (c 0.5, MeOH); UV (MeCN/H_2_O) λ_max_ (%) 220 (100%), 285 (77%) nm. The ^1^H NMR spectrum of BE-54476-A showed the signals corresponding to four methyl groups (δ_H_ 0.75, 1.04, 1.63, and 3.26), three methylenes (δ_H_ 1.15/1.65, 1.29, and 3.75), and eight methine protons (δ_H_ 1.39, 2.12, 2.57, 2.76, 2.99, 3.43, 4.06, and 5.61). The ^13^C NMR and edited HSQC spectra exhibited twenty resonances including four methyls, three methylenes, eight methines, and five non-protonated carbons. The UV absorptions maxima at 220 and 285 nm^53,54^ were typical for a tetramic acid moiety, which was further supported by the characteristic broad ^13^C NMR signals at δ_C_ 104.5, 178.2, 196.0, and 201.8^55,56^. The broad ^13^C peaks were most likely due to keto-enol tautomerism of the tetramic acid moiety^56^. The remaining structure of BE-54476-A was assigned based on COSY and HMBC data (Figs. S19-S20). Analysis of COSY spectrum provided the fragments of H-5/H-6/H_2_-7/H-8/H-9/H-10/H-11/H-2/H-3 and H_3_-15/H-8 (Fig. S20). These data together with HMBC correlations from H-5 to C-6, C-7, and C-11, from H-10 to C-6 constructed a partial structure of decalin. Further, ^1^H-^1^H COSY spin-system of H-3/H_2_-12/H_3_-13 along with HMBC cross-peak from H_2_-12 to C-3 confirmed the location of CH_3_-CH_2_- substituent at C-3. In addition, HMBC correlation from H_3_-14 to the olefinic carbon at δ_C_ 135.4 placed a methyl group at C-4. The position of a methoxy group at C-10 was evidenced by HMBC correlation from the singlet methyl resonance at δ_H_ 3.75 to an oxygenated carbon at δ_C_ 88.7. The presence of a tetramic acid moiety at C-2 was further confirmed by HMBC correlations from H_2_-5’ (δ_H_ 3.75) to C-2’ and C-4’ and by considering the deshielded chemical shift of H-2 (δ_H_ 4.06), which was typical of tetramic acids system^44,56^. Finally, a hydroxy group was located at C-9 by considering the molecular formula of BE-54476-A and the chemical shift of CH-9 (δ_H_/δ_C_ 3.43/75.1).

The relative configuration of BE-54476-A was assigned by analysis of NOESY data (Fig. S21) and ^1^H-^1^H coupling constants, as well as comparison of reported values of NMR data of similar decalin-containing tetramic acid compounds. For instance, the chemical shift of the decalin ring junction signals H-11 (δ_H_ 2.76) and H-6 (δ_H_ 2.12) were consistent with other *cis*-decalin tetramic acid analogs^44,57–60^, when compared to the *trans*-fused congeners^55,61–64^. A large coupling constant of 9.8 Hz between H-9 and H-10 indicated that these protons were axially oriented. NOESY correlations between H-2 and H-9 as well as between H-9 and H-7_ax_ [δ_H_ 1.15, ddd (12.3, 12.3, 12.3)] suggested these protons to be on the same side of the molecule (*α*-oriented). Finally, further NOESY cross-peaks between H-6 and H-8 as well as between H-3 and H-11 supported a *β*-configuration of these protons. Hence, the structure of BE-54476-A was established as a new tetramic acid derivative similar to a known compound BE-54476 and named BE-54476-A.

BE-54476-B was also isolated as a brown amorphous powder and assigned the molecular formula C_20_H_31_O_5_N by (–)-HRESIMS data (*m/z* 376.2132 [M–H]^–^, calcd for C_20_H_30_O_5_N, 376.2129). Optical rotation and UV-Vis data of BE-54476-B are as follows: ${[{{{{{\rm{\alpha }}}}}}]}_{D}^{23}$ + 28 (c 0.2, MeOH); UV (MeCN/H_2_O) λ_max_ (%) 222 (100%), 286 (83%) nm. The ^1^H and ^13^C NMR data as well as UV spectrum of BE-54476-B were similar to those of BE-54476-A. Detailed analysis of NMR and MS data of BE-54476-B suggested that it has an additional –CH_2_– group. 2D NMR data analysis indicated that BE-54476-B had a propyl moiety at C-3 instead of an ethyl group in BE-54476-A. This was further supported by HMBC correlations from a triplet methyl at δ_H_ 0.81 (13-CH_3_) to C-12 and C-13 as well as COSY correlations for 13-CH_3_/H_2_-13/H_2_-12/H-3. The same relative configuration previously determined for BE-54476-A was also assigned for BE-54476-B following analysis of NOESY spectrum and ^1^H-^1^H coupling constants. Thus, the structure was given the trivial name BE-54476-B.

Full details of the NMR assignments of BE-54476-A and BE-54476-B are provided in Table S9.

### BE-54476-A and BE-54476-B: biological assays

The minimum inhibition concentration (MIC) and minimum bactericidal/fungicidal concentration (MBC/MFC) of the isolated compounds against a panel of microbial pathogens were determined using the microbroth dilution method. This is done according to the Clinical Laboratory Standards Institute (CLSI) guidelines, with the following modifications: antibacterial assays were carried out with *Acinetobacter baumannii* (ATCC® 19606™), *Klebsiella aerogenes* (ATCC® 13048™), *Pseudomonas aeruginosa* (ATCC® 9027™) and *Staphylococcus aureus* Rosenbach (ATCC® 25923™) at 5.5 × 10^5^ cells/mL. Antifungal assays were performed with *Aspergillus fumigatus* (ATCC® 46645™) at 2.5 × 10^4^ spores/mL. Gentamicin (Gibco) and Amphotericin (Sigma-Aldrich) were used as the assay controls for the antibacterial and antifungal assays respectively. For MIC determination, the bacterial cells were incubated together with the isolated compounds at 37 °C for 24 h; and at 25 °C for 72 h for the fungal spores. Optical density at 600 nm was then measured using a microplate reader (Tecan Infinite® M1000 Pro) to assess the inhibitory effect of the compounds on microbial growth. Following that, MBC/MFC were then evaluated by transferring 5 µL of the treated culture into fresh media in 384-well microtitre plates. The plates were incubated under the same conditions, and MBC/MFC were determined by measuring the optical density at 600 nm. All assays were performed in triplicates to ensure reproducibility.

For the mammalian cell cytotoxicity assay, A549 human lung carcinoma cells (ATCC® CCL-185™) were seeded at 3.3 × 10^4^ cells/mL. The cells were treated with the compounds for 72 h and incubated at 37 °C in the presence of 5% CO_2_. Puromycin (Sigma-Aldrich) was used as the assay control for cytotoxicity testing. To assess the cytotoxic effect of the compounds on the cells, the microplates were incubated with PrestoBlue™ cell viability reagent (ThermoFisher Scientific, USA) for 2 h, followed by fluorescence reading at excitation 560 nm and emission 590 nm. The analysis of antimicrobial and cytotoxicity activity for their IC_50_ values were carried out with the GraphPad Prism program (GraphPad Software, CA).

### Genomic analyses of A1123 integration mutants

In order to sequence genomes, a library was created for using 10 ng of genomic DNA per mutant (plexWell™ 96, Massachusetts, USA). The library was sequenced using the 2 × 151 bp paired-end protocol on an Illumina HiSeq 4000 platform. Breakpoints in the genome is located by first aligning the sequenced reads to the reference genome and npC697 using BWA^65^. This is followed by filtering using SAMtools^66^ using the -F 14 flag to locate paired end reads where one of the mate reads mapped to npC697 whereas the other read mapped to the reference genome. These breakpoint crossing reads are collected across samples noting the genomic location in both the reference genome and npC697. As a quality control measure, at least 50 such breakpoint reads are required before considered a valid breakpoint. Results are subsequently visually confirmed by IGV^67^.

### Statistics and reproducibility

In this study, we tested all the available biological replicates in 3- 5 fermentation media. Each biological replicate underwent independent cell culture and fermentations, ensuring unbiased processing and reproducibility. The full description of the biological replicates and their corresponding fermentation media experiments are given in Table S6. Analyses were performed in Microsoft Excel (Microsoft Office 365—version 2203—build 15028.20204).

### Reporting summary

Further information on research design is available in the Nature Portfolio Reporting Summary linked to this article.

### Supplementary information

Supplemental material

Supplementary Tables 1-6

Description of Additional Supplementary Data

Numerical source data for Figure 2

Numerical source data for Figure 3

Numerical source data for Figure 4

Numerical source data for Figure 5

Numerical source data for Figure 6

Reporting Summary

## Data Availability

Numerical source data for Figs. 2–6 and supplementary tables S1–S6 can be found as supplementary data files (e.g., Figure2_Data.xlsx, Supplementary_Data_Tables_S1_to_S6.xlsx). A copy of this supplemental data set consisting of the numerical source data for Figs. 2–6 and supplementary tables S1–S6 has also been made available on figshare^68^. The full LC-MS/MS dataset^24^ from this work is available via the Mass Spectrometry Interactive Virtual Environment (MassIVE) repository (accessed via: https://massive.ucsd.edu/) as a MassIVE Dataset with accession number MSV000092237^24^.

## References

[1] González-Manzano S, Dueñas M (2021). Applications of natural products in food. Foods.

[2] Yan Y, Liu Q, Jacobsen SE, Tang Y (2018). The impact and prospect of natural product discovery in agriculture. EMBO Rep..

[3] Shen B (2015). A new golden age of natural products drug discovery. Cell.

[4] Newman DJ, Cragg GM (2016). Natural products as sources of new drugs from 1981 to 2014. J. Nat. Prod..

[5] Atanasov AG (2021). Natural products in drug discovery: advances and opportunities. Nat. Rev. Drug Discov..

[6] Blin K (2019). antiSMASH 5.0: updates to the secondary metabolite genome mining pipeline. Nucleic Acids Res..

[7] Skinnider MA (2020). Comprehensive prediction of secondary metabolite structure and biological activity from microbial genome sequences. Nat. Commun..

[8] Cimermancic P (2014). Insights into secondary metabolism from a global analysis of prokaryotic biosynthetic gene clusters. Cell.

[9] Hoskisson PA, Seipke RF (2020). Cryptic or silent? the known unknowns, unknown knowns, and unknown unknowns of secondary metabolism. mBio.

[10] Schwarz J, Hubmann G, Rosenthal K, Lütz S (2021). Triaging of culture conditions for enhanced secondary metabolite diversity from different bacteria. Biomolecules.

[11] Liu Z, Zhao Y, Huang C, Luo Y (2021). Recent advances in silent gene cluster activation in Streptomyces. Front. Bioeng. Biotechnol..

[12] Ossai J, Khatabi B, Nybo SE, Kharel MK (2022). Renewed interests in the discovery of bioactive actinomycete metabolites driven by emerging technologies. J. Appl. Microbiol..

[13] Heng E, Tan LL, Zhang MM, Wong FT (2021). CRISPR-Cas strategies for natural product discovery and engineering in actinomycetes. Process Biochem..

[14] Libis V (2022). Multiplexed mobilization and expression of biosynthetic gene clusters. Nat. Commun..

[15] Harvey CJB (2018). HEx: A heterologous expression platform for the discovery of fungal natural products. Sci. Adv..

[16] Gao C, Hindra, Mulder D, Yin C, Elliot MA (2012). Crp is a global regulator of antibiotic production in *Streptomyces*. mBio.

[17] Wang W (2020). Harnessing the intracellular triacylglycerols for titer improvement of polyketides in Streptomyces. Nat. Biotechnol..

[18] Mingyar E (2021). A regulator based “semi-targeted” approach to activate silent biosynthetic gene clusters. Int. J. Mol. Sci..

[19] Krause J, Handayani I, Blin K, Kulik A, Mast Y (2020). Disclosing the potential of the SARP-type regulator PapR2 for the activation of antibiotic gene clusters in Streptomycetes. Front. Microbiol..

[20] Ou X (2008). SarA influences the sporulation and secondary metabolism in Streptomyces coelicolor M145. Acta Biochim. Biophys. Sin..

[21] Lee H-N, Kim J-S, Kim P, Lee H-S, Kim E-S (2013). Repression of antibiotic downregulator WblA by AdpA in Streptomyces coelicolor. Appl. Environ. Microbiol..

[22] Romano S, Jackson SA, Patry S, Dobson ADW (2018). Extending the “One Strain Many Compounds” (OSMAC) principle to marine microorganisms. Mar. Drugs.

[23] Liu M (2017). A systems approach using OSMAC, Log P and NMR fingerprinting: An approach to novelty. Synth. Syst. Biotechnol..

[24] Wong, F. T. A general multipronged activation approach for natural product discovery in Actinomycetes 54 actinobacterial strains with genetic and cultivation based activation. *MassIVE*10.25345/C53X83W53 (2023).

[25] Ng SB (2018). The 160K Natural Organism Library, a unique resource for natural products research. Nat. Biotechnol..

[26] Tan, L. L. et al. In *Recombineering: Methods and Protocols* (ed Christopher R. Reisch) 207–225 (Springer US, 2022).

[27] Tong Y (2020). CRISPR–Cas9, CRISPRi and CRISPR-BEST-mediated genetic manipulation in streptomycetes. Nat. Protoc..

[28] Bierman M (1992). Plasmid cloning vectors for the conjugal transfer of DNA from Escherichia coli to Streptomyces spp. Gene.

[29] Snoeck N (2019). Serine integrase recombinational engineering (SIRE): a versatile toolbox for genome editing. Biotechnol. Bioeng..

[30] Groth AC, Fish M, Nusse R, Calos MP (2004). Construction of transgenic Drosophila by using the site-specific integrase from phage ϕC31. Genetics.

[31] Guha TK, Calos MP (2020). Nucleofection of phiC31 integrase protein mediates sequence-specific genomic integration in human cells. J. Mol. Biol..

[32] Rubtsova M (2008). Expression of active Streptomyces phage phiC31 integrase in transgenic wheat plants. Plant Cell Rep..

[33] Wang W (2013). An engineered strong promoter for Streptomycetes. Appl. Environ. Microbiol..

[34] Combes P, Till R, Bee S, Smith MCM (2002). The *Streptomyces* genome contains multiple Pseudo-*attB* sites for the ϕC31-encoded site-specific recombination system. J. Bacteriol..

[35] Zhang Z (2020). Antibiotic production in *Streptomyces* is organized by a division of labor through terminal genomic differentiation. Sci. Adv..

[36] Zhang Z, Shitut S, Claushuis B, Claessen D, Rozen DE (2022). Mutational meltdown of putative microbial altruists in Streptomyces coelicolor colonies. Nat. Commun..

[37] Qi Y, Nepal KK, Blodgett JAV (2021). A comparative metabologenomic approach reveals mechanistic insights into *Streptomyces* antibiotic crypticity. Proc. Natl Acad. Soc..

[38] Wang M (2016). Sharing and community curation of mass spectrometry data with Global Natural Products Social Molecular Networking. Nat. Biotechnol..

[39] Xu Z, Li Y, Wang Y, Deng Z, Tao M (2019). Genome-wide mutagenesis links multiple metabolic pathways with actinorhodin production in Streptomyces coelicolor. Appl. Environ. Microbiol..

[40] Furumai T (2002). TPU-0037-A, B, C and D, novel lydicamycin congeners with anti-MRSA activity from Streptomyces platensis TP-A0598. J. Antibiot..

[41] Wong FT (2023). Enhancing armeniaspirols production through multi-level engineering of a native Streptomyces producer. Authorea.

[42] Ertl P, Schuhmann T (2019). A systematic cheminformatics analysis of functional groups occurring in natural products. J. Nat. Prod..

[43] Whitt J, Shipley SM, Newman DJ, Zuck KM (2014). Tetramic acid analogues produced by coculture of Saccharopolyspora erythraea with Fusarium *pallidoroseum*. J. Nat. Prod..

[44] Osterhage C, Kaminsky R, König GM, Wright AD, Ascosalipyrrolidinone A (2000). an antimicrobial alkaloid, from the obligate marine fungus Ascochyta salicorniae. J. Org. Chem..

[45] Toda S (1993). A new neuritogenetic compound BU-4514N produced by Microtetraspora sp. J. Antibiot..

[46] Wang G (2019). CRAGE enables rapid activation of biosynthetic gene clusters in undomesticated bacteria. Nat. Microbiol..

[47] Ayikpoe RS (2022). A scalable platform to discover antimicrobials of ribosomal origin. Nat. Commun..

[48] Grkovic T (2020). National Cancer Institute (NCI) program for natural products discovery: rapid isolation and identification of biologically active natural products from the NCI prefractionated library. ACS Chem. Biol..

[49] Libis V (2019). Uncovering the biosynthetic potential of rare metagenomic DNA using co-occurrence network analysis of targeted sequences. Nat. Commun..

[50] Liang M (2022). Activating cryptic biosynthetic gene cluster through a CRISPR–Cas12a-mediated direct cloning approach. Nucleic Acids Res..

[51] Zhang MM (2017). CRISPR–Cas9 strategy for activation of silent Streptomyces biosynthetic gene clusters. Nat. Chem. Biol..

[52] Chambers MC (2012). A cross-platform toolkit for mass spectrometry and proteomics. Nat. Biotechnol..

[53] Fukuda T (2015). Tolypoalbin, a new tetramic acid from Tolypocladium album TAMA 479. J. Antibiot..

[54] Herath, K. et al. Isolation, structure elucidation, and antibacterial activity of methiosetin, a tetramic acid from a tropical sooty mold (*Capnodium.* sp.). *J. Nat. Prod.* **75**, 420–424 (2012). 10.1021/np200857y 22288374

[55] Ondeyka JG (2014). Isolation, structure elucidation and antibacterial activity of a new tetramic acid, ascosetin. J. Antibiot..

[56] Wright AD, Osterhage C, König GM (2003). Epicoccamide, a novel secondary metabolite from a jellyfish-derived culture of Epicoccum purpurascens. Org. Biomol. Chem..

[57] Singh SB (2002). Structure, stereochemistry, and biological activity of integramycin, a novel hexacyclic natural product produced by Actinoplanes sp. that Inhibits HIV-1 Integrase. Org. Lett..

[58] Jadulco RC (2014). Isolation of pyrrolocins A–C: cis- and trans-decalin tetramic acid antibiotics from an endophytic fungal-derived pathway. J. Nat. Prod..

[59] Watanabe T (2012). Isolation and characterization of Signermycin B, an antibiotic that targets the dimerization domain of histidine kinase walK. Antimicrob. Agents Chemother..

[60] Boros C, Dix A, Katz B, Vasina Y, Pearce C (2003). Isolation and identification of cissetin-a setin-like antibiotic with a novel cis-octalin ring fusion. J. Antibiot..

[61] Inokoshi J (2013). Epi-trichosetin, a new undecaprenyl pyrophosphate synthase inhibitor, produced by Fusarium oxysporum FKI-4553. J. Antibiot..

[62] Segeth MP (2003). Coniosetin, a novel tetramic acid antibiotic from Coniochaeta ellipsoidea DSM 13856. J. Antibiot..

[63] Hellwig V (2002). Altersetin, a new antibiotic from cultures of endophytic Alternaria spp. Taxonomy, fermentation, isolation, structure elucidation and biological activities. J. Antibiot..

[64] Singh SB (1998). Equisetin and a novel opposite stereochemical homolog phomasetin, two fungal metabolites as inhibitors of HIV-1 integrase. Tetrahedron Lett..

[65] Li, H. Aligning sequence reads, clone sequences and assembly contigs with BWA-MEM. Preprint at https://arxiv.org/abs/1303.3997 (2013).

[66] Li H (2009). The sequence alignment/map format and SAMtools. Bioinformatics.

[67] Thorvaldsdóttir H, Robinson JT, Mesirov JP (2012). Integrative Genomics Viewer (IGV): high-performance genomics data visualization and exploration brief. Brief. Informatics.

[68] Tay, D., Wong, F. T. & Lim, Y. H. Supplementary Files. *figshare*10.6084/m9.figshare.24637302.v1 (2023).

[69] Blodgett JAV (2007). Unusual transformations in the biosynthesis of the antibiotic phosphinothricin tripeptide. Nat. Chem. Biol..

[70] Blodgett JAV, Zhang JK, Metcalf WW (2005). Molecular cloning, sequence analysis, and heterologous expression of the phosphinothricin tripeptide biosynthetic gene cluster from *Streptomyces viridochromogenes* DSM 40736. Antimicrob. Agents Chemother..

[71] Katoh K, Misawa K, Kuma KI, Miyata T (2002). MAFFT: a novel method for rapid multiple sequence alignment based on fast Fourier transform. Nucleic Acids Res..

[72] Nguyen L-T, Schmidt HA, von Haeseler A, Minh BQ (2014). IQ-TREE: a fast and effective stochastic algorithm for estimating maximum-likelihood phylogenies. Mol. Biol. Evol..

[73] Letunic I, Bork P (2021). Interactive Tree Of Life (iTOL) v5: an online tool for phylogenetic tree display and annotation. Nucleic Acids Res..

