# Supplementary material for: Exploring a general multi-pronged activation strategy for natural product discovery in Actinomycetes
Source: Commun Biol. 2024 Jan 6;7:50. doi: 10.1038/s42003-023-05648-7 (PMC10771470; doi:10.1038/s42003-023-05648-7)
Supplement: Supplementary file 1 — Supplemental material [file 42003_2023_5648_MOESM1_ESM.pdf]

Supporting Information

Exploring a general multi-pronged activation strategy for natural product discovery in Actinomycetes.

Dillon W. P. Tay[a], Lee Ling Tan[b], Elena Heng[b], Nadiah Zulkarnain[b], Kuan Chieh Ching[c], Mario Wibowo[c], Elaine Jinfeng Chin[c], Zann Y. Tan[c], Chung Yan Leong[c], Veronica Wee Pin Ng[c], Lay Kien Yang[c], Deborah Chwee San Seow[c], Yi Wee Lim[a], Winston Koh[d], Lokanand Koduru[b], Yoganathan Kanagasundaram[c], Siew Bee Ng[c], Yee Hwee Lim[a,e#], Fong Tian Wong[a,b#]

Affiliations:

[a] Institute of Sustainability for Chemicals, Energy and Environment (ISCE[2]), Agency for Science, Technology and Research (A*STAR), 8 Biomedical Grove, #07-01 Neuros Building, Singapore 138665, Republic of Singapore.

[b] Molecular Engineering Lab, Institute of Molecular and Cell Biology (IMCB), Agency for Science, Technology and Research (A*STAR), 61 Biopolis Drive, #07-06, Proteos, Singapore 138673, Republic of Singapore.

[c] Singapore Institute of Food and Biotechnology Innovation (SIFBI), Agency for Science, Technology and Research (A*STAR), 31 Biopolis Way, #01-02, Nanos, Singapore 138669, Republic of Singapore.

[d] Bioinformatics Institute (BII), Agency of Science, Technology and Research (A*STAR), 30 Biopolis Street, #07-01, Matrix, Singapore 138671, Republic of Singapore

[e] Synthetic Biology Translational Research Program, Yong Loo Lin School of Medicine, National University of Singapore, 10 Medical Drive, Singapore 117597, Republic of Singapore

#Corresponding authors

Y.H.L.: lim_yee_hwee@isce2.a-star.edu.sg

F.T.W.: wongft@imcb.a-star.edu.sg

List of content:

Figure S1. Heatmap of metabolite upregulation by phiC31 integration of RedD and FAS in A1123 across 5 media.
Figure S2. Comparison of Surfactin A, B, and C LC-MS/MS abundances in fermentation extracts from 5 RedD mutants of A1123 in CA07LB media.
Figure S3. Genomic analyses of A1123 mutants integrated with npC697.
Table S7. Media compositions
Table S8. Identified metabolites detected by LC-MS/MS across 54 strains and their 459 mutants.
Figure S4. Fold change in metabolite LC-MS/MS abundances detected due to integration of SarA regulator across 5 strains (A1123, A1137, A2056, A33995, A80510).
Figure S5. Bioactivity profiling heatmap of strains with SarA regulator integrated.
Figure S6. Bioactivity profiling heatmap of strains with Crp regulator integrated.
Figure S7. Tandem mass spectra (MS/MS) comparison of lydicamycin analogs TPU-0037-A and TPU-0037-C.
Figure S8. Tandem mass spectra (MS/MS) comparison of lydicamycin analogs TPU-0037-A and TPU-0037-D.
Figure S9. Lydicamycin pathway specific regulator: lydicamycin cluster in A80510**.** A80510 LuxR amino acid sequence.
Figure S10. Bioactivity profiling heatmap of strains with AdpA regulator integrated.
Figure S11 Bioactivity profiling heatmap of strains with RedD regulator integrated.
Figure S12. Upregulation of tetramic acid compounds BE-54476-A (**1**) and BE-54476-B (**2**) in A58051 and its edited strains.
Supplementary Material for BE-54476-A and BE-54476-B. Bioactivity of Compounds **1** and **2**
Figure S13. Structures of tetramic acid analogs and similar compounds.
Table S9. NMR assignments of BE-54476-A (**1**) and B (**2**).
Figure S14. Chemical structures of tetramic acid analogs.
Figure S15. Integration of overexpression cassette for SCO6196 into *Streptomyces sp*. A58051.
Figure S16. ^{1}H NMR spectrum (MeOH-d_4, 400 MHz) of tetramic acid **1**.
Figure S17. ^{13}C NMR spectrum (MeOH-d_4, 100 MHz) of tetramic acid **1**.
Figure S18. HSQC spectrum of tetramic acid **1**.
Figure S19. HMBC spectrum of tetramic acid **1**.
Figure S20. COSY spectrum of tetramic acid **1**.
Figure S21. NOESY spectrum of tetramic acid **1**.
Figure S22. ^{1}H NMR spectrum (MeOH-d_4, 400 MHz) of tetramic acid **2**.
Figure S23. ^{13}C NMR spectrum (MeOH-d_4, 100 MHz) of tetramic acid **2**.
Figure S24. HSQC spectrum of tetramic acid **2**.
Figure S25. HMBC spectrum of tetramic acid **2**.
Figure S26. COSY spectrum of tetramic acid **2**.
Figure S27. NOESY spectrum of tetramic acid **2**.
Figure S28. (–)-HRESIMS spectra of compounds **1** and **2**.
Figure S29. UV spectra of compounds **1** and **2**.
Figure S30. Dose response curve against *Klebsiella aerogenes* (ATCC® 13048™) (KA13048), *Pseudomonas aeruginosa* (ATCC® 9027™) (PA9027), *Aspergillus fumigatus* (ATCC® 46645™) (AF46645).
Figure S31. Dose response curve against *Staphylococcus aureus* Rosenbach (ATCC® 25923™) (SA25923).
Figure S32. Dose response curve against *Acinetobacter baumannii* (ATCC® 19606™) (ACB19606),
Figure S33. Dose response curve against the human lung carcinoma cells A549 (ATCC® CCL-185™) (A549).
Table S10. Bioactivity characterization of compounds BE-54476-A (**1**) and BE-54476-B (**2**).
Figure S34. ^{1}H NMR spectrum of [Leu^{7}] surfactin iso-C14.
Figure S35. ^{13}C NMR spectrum of [Leu^{7}] surfactin iso-C14.
Figure S36. ^{1}H NMR spectrum of [Leu^{7}] surfactin anteiso-C15.
Figure S37. ^{13}C NMR spectrum of [Leu^{7}] surfactin anteiso-C15.
Figure S38. ^{1}H NMR spectrum of [Leu^{7}] surfactin iso-C15.
Figure S39. ^{13}C NMR spectrum of [Leu^{7}] surfactin iso-C15.
Table S11. Integration cassette sequences.
Figure S40. Representative screening of A1301 mutants integrated with *kasO**p-*SCO6196*.

Figure S41. Representative sanger sequencing of PCR fragment to verify presence of *kasO**p and expression cassette SCO6196 (FAS).
Figure S42. Plasmid map of pCRP63.
Figure S43. Plasmid map of pCRP65.
Figure S44. Plasmid map of pCRP67.
Figure S45. Plasmid map of pCRP178.
Figure S46. Plasmid map of npC697.

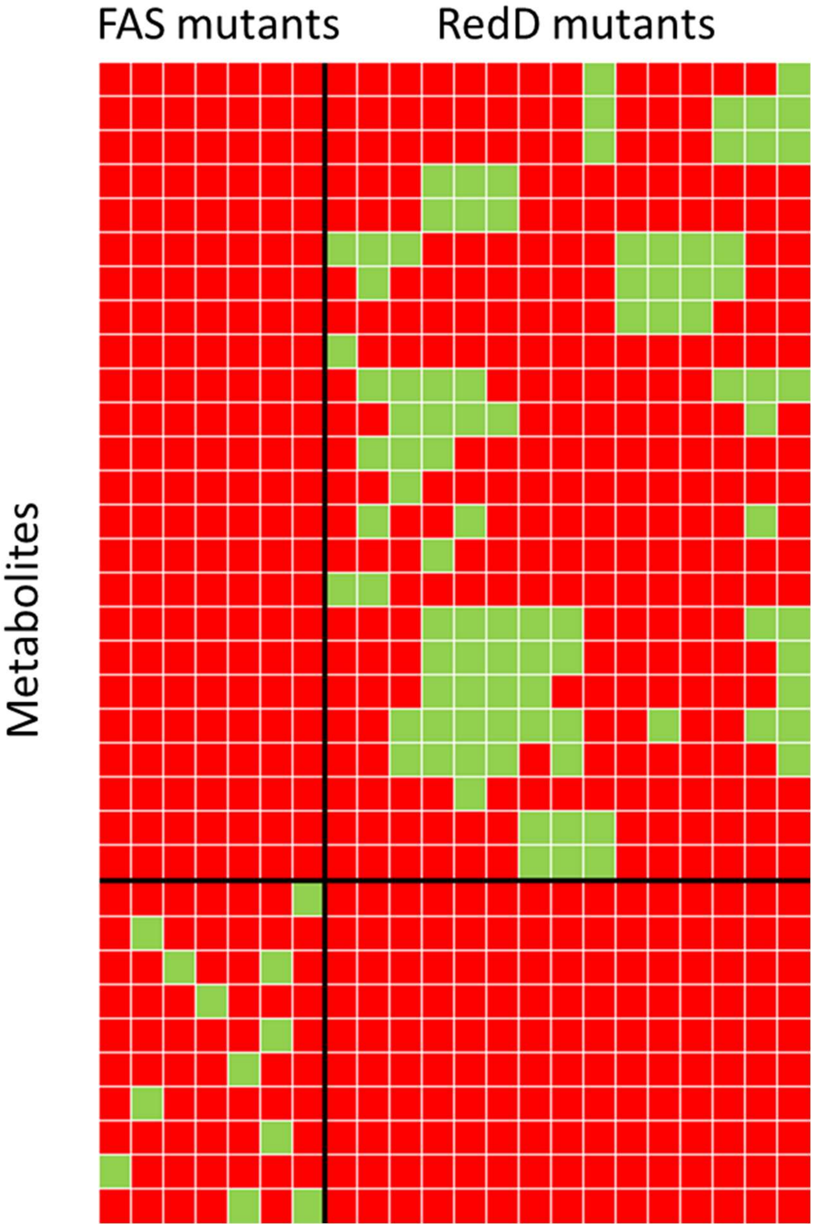

Figure S1. Heatmap of metabolite upregulation by phiC31 integration of RedD (n=15 independent mutants) and FAS (n=7 independent mutants) in A1123 across 5 media (CA02LB, CA07LB, CA08LB, CA09LB, CA10LB). Only selected metabolites not observed in parent strain are shown. Green indicates metabolite observed by LC-MS/MS in the fermentation extract of that mutant, red indicates not observed. Metabolites annotated via the Global Natural Products Social Molecular Networking (GNPS) Molecular Networking workflow (see Methods).

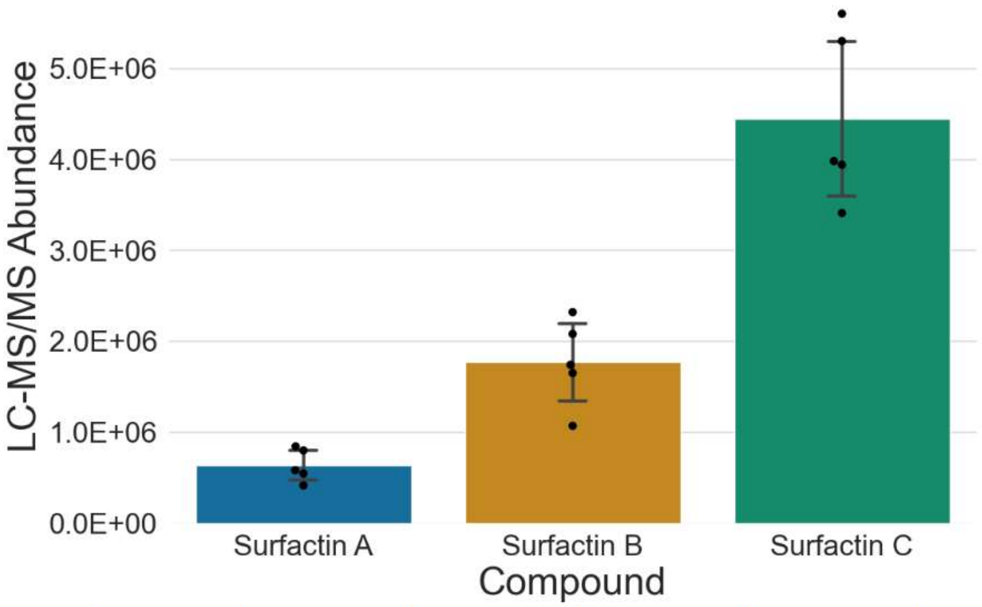

Compound	m/z	A100208	A100209	A100212	A100213	A100214
Surfactin A	1008.66	5.46E+05	5.82E+05	8.44E+05	4.15E+05	8.00E+05
Surfactin B	1022.67	1.65E+06	1.74E+06	2.32E+06	1.07E+06	2.08E+06
Surfactin C	1036.69	3.98E+06	3.94E+06	5.30E+06	3.41E+06	5.60E+06

Figure S2. Comparison of Surfactin A, B, and C LC-MS/MS abundances in fermentation extracts from n=5 independent RedD mutants (A100208, A100209, A100212, A100213 and A100214) of A1123 in CA07LB media. See Methods for details on LC-MS/MS analysis of fermentation extracts. Error bars are shown for 1 standard deviation.

.

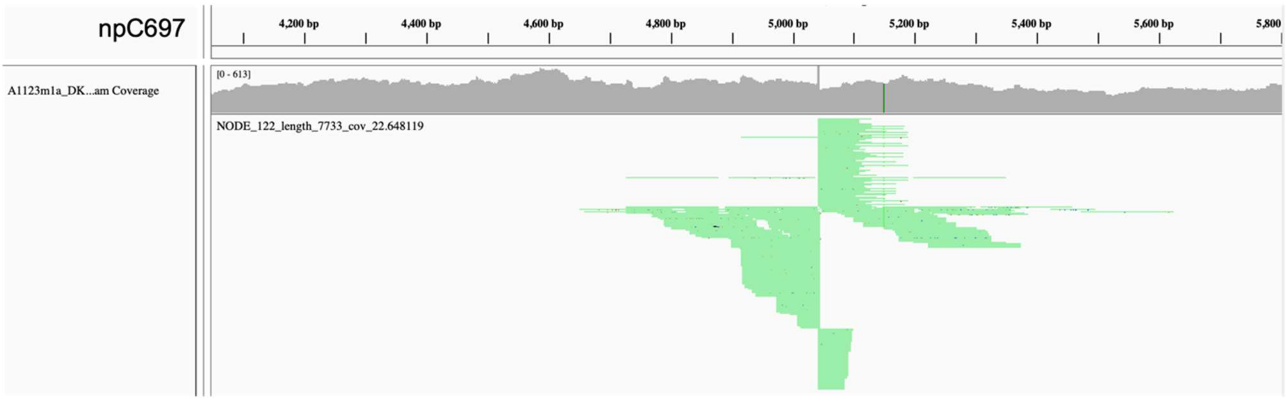

Strain	Native Strain ID	Modification	X3	X7	counts
A100200	A1123	RedD integration	npC697	NODE_122_length_7733_cov_22.648119	81
A100201	A1123	RedD integration	npC697	NODE_122_length_7733_cov_22.648119	166
A100203	A1123	RedD integration	npC697	NODE_122_length_7733_cov_22.648119	152
A100204	A1123	RedD integration	npC697	NODE_122_length_7733_cov_22.648119	130
A100205	A1123	RedD integration	npC697	NODE_122_length_7733_cov_22.648119	157
A100206	A1123	RedD integration	npC697	NODE_122_length_7733_cov_22.648119	142
A100207	A1123	RedD integration	npC697	NODE_122_length_7733_cov_22.648119	163
A100208	A1123	RedD integration	npC697	NODE_122_length_7733_cov_22.648119	90
A100209	A1123	RedD integration	npC697	NODE_122_length_7733_cov_22.648119	103
A100210	A1123	RedD integration	npC697	NODE_122_length_7733_cov_22.648119	136
A100212	A1123	RedD integration	npC697	NODE_122_length_7733_cov_22.648119	170
A100214	A1123	RedD integration	npC697	NODE_122_length_7733_cov_22.648119	259

Figure S3. Genomic analyses of A1123 mutants integrated with npC697. Top: Representative alignment of breakpoint reads crossing from npC697 (RedD integration) into the genome. Numbering of npC697 is given in Figure S46. **Bottom:** Table of number of paired reads (Counts) crossing the break points starting from attP site on npC697 (X3) to other location on the genome (X7). Only one integration event was observed in these strains.

Table S7. Media compositions

CA02LB	CA07LB	CA08LB	CA09LB	CA10LB
Mannitol 20g/L	Glycerol 15g/L	Glucose 15g/L	Beef Extract 10g/L	Soluble Starch 20g/L
Soybean Meal 20g/L	Oatmeal 30g/L	Cane Molasses 20g/L	Yeast Extract 4g/L	Soybean Meal 15g/L
pH 7.5	Yeast Extract 5g/L	Soluble Starch 40g/L	Glucose 20g/L	KH_2PO_4 3g/L
	KH_2PO_4 5g/L	$CaCO_3$ 8g/L	Glycerol 3g/L	Na_2HPO_4.12H2O 2g/L
	Na_2HPO_4.12H2O 5g/L	Cotton seed flour 25g/L	pH 7.0	Mg/LSO_4.7H2O 0.5g/L
	$MgCl_2$.6H2O 1g/L	pH 7.2		*Trace Salts Solution 1ml
	pH natural			pH 7.2

*Trace Salts Solution: Prepare a solution containing 0.2% each of the following: $FeSO_4.7H_2O$, $MnCl_2.4H_2O$, $ZnSO_4.7H_2O$, $CuSO_4.5H_2O$, $CoCl_2.2H_2O$

Strain ID	1 Desferrioxamine E	2 valinomycin	3 Monactin	4 Surfactin A	5 arthrobactin	6 Monoelaidin	7 Surugamide A
A1090							
A1123				New			
A11345							
A1137							
A1301							
A1532							
A1636							
A2056				Both			
A2278							
A2705							
A2957		Both	Both				
A30639	New						
A33995		Both					
A34001	New	Both					
A34053		Native					
A40707			Both				
A40926							
A4217	New						
A44034	New						
A5252	Both						
A53961							
A58051							
A5858							
A61715		Both					
A6562							
A80510				Native			
A8274							
A8567							
T10	New						
T108							
T118	Both						Both
T1195	Native						
T12							
T1236					Native		
T1312	New						
T1415	New						
T1416							
T1425	New		Both				
T1628	Both						Both
T168	Both						Both
T175	Both						Both
T265					New	New	
T271							
T298				New	Both	New	
T302							
T343	New						
T354	New						Both
T36	Both						Both
T39	Both						Both
T467	New						
T4680		New					
T676							
A100005							
A100006							

Legend

New – new to strain due to mutation
Both – in both native and mutant strain
Native – only in native strain, not observed in mutant strain

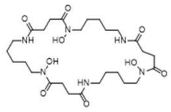

1 Desferrioxamine E

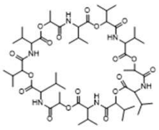

2 Valinomycin

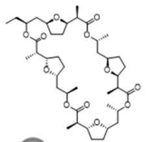

3 Monactin

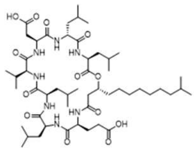

4 Surfactin A

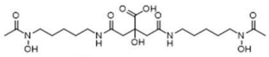

5 Arthrobactin

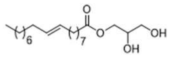

6 Monoelaidin

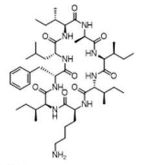

7 Surugamide A

Table S8. Metabolites detected by LC-MS/MS and identified by Global Natural Products Social Molecular Networking (GNPS) across 54 strains and their 459 mutants (see Figure 2). Pink (new) indicates metabolite is only detected in the strain after mutation. Grey (both) indicates that the metabolite is detected in both the native strain and its mutant. Blue (native) indicates that the metabolite is only detected in the native strain but not in its mutants. For analysis details please see Methods.

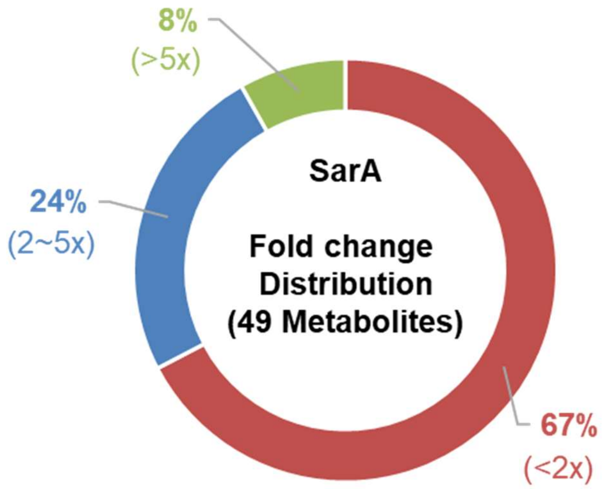

List of Metabolites with >2x fold change

Strain	Metabolite m/z	Fold Change
A2056	261	18.8
A33995	218	10.7
A33995	389	7.1
A80510	871	5.2
A2056	496	4.3
A80510	366	3.9
A2056	510	3.8
A2056	482	3.3
A80510	841	3.2
A80510	853	3.2
A2056	478	2.7
A1137	476	2.6
A2056	451	2.3
A33995	763	2.1
A80510	265	2.1
A33995	366	2.03

Figure S4. Fold change in metabolite LC-MS/MS abundances detected due to integration of SarA regulator across 5 strains (A1123, A1137, A2056, A33995, A80510).

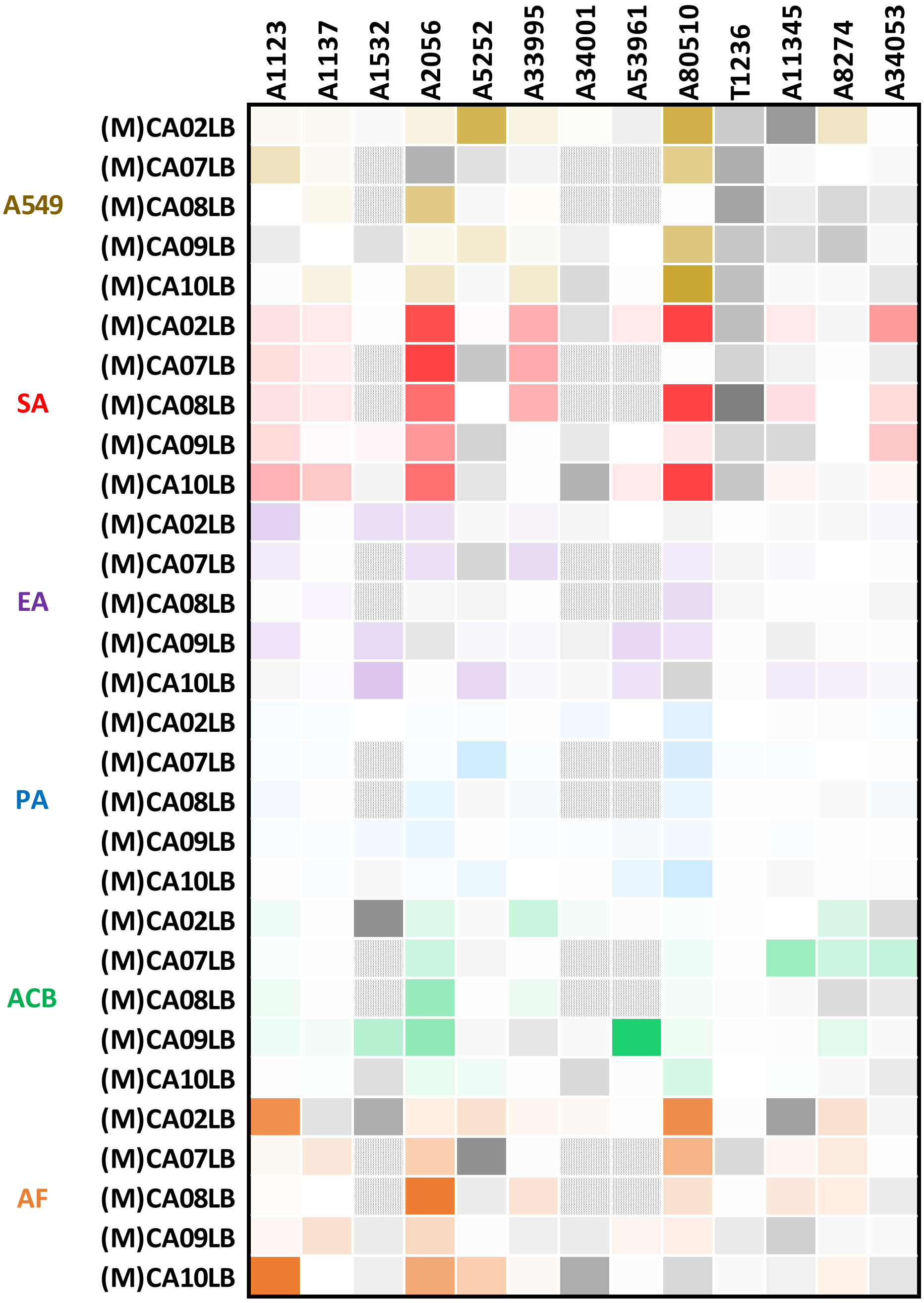

Figure S5. Differential bioactivity profiling of strains with SarA expression cassette integrated compared to their native strains. A549 (brown) = cell cytotoxicity against human lung carcinoma cells. SA (red) = antibacterial activity against *Staphylococcus aureus*. EA (purple) = antibacterial activity against *Klebsiella aerogenes*. PA (blue) = antibacterial activity against *Pseudomonas aeruginosa*. ACB (green) = antibacterial activity against *Acinetobacter baumannii*. AF (orange) = antifungal activity against *Aspergillus fumigatus*.

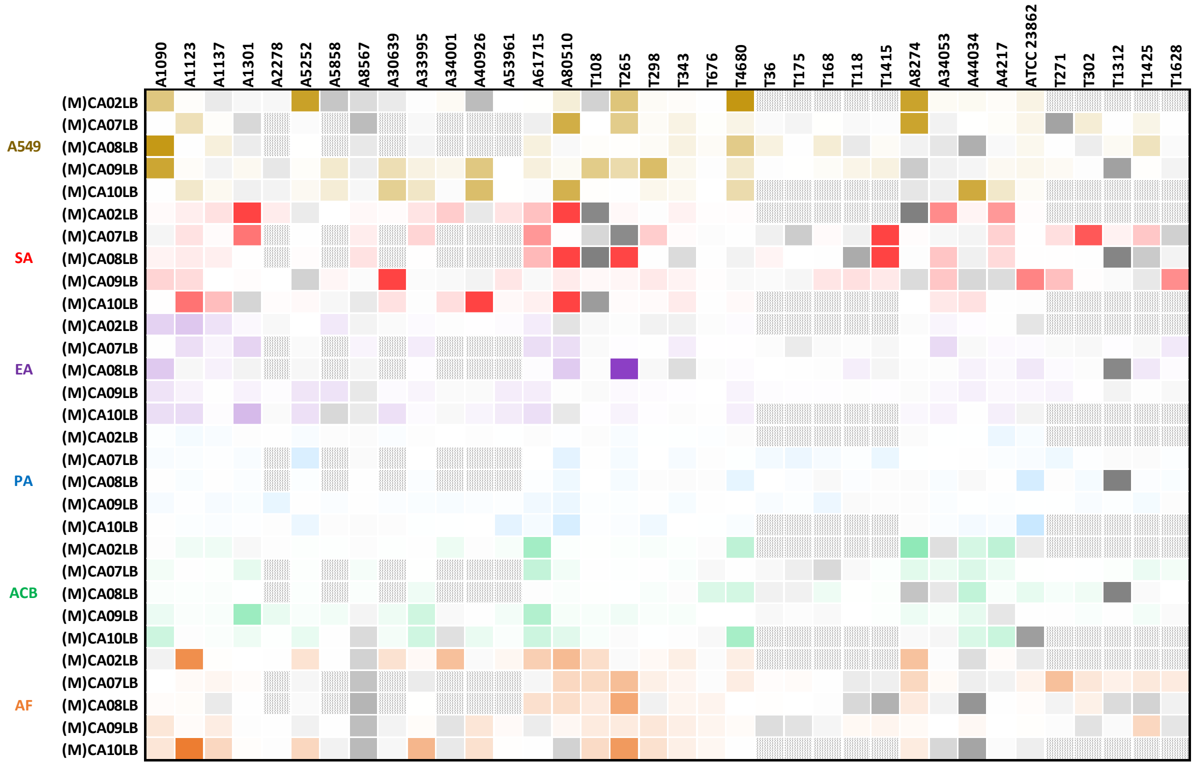

Figure S6. Differential bioactivity profiling of strains with Crp expression cassette integrated compared to their native strains. A549 (brown) = cell cytotoxicity against human lung carcinoma cells. SA (red) = antibacterial activity against *Staphylococcus aureus*. EA (purple) = antibacterial activity against *Klebsiella aerogenes*. PA (blue) = antibacterial activity against *Pseudomonas aeruginosa*. ACB (green) = antibacterial activity against *Acinetobacter baumannii*. AF (orange) = antifungal activity against *Aspergillus fumigatus*.

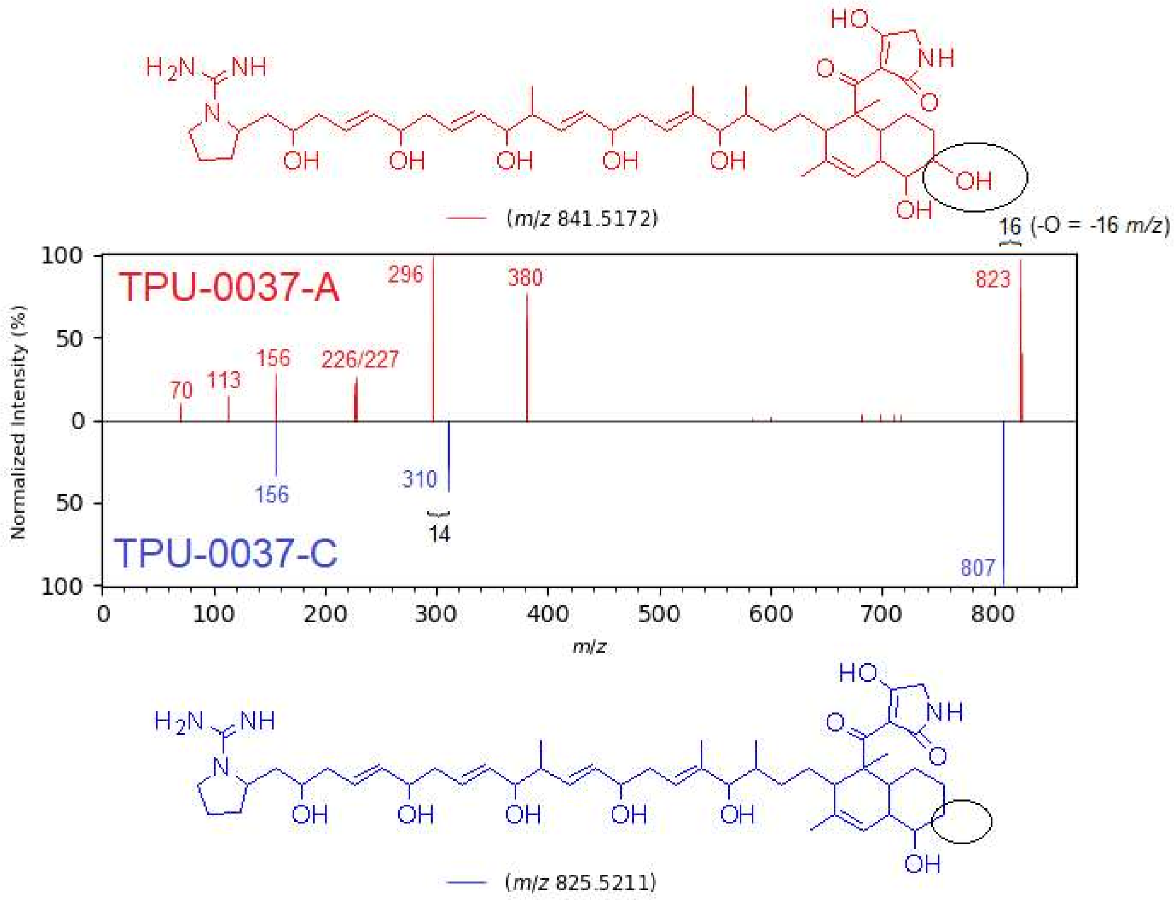

Figure S7. Tandem mass spectra (MS/MS) comparison of lydicamycin analogs TPU-0037-A and TPU-0037-C.

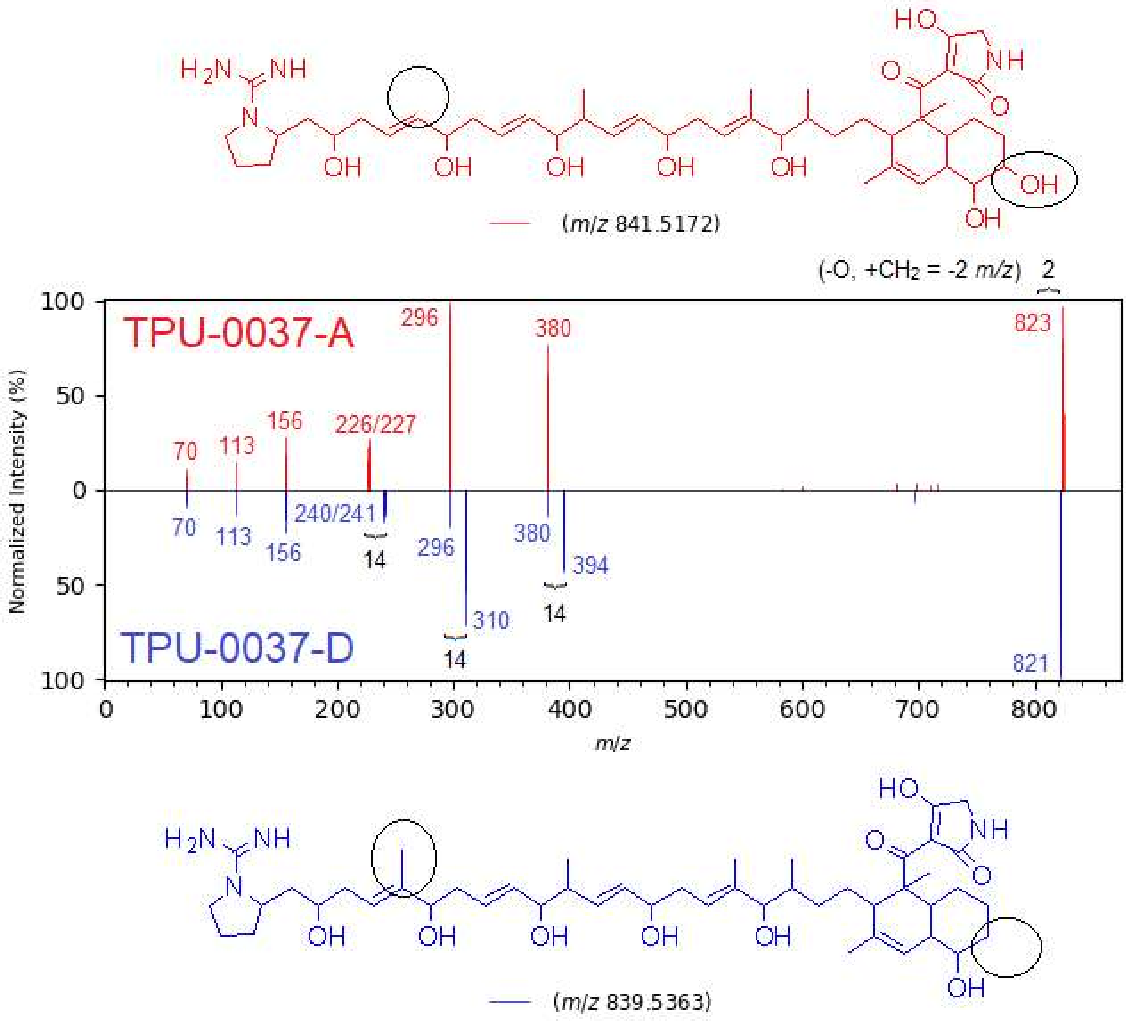

Figure S8. Tandem mass spectra (MS/MS) comparison of lydicamycin analogs TPU-0037-A and TPU-0037-D.

Lydicamycin pathway specific regulator:

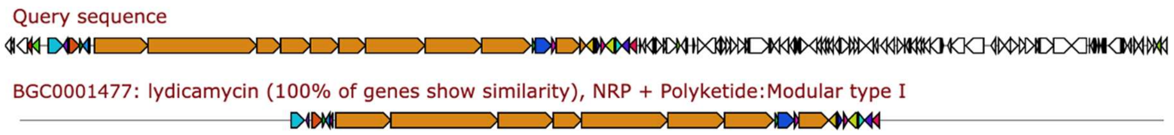

Figure S9. Lydicamycin cluster in A80510 in multi-gene blast against Lydicamycin miBIG, BGC0001477

>LuxR (from A80510, 100% sequence identity to Lydicamycin miBIG, BGC0001477)
MGLVERDAVIAELRTALSDSAKGCGKVAVIRGGIASGKTALFRAFEEHAVASGATLLRASGAPSEQSL
RFGVIEQFFSGATTPPEASAMLSHLTGLEAPQVGGEPPSPVQSGTRDAHDLCVGLLELSRKGPLVVT
VDDHQFADPASLQVLTYLQHRIGTARVMLLLSQGTEPPSDLVAEALRQPYSRQFTLSPLSPEGVGQL
LAQRLDSSAALRQAPGSYALTGGNPLLTRALVDDSLAPGPDASAGAAAGPVTGQAFTRAVLACLHR
GGPQLLRTARAIAVLGEFAAPALLARFLDVRPSVVGGALEALELAGLTIDAQFRYEGTRAAVLDELTPE
ERSALNRRAAELLHHDGVAPSDVVGYLLAAGEADEPWAARVLHAAADQALPHAEQALALGKVAEAV
QYLEFASRSCGDEHKRAMLTARLAWVQWTSSPAAATRHHGPLQTALEKELLSGREVMRLVRSLAW
HGRPKEAVRALESLGAPPEGDGSRDQAERKLTRQWLSRWHPQIFAQVERHAAMSEPPGSPGPARR
PQTAVLPRGTGNSPAVEGAEQVLQRARVRETPLASVISALHELLAAERFERAMYWCNELLQKAEGQ
HAAAWRGVLLDTRAAVSLRLGDLADAERDACSALTALSARSWGVAIGSPLSHAVHAATMRGHFDKA
AEFLNQMIPQAMMDTRYGLQYRTARGHFHLATDRPHAALEDFEAVGDLVVKWKLDHPMTLPWRGD
LAQALVRVGQTDRARELIKDQLRMIGTDSTRMRGVSLGILASVSDLKQRLPLLGEVVDLLQAGGDRYE
LARAFVELGQVWQILGKLDRAQLIRRRALQLAKSCHAEQLYNQLVATREPLNSETTPSQWEDAEGMA
VLSEAERRVAALAALGRTNREIGRKLHITVSTVEQHLTRVYRKLNIKRRADLPVGLPADIADIA

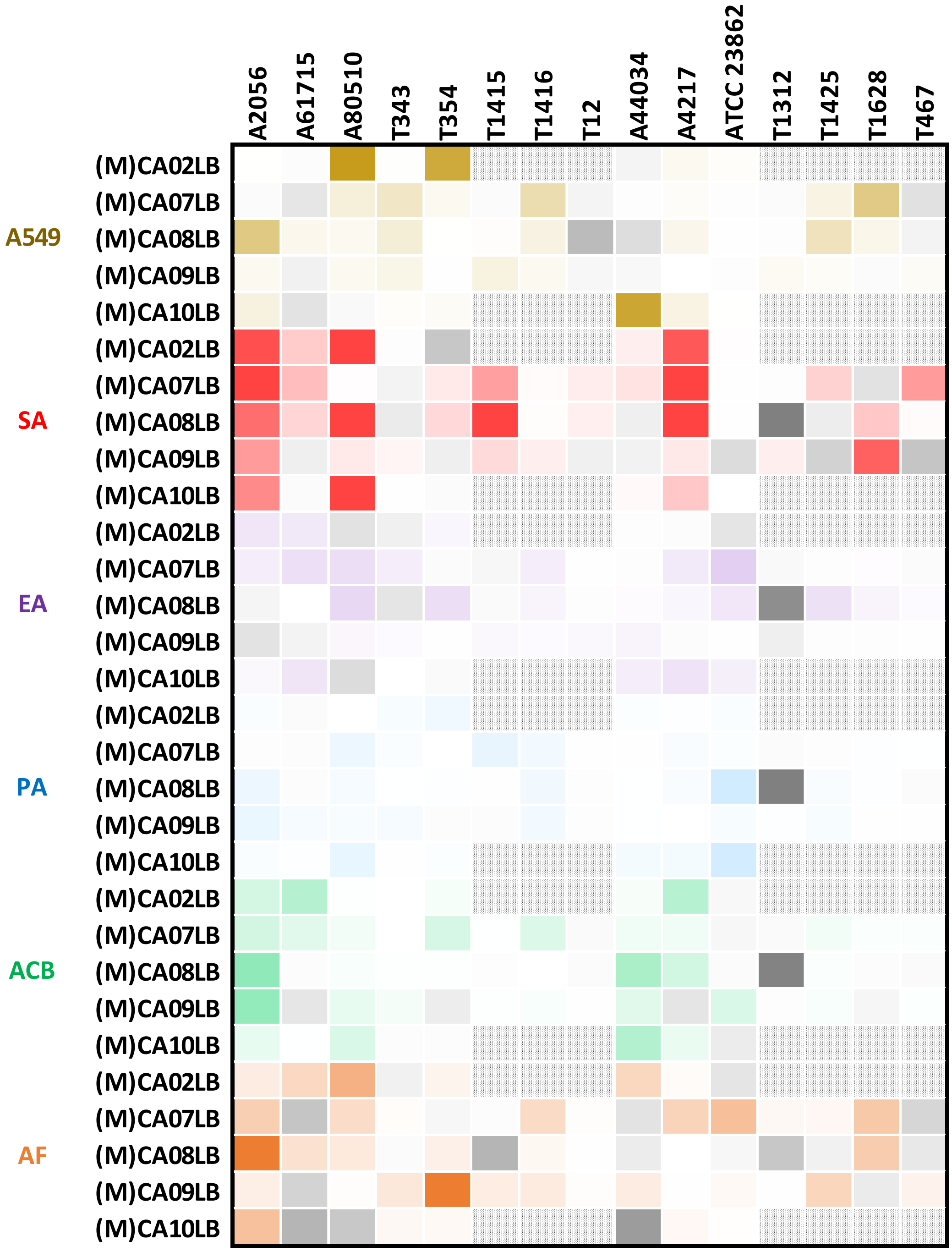

Figure S10. Differential bioactivity profiling of strains with AdpA expression cassette integrated compared to their native strains. A549 (brown) = cell cytotoxicity against human lung carcinoma cells. SA (red) = antibacterial activity against *Staphylococcus aureus*. EA (purple) = antibacterial activity against *Klebsiella aerogenes*. PA (blue) = antibacterial activity against *Pseudomonas aeruginosa*. ACB (green) = antibacterial activity against *Acinetobacter baumannii*. AF (orange) = antifungal activity against *Aspergillus fumigatus*.

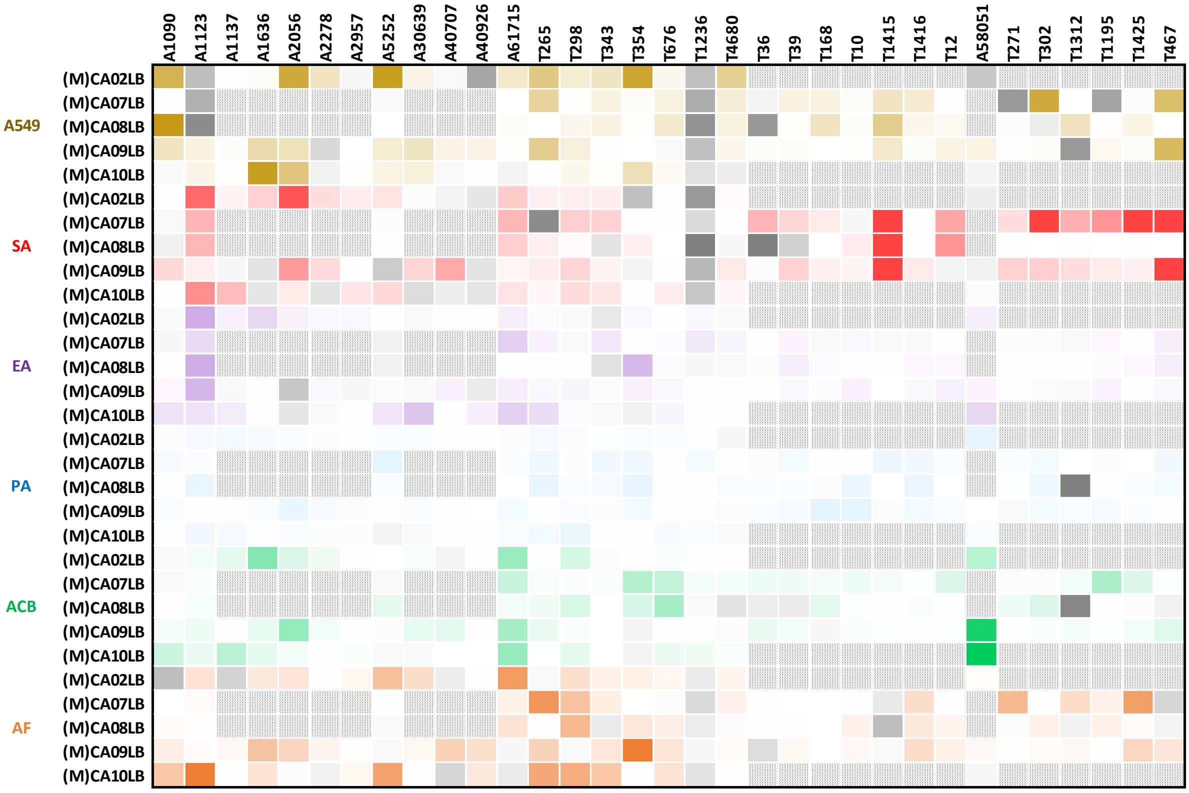

Figure S11. Differential bioactivity profiling of strains with SARP, RedD, expression cassette integrated compared to their native strains. A549 (brown) = cell cytotoxicity against human lung carcinoma cells. SA (red) = antibacterial activity against *Staphylococcus aureus*. EA (purple) = antibacterial activity against *Klebsiella aerogenes*. PA (blue) = antibacterial activity against *Pseudomonas aeruginosa*. ACB (green) = antibacterial activity against *Acinetobacter baumannii*. AF (orange) = antifungal activity against *Aspergillus fumigatus*.

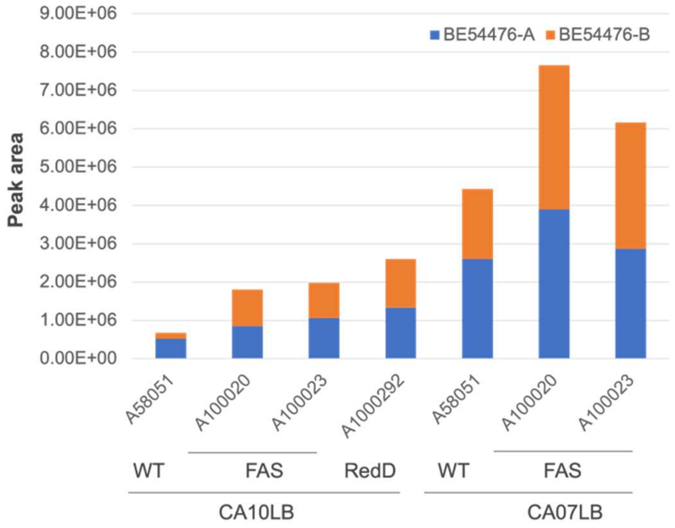

Figure S12. Upregulation of tetramic acid compounds BE-54476-A (**1**) and BE-54476-B (**2**) in A58051 and its edited strains.

Bioactivity of Compounds 1 and 2

Compounds **1** (BE 54476-A) and **2** (BE 54476-B) were tested for their antimicrobial activity against a panel of microorganisms consisting of Gram-positive and Gram-negative bacteria, as well as one fungal strain: *Acinetobacter baumannii* (ATCC® 19606™), *Klebsiella aerogenes* (ATCC® 13048™), *Pseudomonas aeruginosa* (ATCC® 9027™), *Staphylococcus aureus Rosenbach* (ATCC® 25923™) and *Aspergillus fumigatus* (ATCC® 46645™).

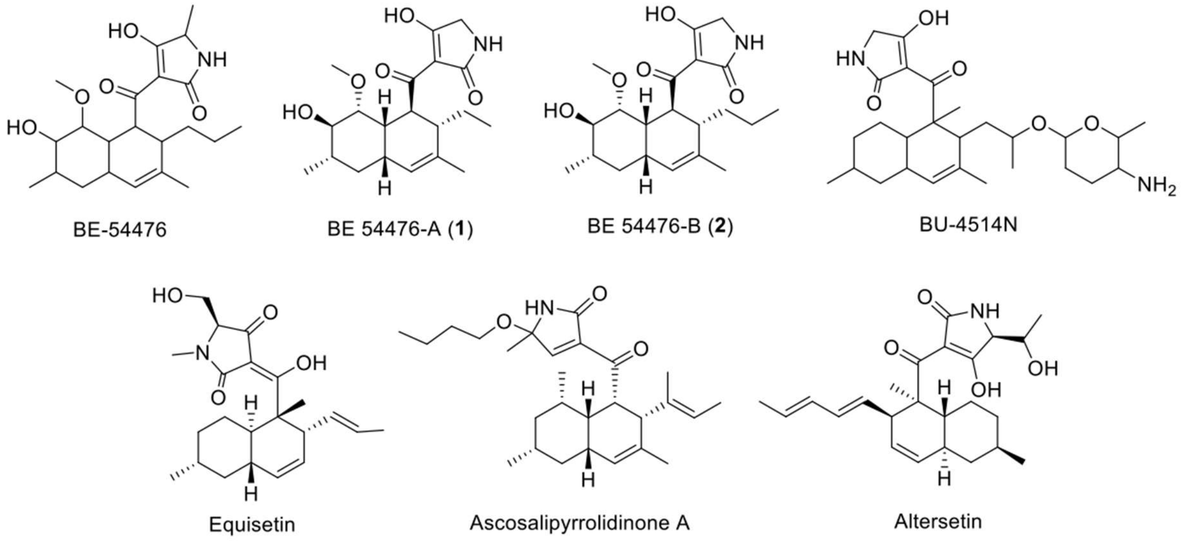

Figure S13. Structures of tetramic acid analogs and similar compounds.

Structurally similar tetramic acid analogs have been reported to exhibit antimicrobial activity, mainly against Gram-positive bacteria or anti-tumor activity. For example, equisetin has been reported to be active against *Staphylococcus erythraea* and *Staphylococcus aureus*.[1] Ascosalipyrrolidone A was reported to be active against *Bacillus megaterium*,[2] *Mycoptypha microsporosum*, and *Microbotyryum violaceum*, while BU-4514N[3] and the fungal metabolite altersetin[4] possess inhibitory activity against several Gram-positive bacteria. Notably, the antibiotic BE-54476, which possesses high structural similarity with compounds **1** and **2** was reported to not only have anti-tumor activity, but also antibacterial activity against Gram-positive bacteria such as *Bacillus subtilis*, *Enterococcus faecalis*, and *Staphylococcus aureus*.[5] Both compounds **1** and **2** were found to be inactive against Gram-negative bacteria *Klebsiella aerogenes* (ATCC® 13048™), *Pseudomonas aeruginosa* (ATCC® 9027™) and fungal strain *Aspergillus fumigatus* (ATCC® 46645™) (**Table S10, Figure S30**). However,

Compounds **1** and **2** showed activity against *Staphylococcus aureus* Rosenbach (ATCC® 25923™) (**Table S10, Figure S31**) with minimum inhibitory concentrations (MIC_{50}) of 14.5 µM and minimum bactericidal concentrations (MBC_{50}) of 65.5 µM for compound **1** and MIC_{50} of 7.5 µM, MBC_{50} of 22.3 µM for Compound **2**. Interestingly, the compounds were also found to be active against Gram-negative strain *Acinetobacter baumannii* (ATCC® 19606™) (**Table S10, Figure S32**), with MIC_{50} of 9.8 µM for Compound **1** and MIC_{50} of 6.9 µM for Compound **2**. No MBC activities were observed in antimicrobial testing of *Acinetobacter baumannii*. Apart from that, both compounds were also tested for their cytotoxicity against the human lung carcinoma cell line A549 (ATCC® CCL-185™) with IC_{50} of 34.4 µM and 46.3 µM respectively (**Table S10, Figure S33**).

Table S9. ^1H and ^{13}C NMR data of tetramic acid analogs, BE-54476-A (**1**) and B (**2**) in MeOH-d_4.

Pos.	**1**		**2**	
	δ_C, type	δ_H, mult. (J = Hz)	δ_C, type	δ_H, mult. (J = Hz)
1	201.8,[a] C	–	[b]	–
2	38.9, CH	4.06, dd (12.0)	39.3, CH	4.09, dd (12.3)
3	45.3, CH	2.57, br d (10.0)	44.9, CH	2.53, br d (10.0)
4	135.4, C	–	135.8, C	–
5	129.0, CH	5.61, d (6.0)	128.4, CH	5.56, d (5.8)
6	37.3, CH	2.12, m	37.2, CH	2.10, m
7	38.2, CH_2	1.15, ddd (12.3, 12.3, 12.3); 1.65, m	38.2, CH_2	1.14, ddd (12.9, 12.9, 12.9); 1.63, m
8	38.7, CH	1.39, m	38.9, CH	1.39, m
9	75.1, CH	3.43, m	75.1, CH	3.44, m
10	88.9, CH	2.99, dd (4.3, 9.8)	88.8, CH	2.97, dd (4.4, 9.7)
11	40.8, CH	2.76, ddd (4.3, 4.3,12.0)	40.9, CH	2.74, dt (4.4, 4.4, 12.3)
12	21.9, CH_2	1.29, m	18.2, CH_2	1.48, m
13	8.6, CH_3	0.75, t (7.4)	32.3, CH_2	1.29, m
14	21.1, CH_3	1.63, s	21.2, CH_3	1.63, s
15	18.8, CH_3	1.04, d (6.4)	18.8, CH_3	1.04, d (6.3)
16	58.3, CH_3	3.26, s	58.3, CH_3	3.25, s
2'	178.2,[a] C	–	178.0, C	–
3'	104.5,[a] C	–	[b]	–
4'	196.1,[a] C	–	196.0, C	–
5'	52.9,[a] CH_2	3.75 (2H), br s	52.5, CH_2	3.73 (2H), br s
13-Me	–	–	15.0, CH_3	0.81, t (6.9)

^1H (400 MHz) and ^{13}C (100 MHz) in MeOH-d_4. Assignments based on COSY, NOESY, HSQC and HMBC. Chemical shifts (δ) in ppm. s: singlet; br s: broad singlet; d: doublet; br d: broad doublet; t: triplet; m: multiplet. One proton unless otherwise stated. [a]Broad signal. [b]Not detected.

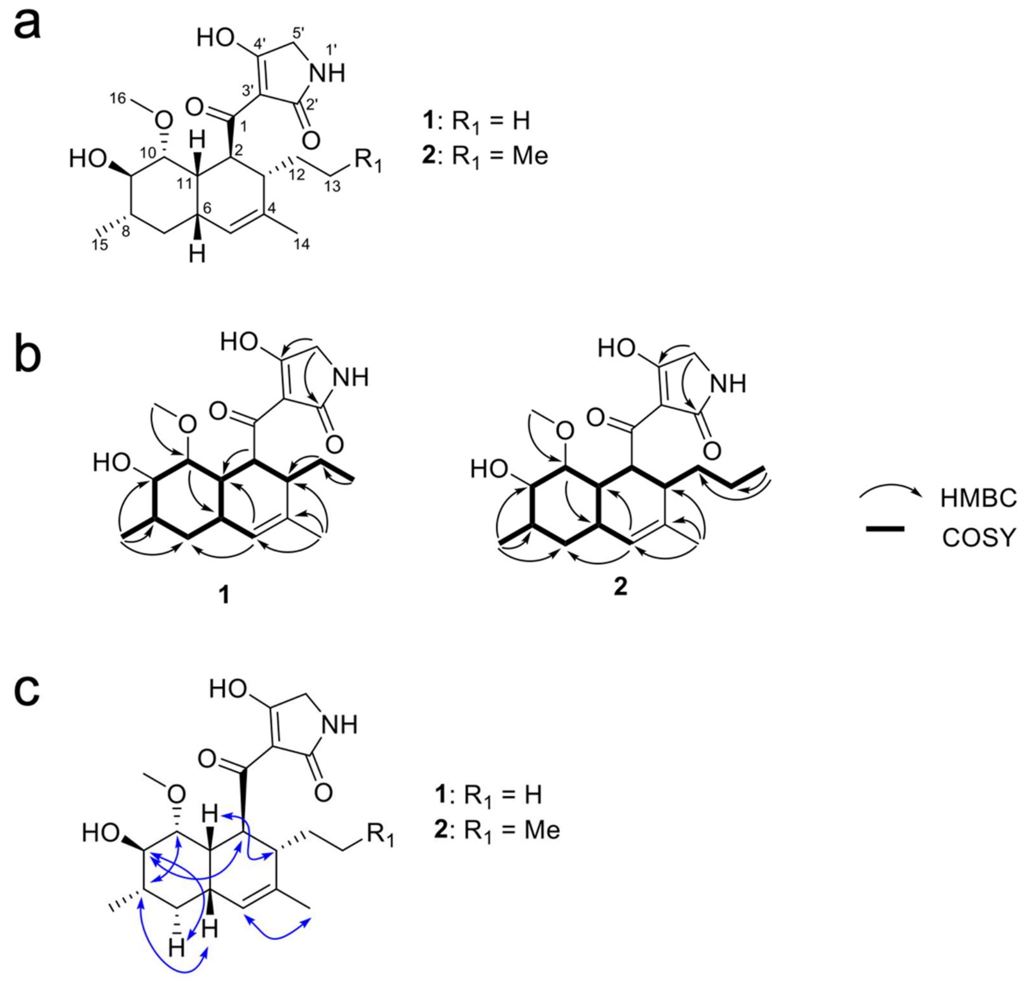

Figure S14: Chemical structures of the tetramic acid analogs, BE 54476-A (**1**) and B (**2**) (relative configurations of the stereocenters were determined via NOESY correlations). (B) Selected COSY and HMBC correlations of BE 54476-A (**1**) and B (**2**). (C) Selected NOESY correlations of BE 54476-A (**1**) and B (**2**).

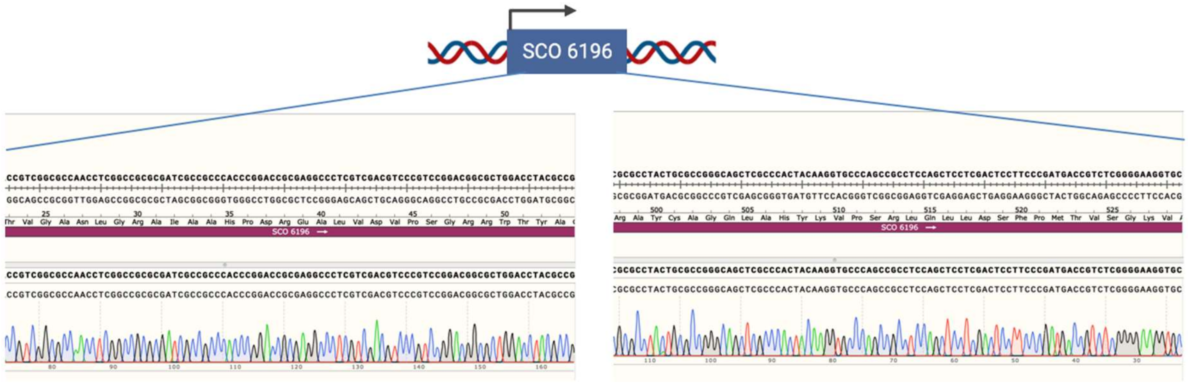

Figure S15. Integration of overexpression cassette for SCO6196 into *Streptomyces sp*. A58051.

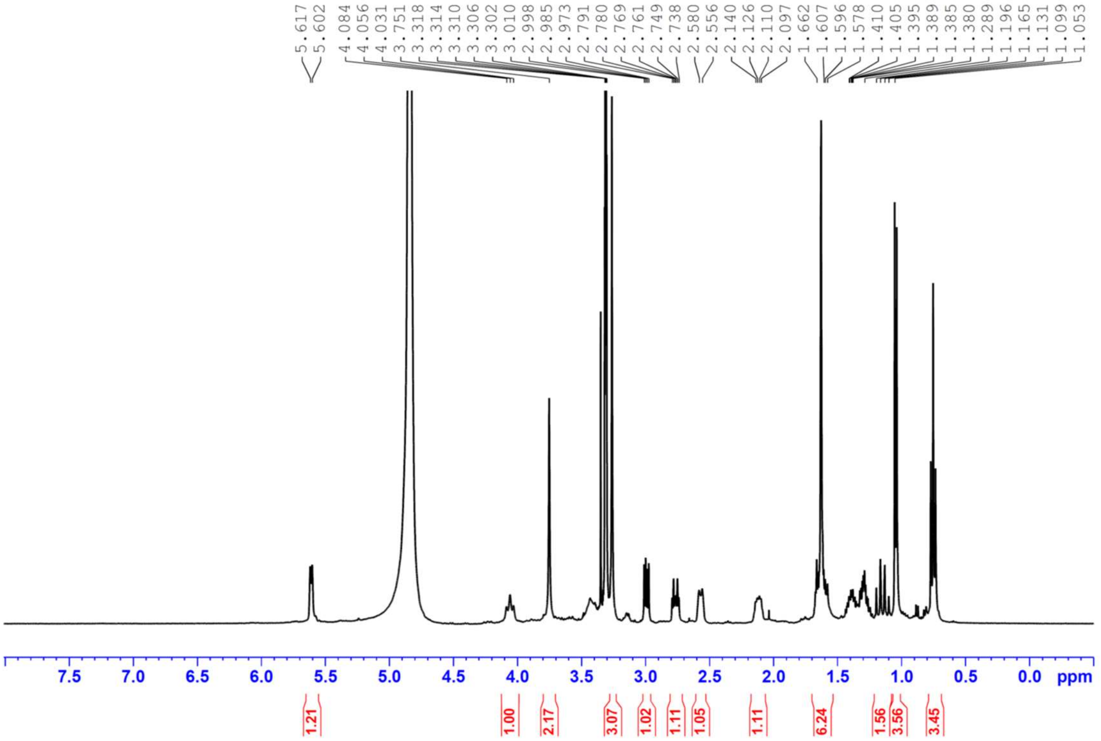

Figure S16. ^{1}H NMR spectrum (MeOH-d_4, 400 MHz) of tetramic acid **1**.

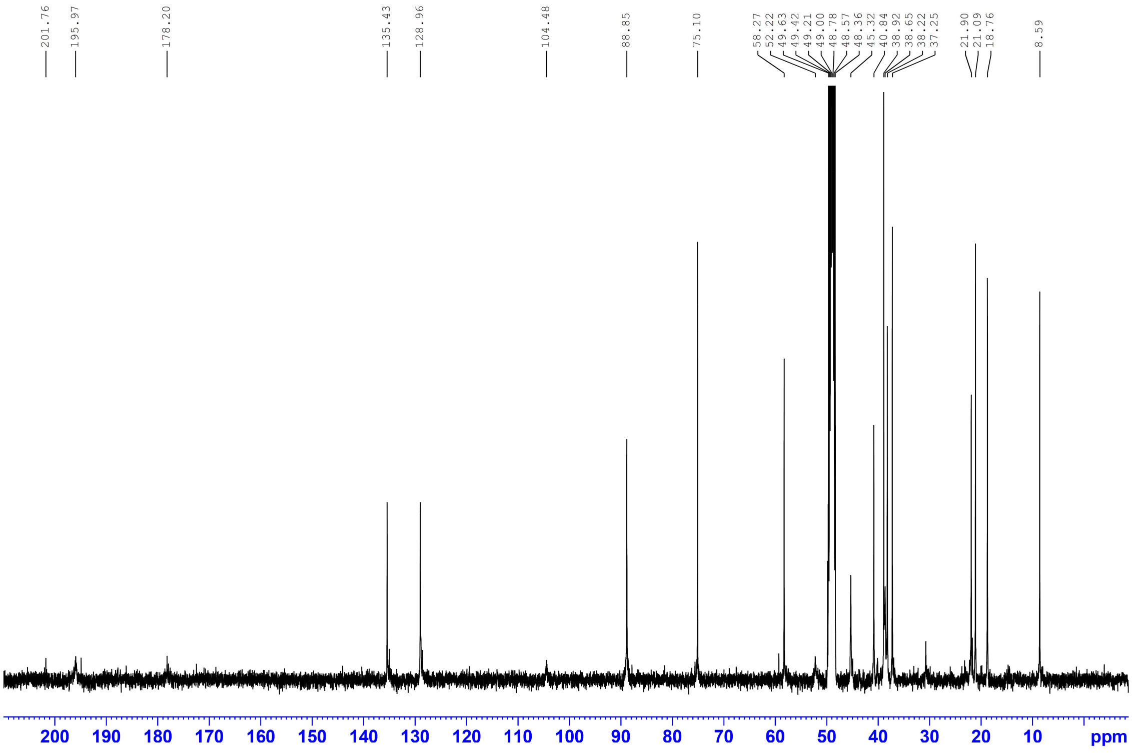

Figure S17. ^{13}C NMR spectrum (MeOH-d_4, 100 MHz) of tetramic acid **1**.

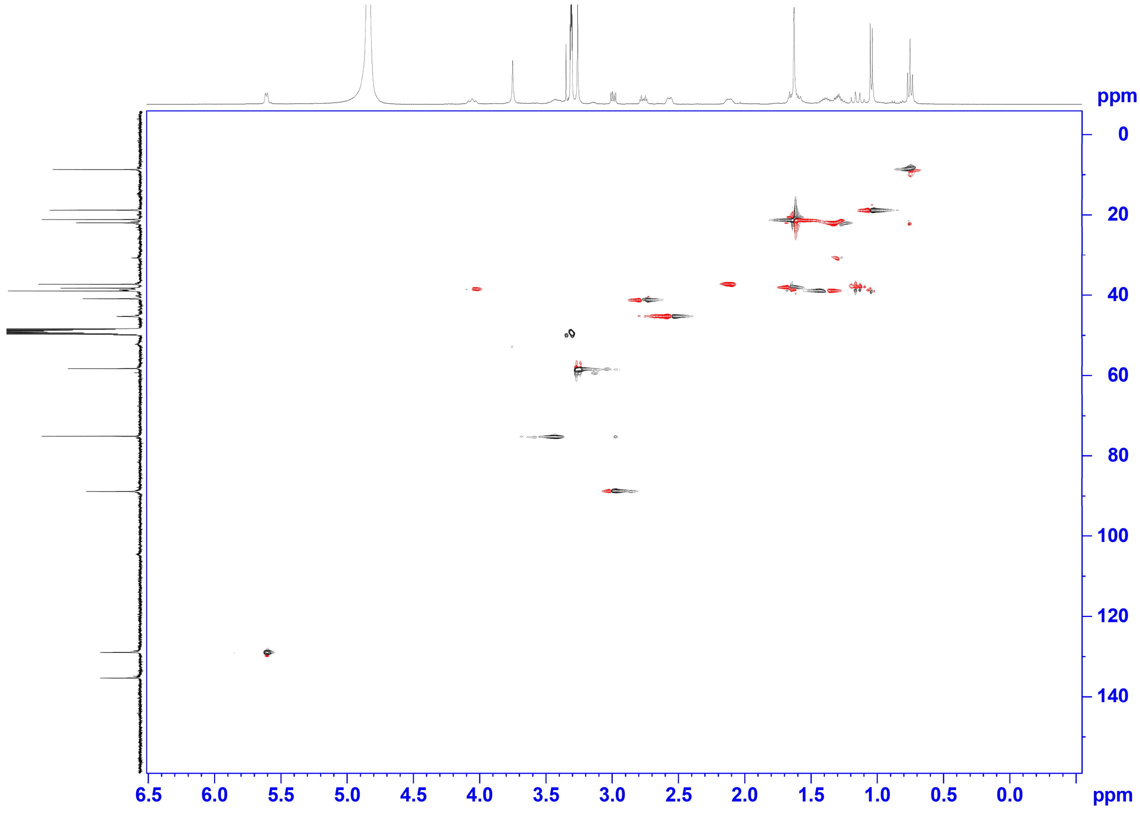

Figure S18. HSQC spectrum of tetramic acid **1**.

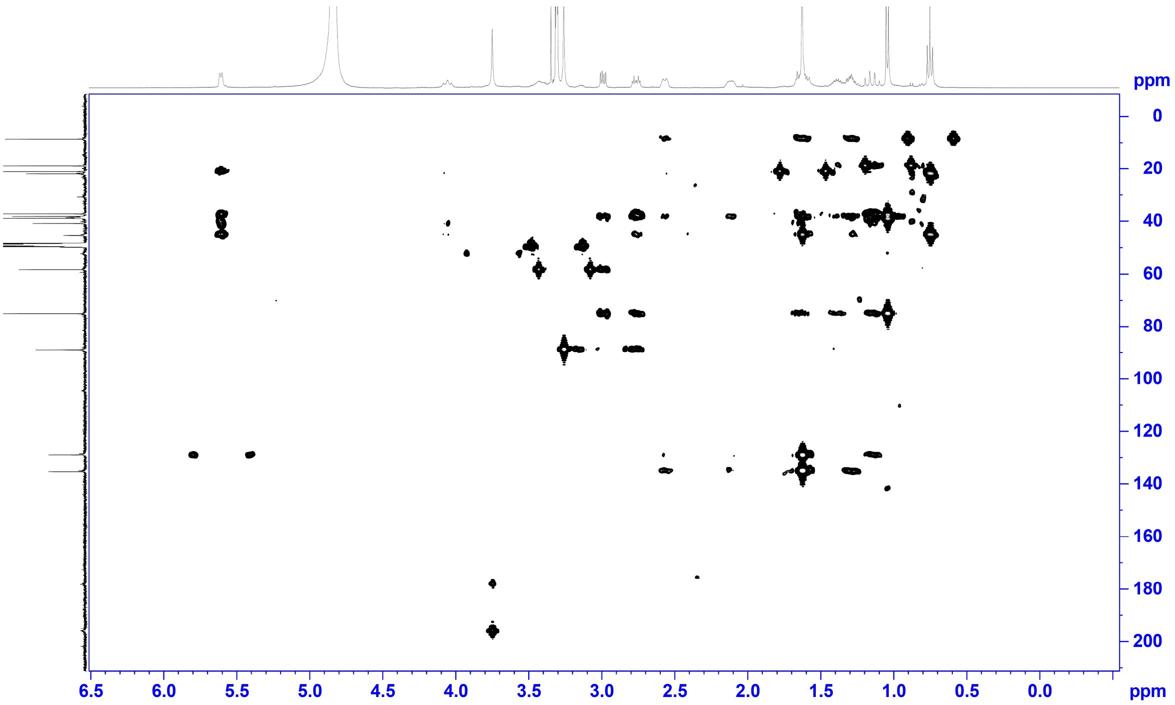

Figure S19. HMBC spectrum of tetramic acid **1**.

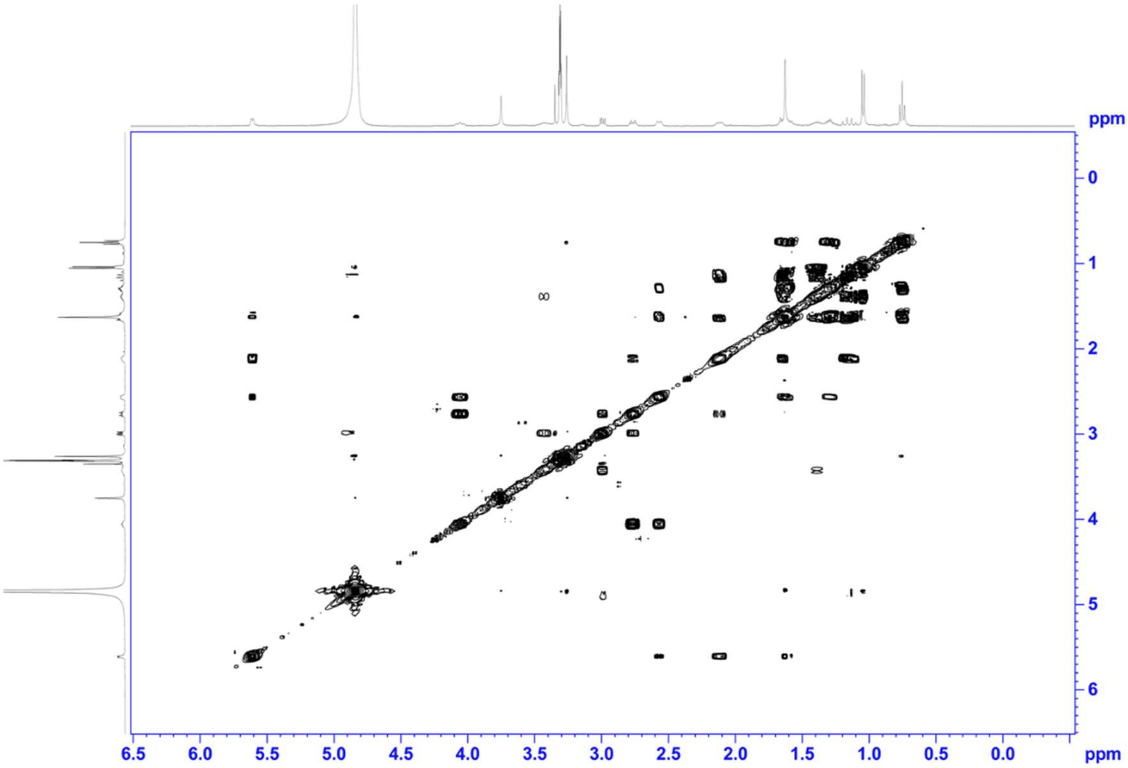

Figure S20. COSY spectrum of tetramic acid **1**.

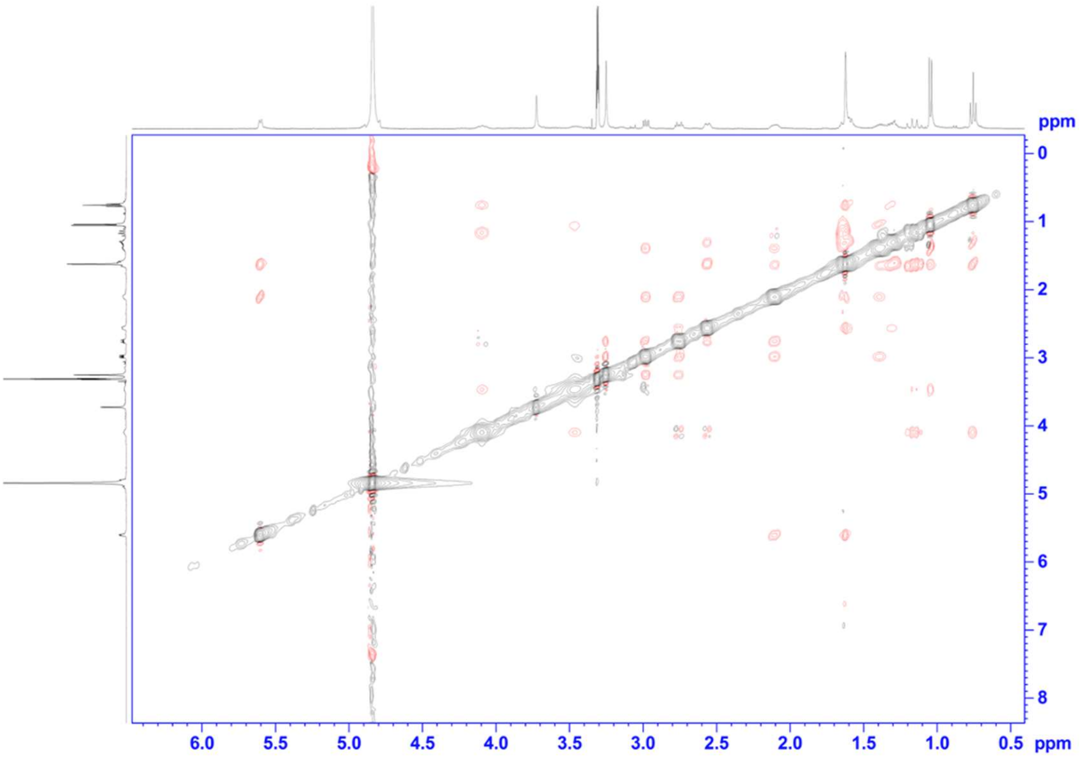

Figure S21. NOESY spectrum of tetramic acid **1**.

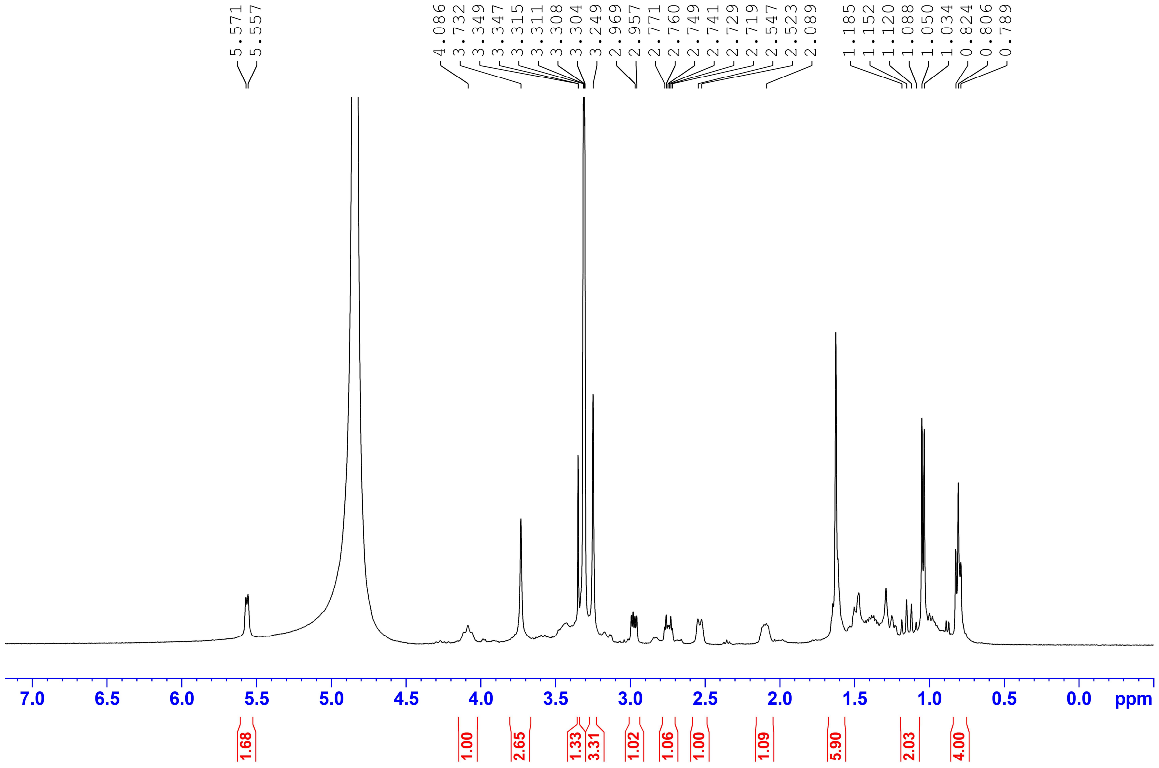

Figure S22. ^{1}H NMR spectrum (MeOH-*d*4, 400 MHz) of tetramic acid **2**.

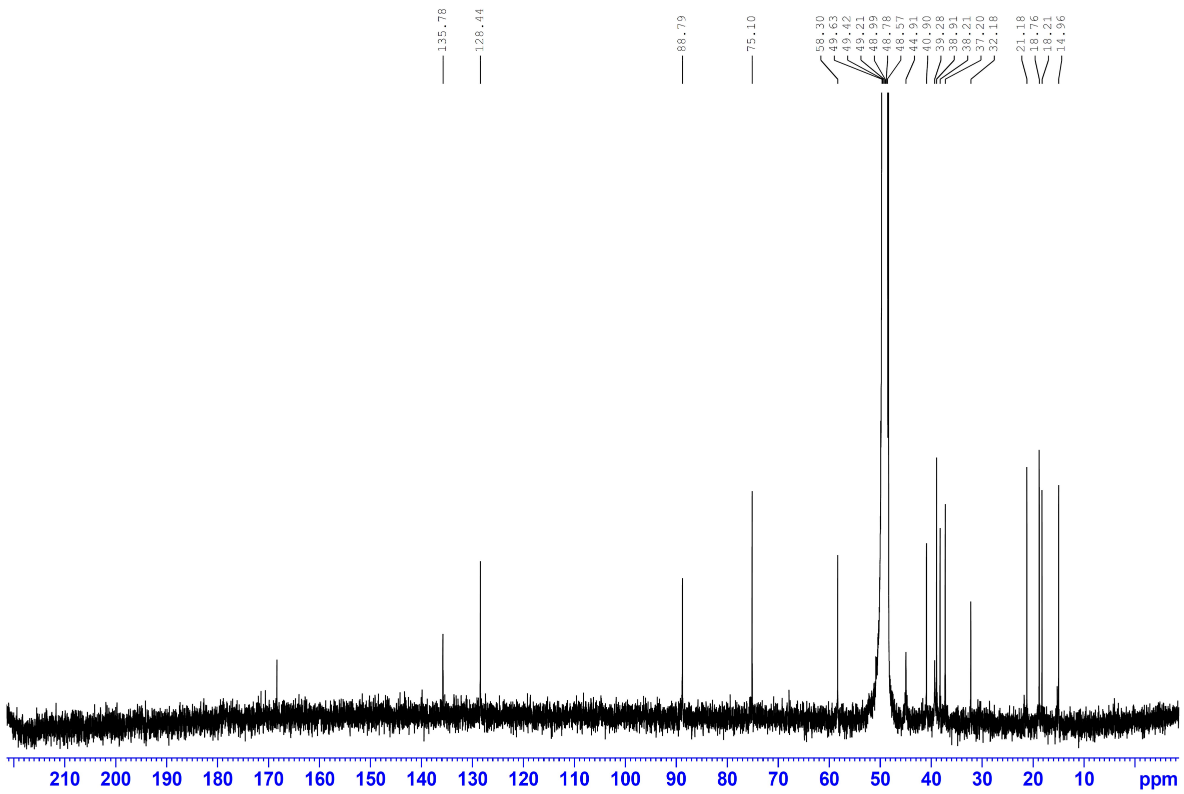

Figure S23. ^{13}C NMR spectrum (MeOH-d_4, 100 MHz) of tetramic acid **2**.

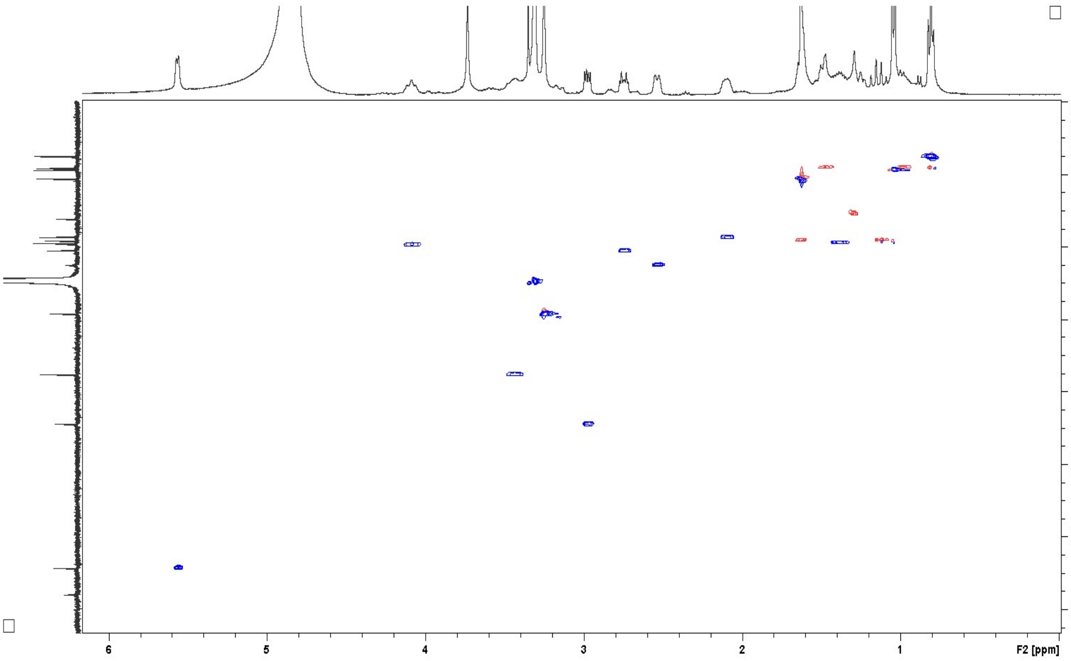

Figure S24. HSQC spectrum of tetramic acid **2**.

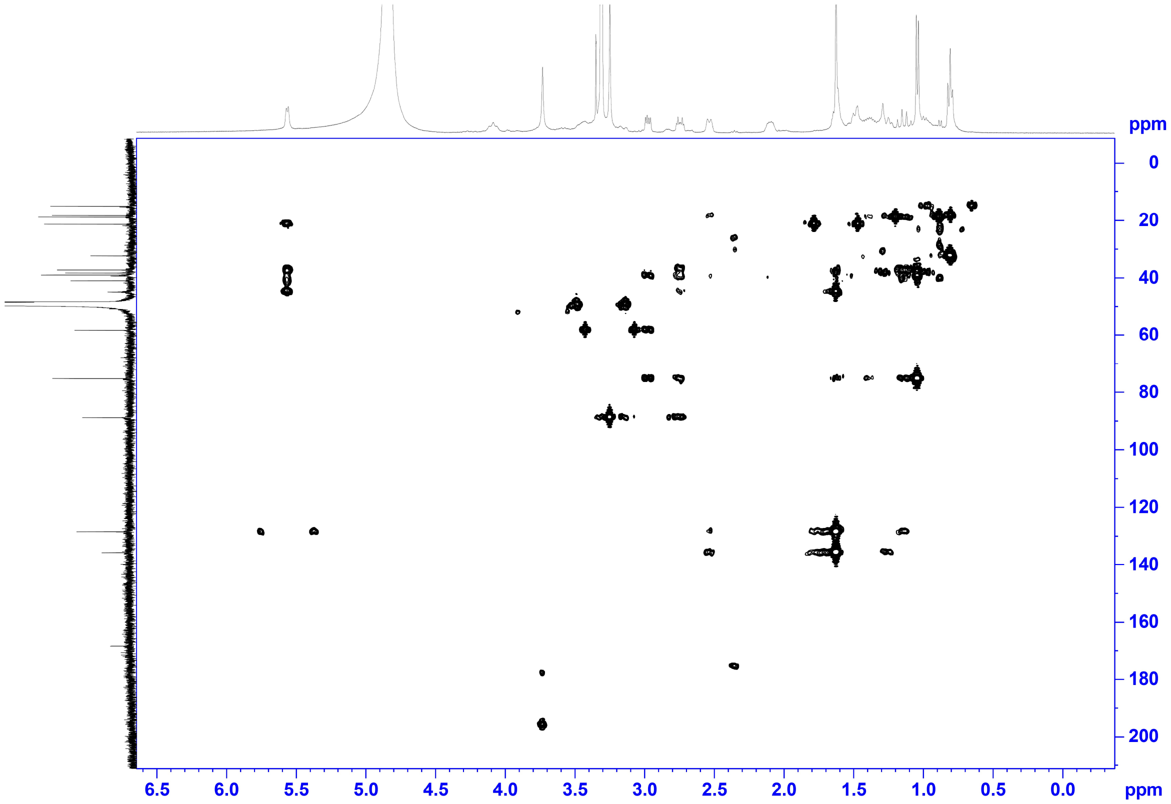

Figure S25. HMBC spectrum of tetramic acid **2**.

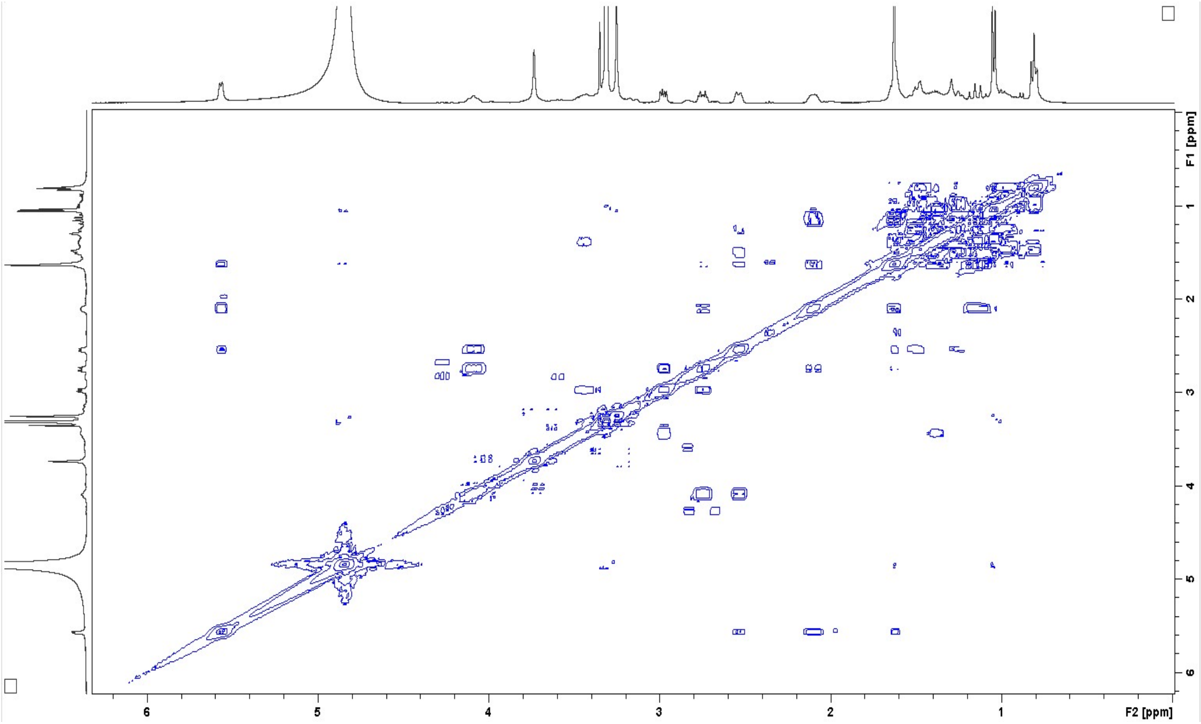

Figure S26. COSY spectrum of tetramic acid **2**.

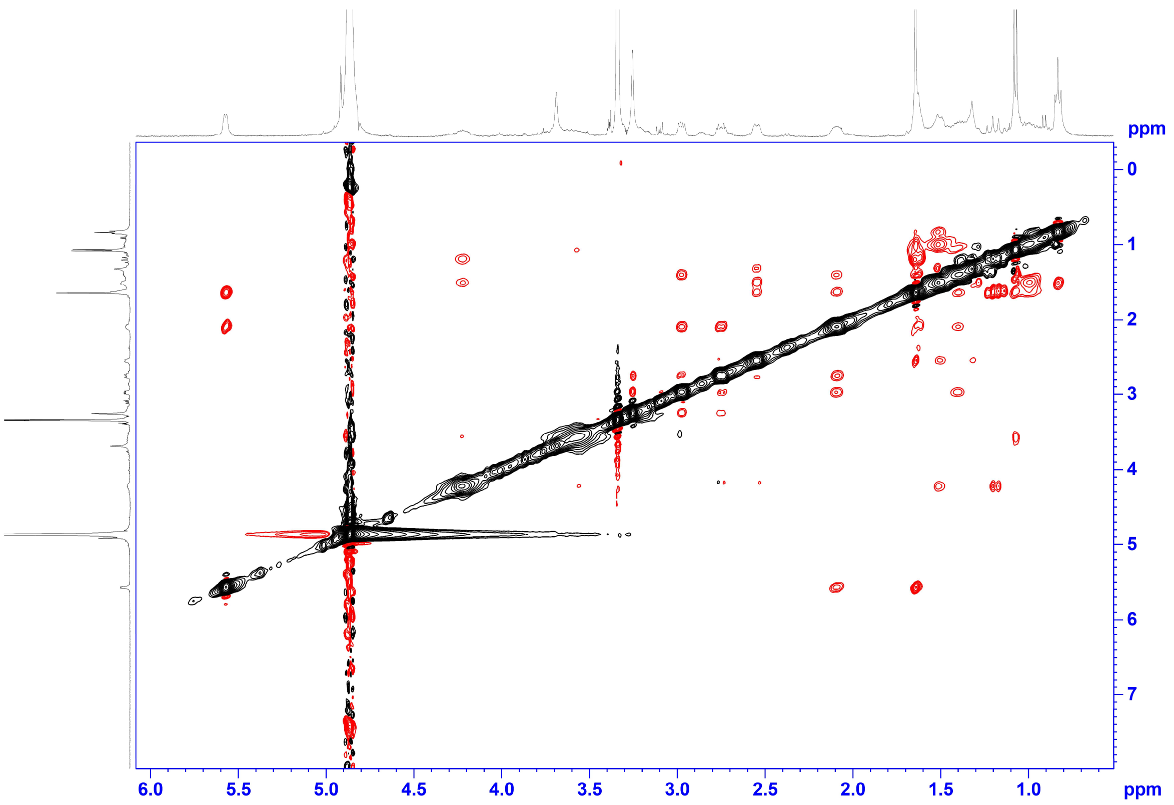

Figure S27. NOESY spectrum of tetramic acid **2.**

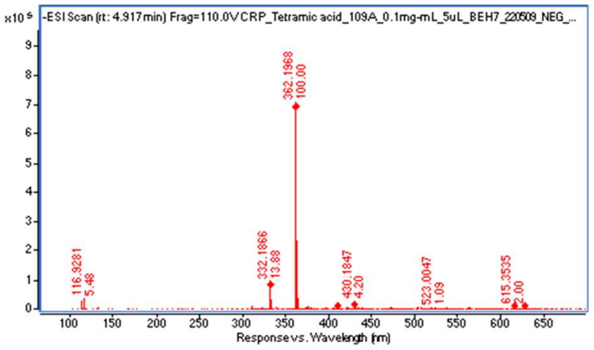

1

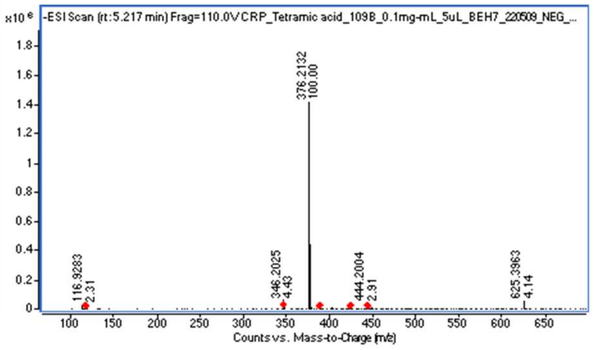

2

Figure S28. (–)-HRESIMS spectra of compounds **1** and **2.**

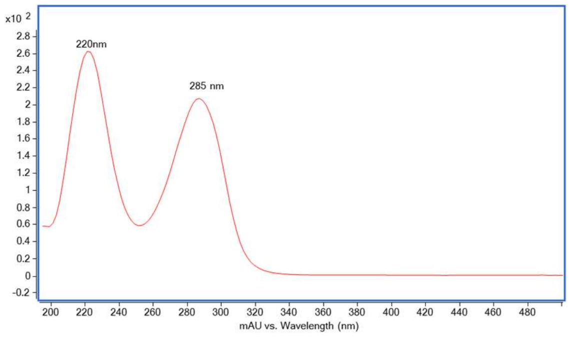

1

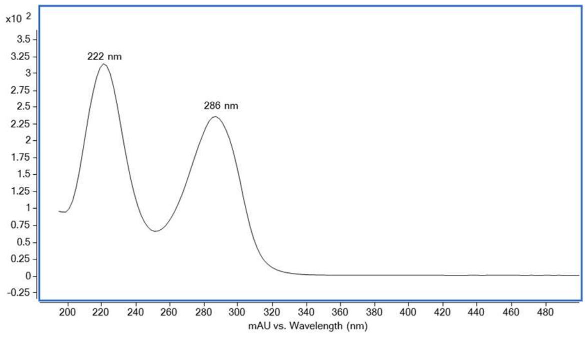

2

Figure S29. UV spectra of compounds **1** and **2**.

A

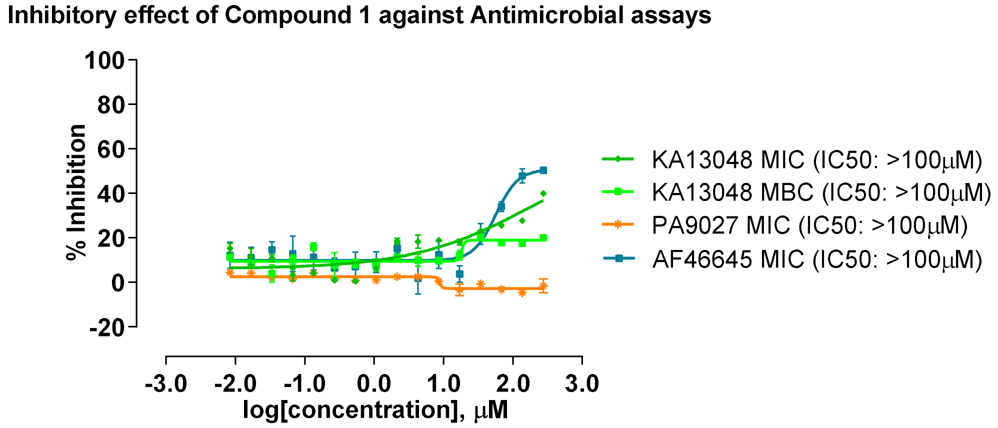

B

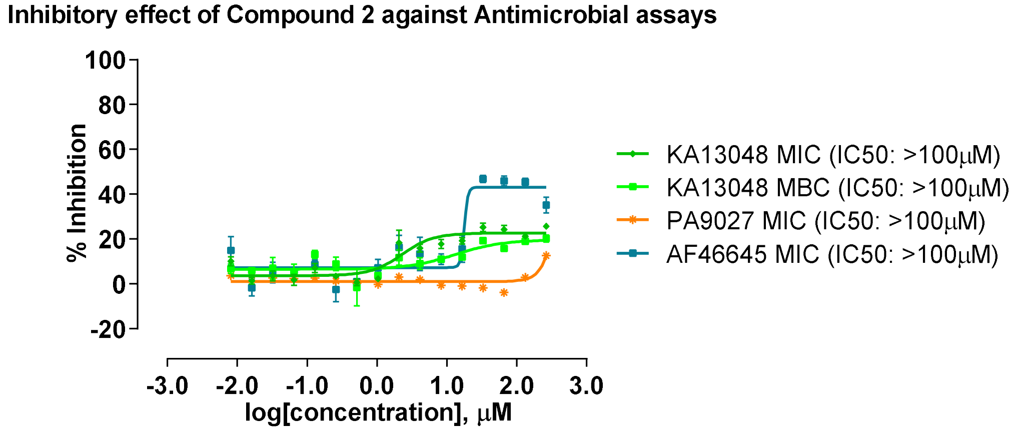

Figure S30. Dose response curve against *Klebsiella aerogenes* (ATCC® 13048™) (KA13048), *Pseudomonas aeruginosa* (ATCC® 9027™) (PA9027), *Aspergillus fumigatus* (ATCC® 46645™) (AF46645): **A)** Compound **1**, **B)** Compound **2**.

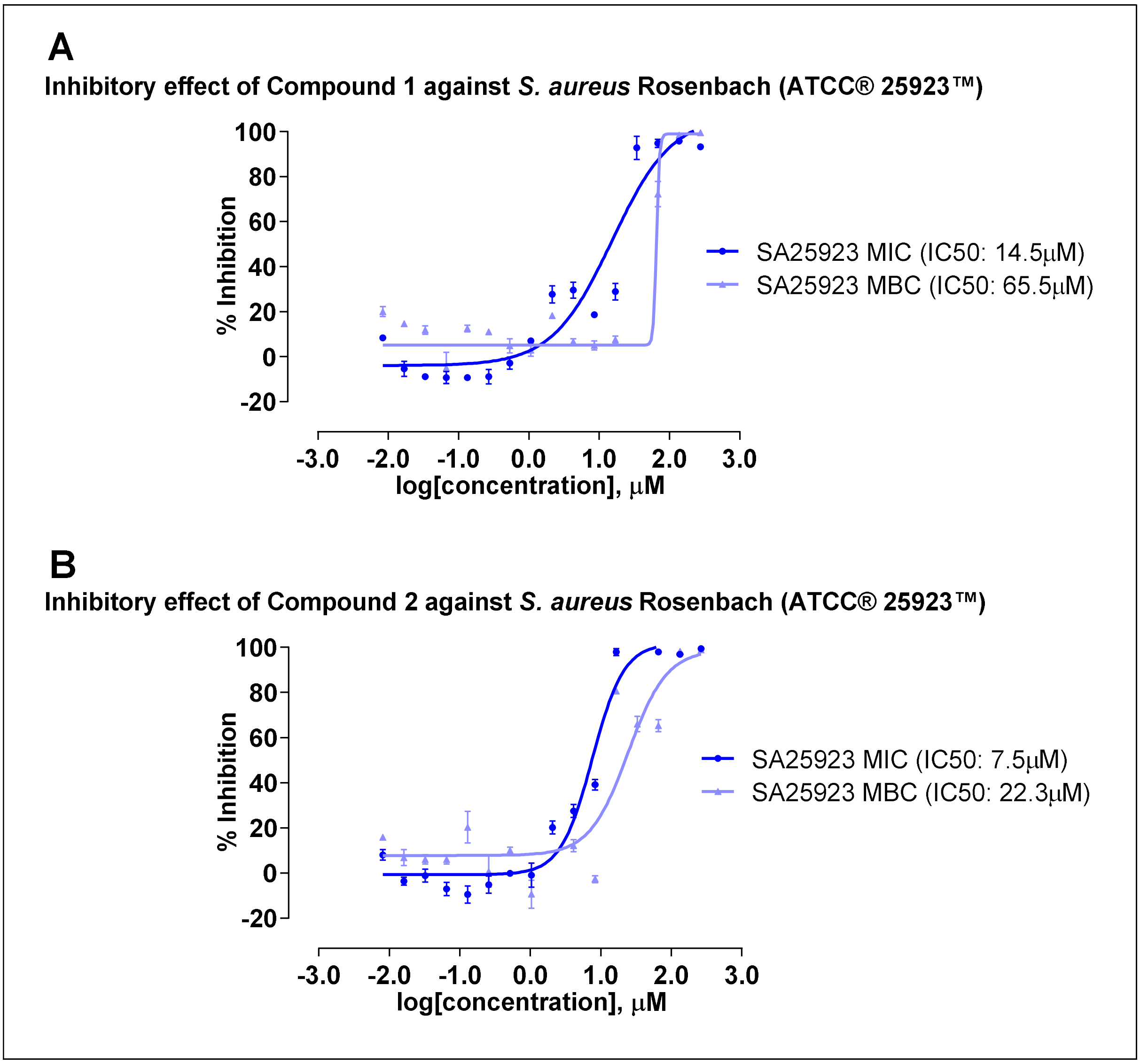

Figure S31. Dose response curve against *Staphylococcus aureus* Rosenbach (ATCC® 25923™) (SA25923), **A)** Compound **1**, **B)** Compound **2**.

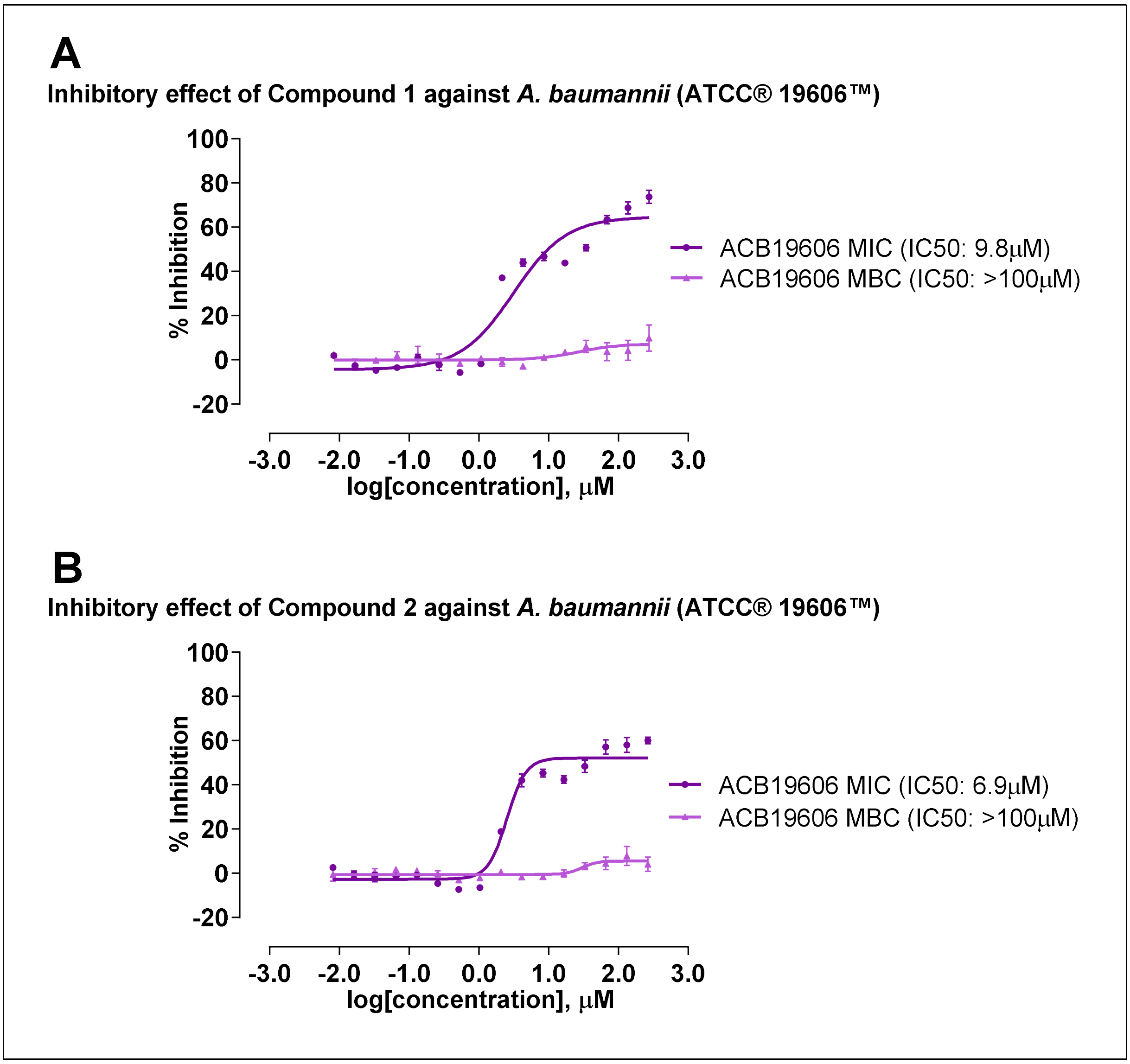

Figure S32. Dose response curve against *Acinetobacter baumannii* (ATCC® 19606™) (ACB19606), **A)** Compound **1**, **B)** Compound **2**.

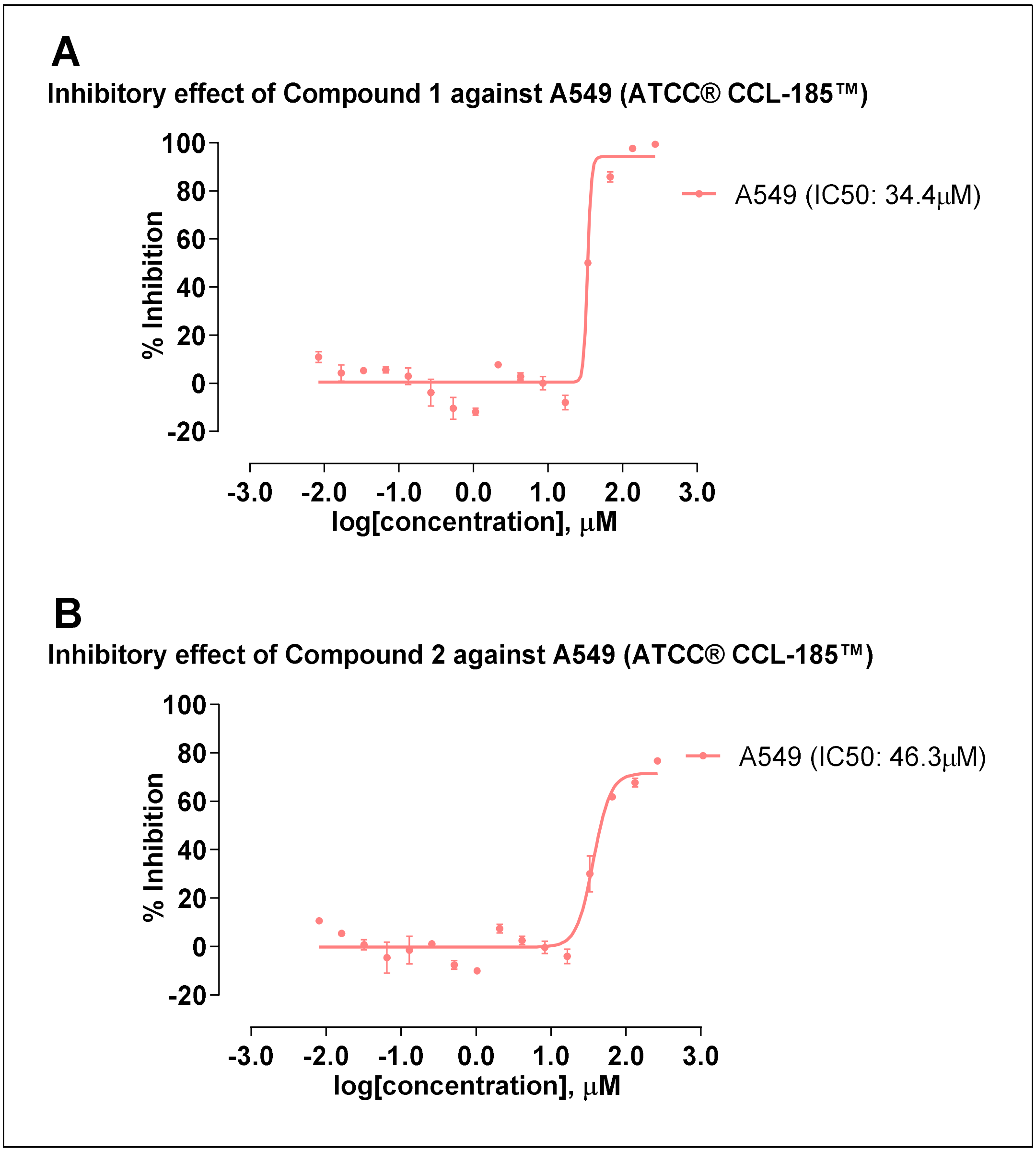

Figure S33. Dose response curve against the human lung carcinoma cells A549 (ATCC® CCL-185™) (A549), **A)** Compound **1**, **B)** Compound **2**.

Table S10. Bioactivity characterization of compounds BE-54476-A (**1**) and BE-54476-B (**2**)

	Compound 1 (BE-54476-A)		Compound 2 (BE-54476-B)	
**Target organism (ATCC® number)**	**MIC[a] (μM)**	**MBC/MFC[b] (μM)**	**MIC[a] (μM)**	**MBC/MFC[b] (μM)**
Acinetobacter baumannii (ATCC® 19606™)	9.8	>100	6.9	>100
Klebsiella aerogenes (ATCC® 13048™)	>100	>100	>100	>100
Pseudomonas aeruginosa (ATCC® 9027™)	>100	-	>100	-
Staphylococcus aureus Rosenbach (ATCC® 25923™)	14.5	65.5	7.5	22.3
Aspergillus fumigatus (ATCC® 46645™)	>100	-	>100	-
**Target cell line ATCC® number)**	**IC_{50} (μM)**			
A549 Human lung carcinoma cells (ATCC® CCL-185™)	34.4		46.3	

[a] Minimum inhibitory concentration IC_{50} for microbial assay *Acinetobacter baumannii* (ATCC® 19606™), *Klebsiella aerogenes* (ATCC® 13048™), *Pseudomonas aeruginosa* (ATCC® 9027™), *Staphylococcus aureus* Rosenbach (ATCC® 25923™) and *Aspergillus fumigatus* (ATCC® 46645™). [b] Minimum bactericidal/fungicidal concentration IC_{50} for microbial assay *Acinetobacter baumannii* (ATCC® 19606™), *Klebsiella aerogenes* (ATCC® 13048™), *Pseudomonas aeruginosa* (ATCC® 9027™), *Staphylococcus aureus* Rosenbach (ATCC® 25923™) and *Aspergillus fumigatus* (ATCC® 46645™).

Nuclear magnetic resonance (NMR) information for surfactin analogs isolated from A1123 – A100141 mutant:

A1123 - A100141 mutant was fermented in CA10LB at 28 °C for 9 days shaking at 200 rpm with 50 mm throw. At the end of the incubation period, cultures were freeze dried and surfactin analogs were extracted via preparative high-performance liquid chromatography (prep-HPLC).

Proton (^{1}H) and carbon (^{13}C) nuclear magnetic resonance spectra were recorded on a Bruker Avance 400 spectrometer with CryoProbe at 400 MHz for ^{1}H and 100 MHz for ^{13}C. The chemical shifts are reported in parts per million (ppm) on the delta (δ) scale. NMR spectra recorded in CD_3OD were referenced to the central peak of the residual methanol quintet (3.31. ppm) for 1H, or the central peak of the residual methanol septet (49.00 ppm) for 13C. NMR spectra were processed using MestReNova 10.0.2

[Leu^7] surfactin iso-C14

Peptide sequence: N-Glu-Leu-D-Leu-Val-Asp-D-Leu-Leu-C

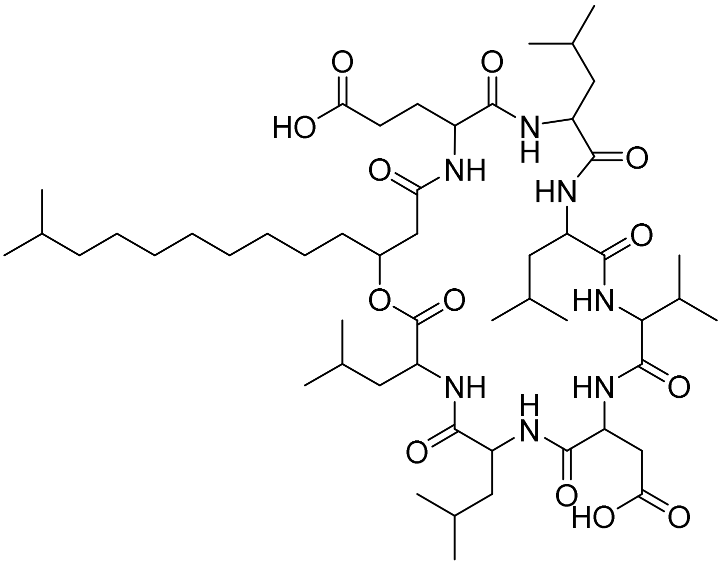

Chemical Formula: $C_{52}H_{91}N_7O_{13}$
Exact Mass: 1021.66749
Molecular Weight: 1022.33600

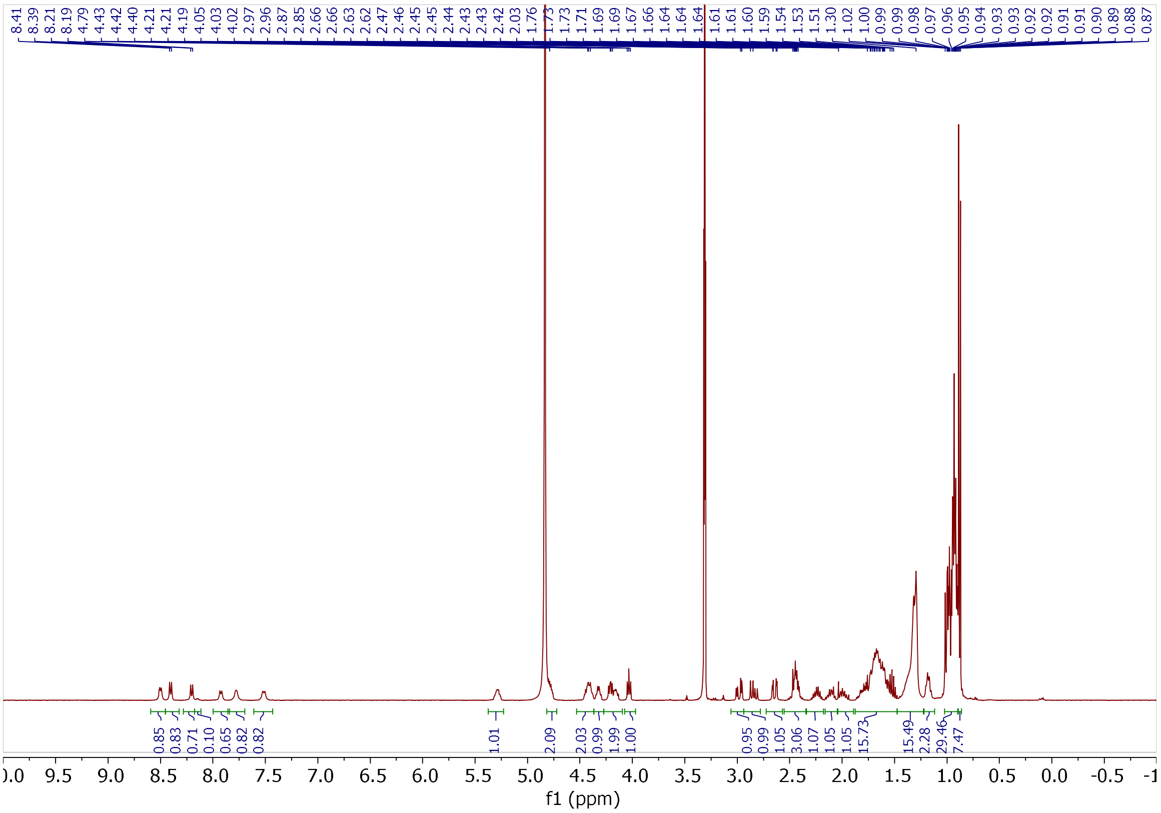

Figure S34: ^{1}H NMR spectrum of [Leu^{7}] surfactin iso-C14

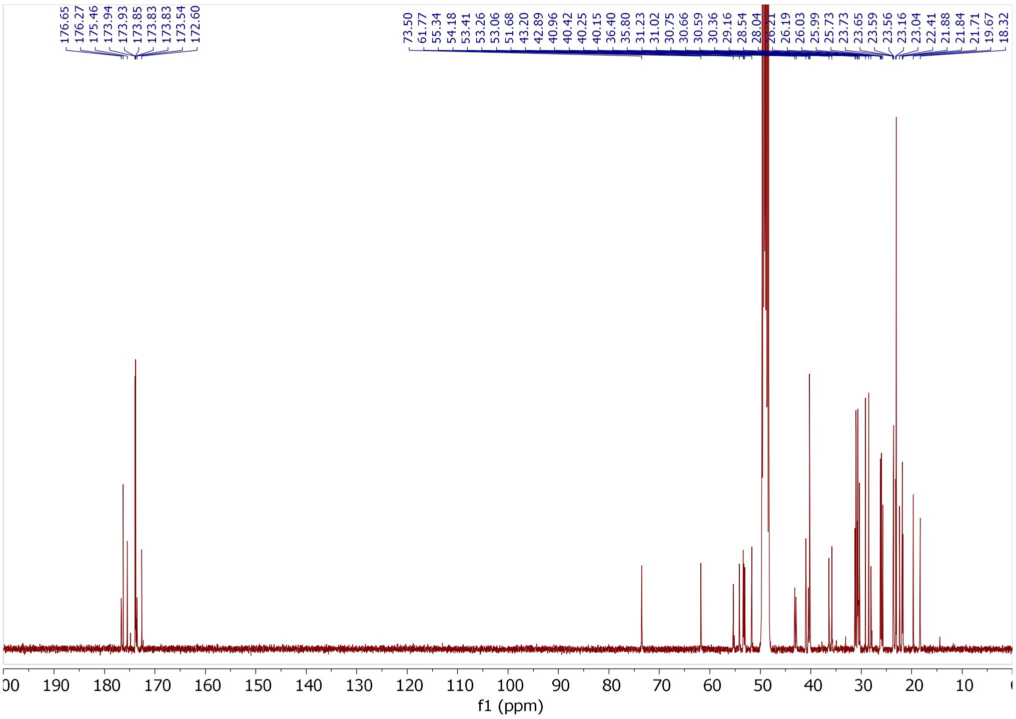

Figure S35: ^{13}C NMR spectrum of [Leu^{7}] surfactin iso-C14

[Leu^7] surfactin anteiso-C15

Peptide sequence: N-Glu-Leu-D-Leu-Val-Asp-D-Leu-Leu-C

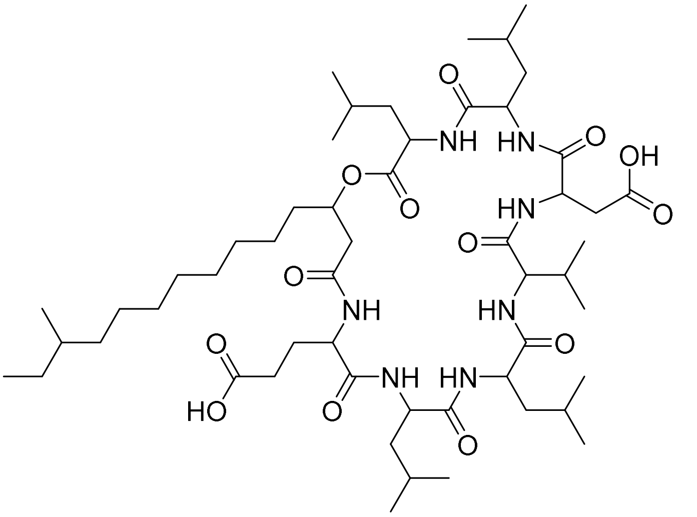

Chemical Formula: $C_{53}H_{93}N_7O_{13}$
Exact Mass: 1035.68
Molecular Weight: 1036.36

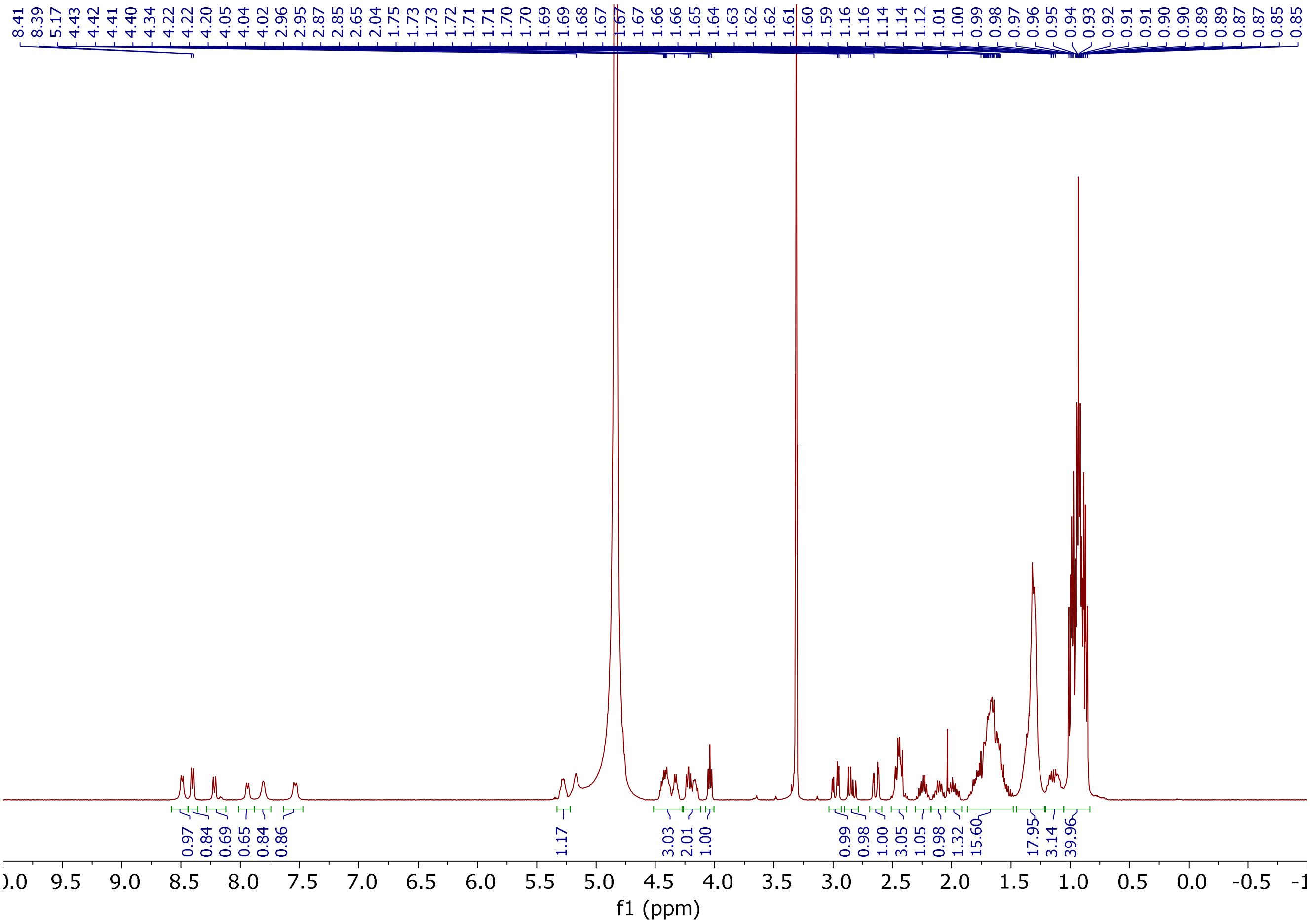

Figure S36: ^{1}H NMR spectrum of [Leu^7] surfactin anteiso-C15

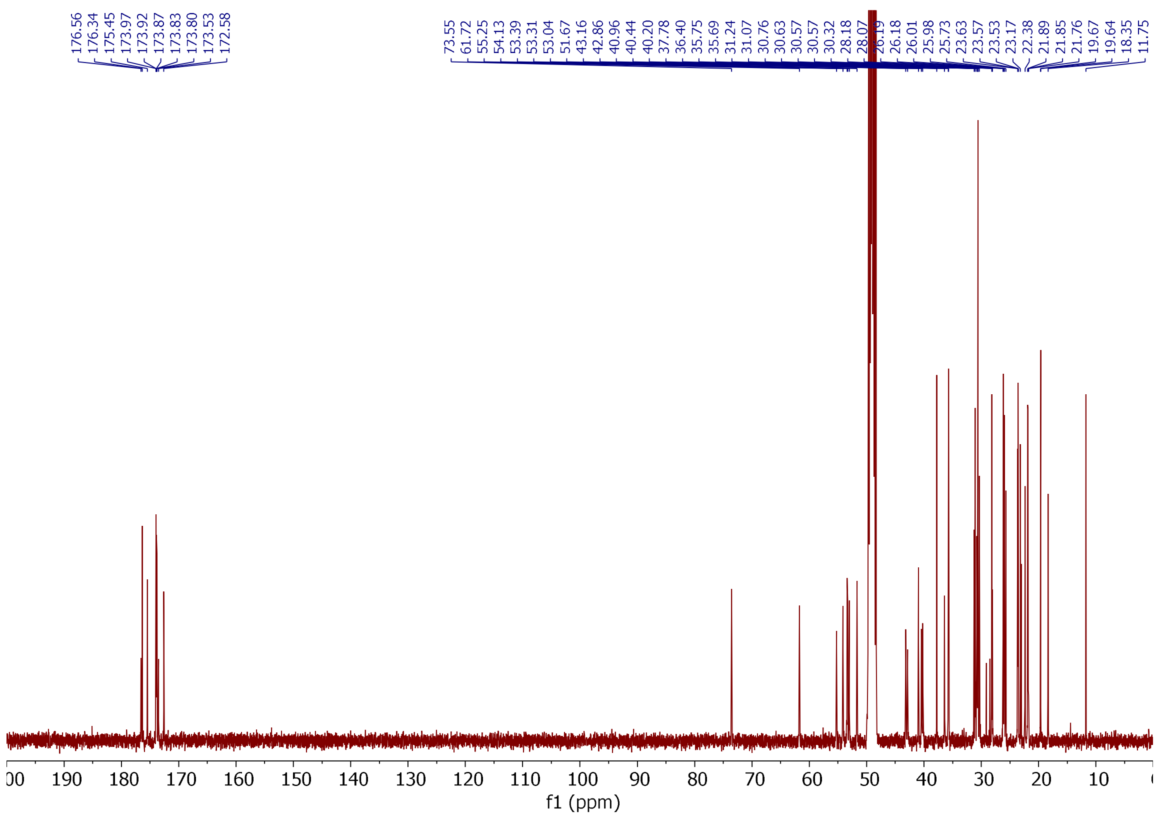

Figure S37: ^{13}C NMR spectrum of [Leu^{7}] surfactin anteiso-C15

[Leu^7] surfactin iso-C15

Peptide sequence: N-Glu-Leu-D-Leu-Val-Asp-D-Leu-Leu-C

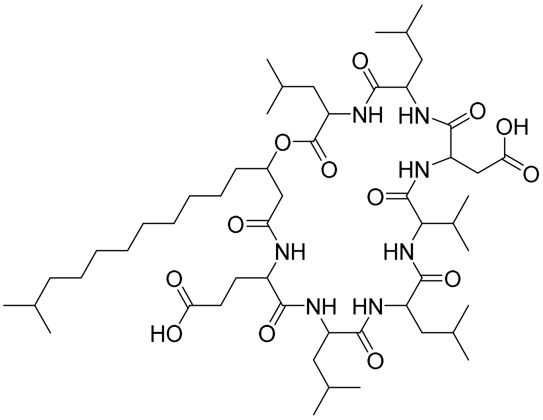

Chemical Formula: $C_{53}H_{93}N_7O_{13}$
Exact Mass: 1035.68
Molecular Weight: 1036.36

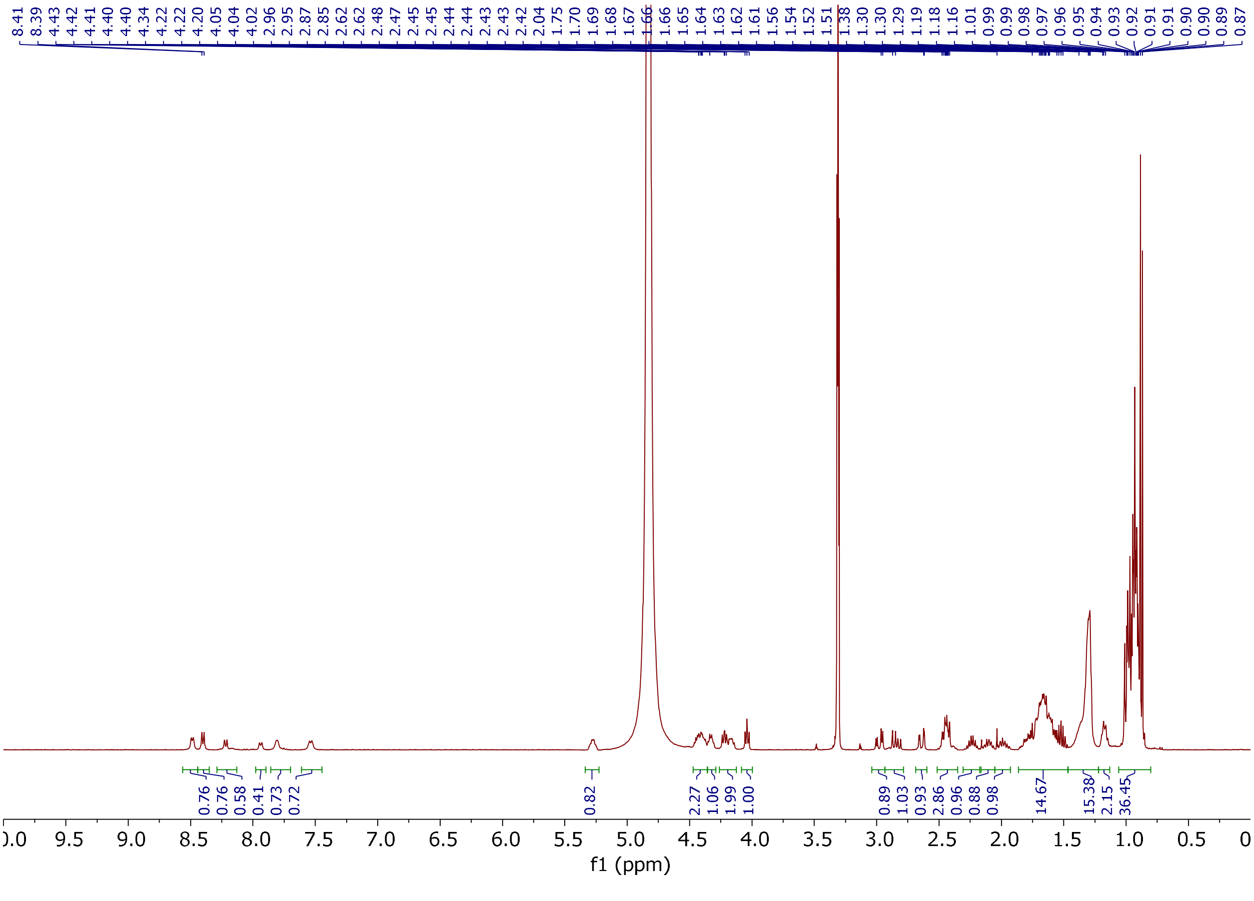

Figure S38: ^{1}H NMR spectrum of [Leu^7] surfactin iso-C15

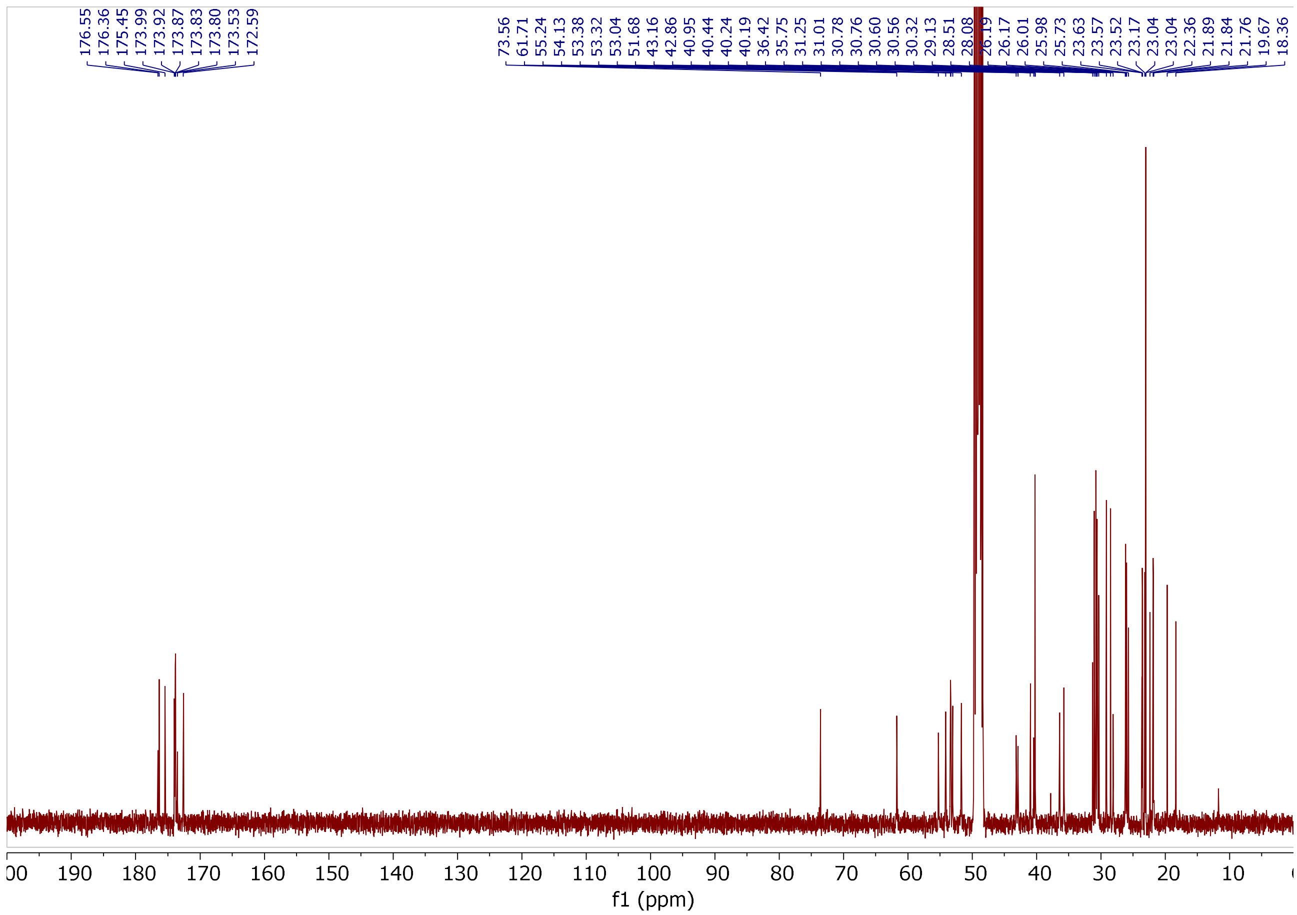

Figure S39: ^{13}C NMR spectrum of [Leu^7] surfactin iso-C15

Table S11. Integration cassette sequences: *kasO**p is underlined

Integration cassette	Sequences (DNA)
kasO*p-*SCO2792* (AdpA)	TGTTCACATTCGAACGGTCTCTGCTTTGACAACATGCTGTGCGGT<br>GTTGTAAAGTCGTGGCCAGGAGAATACGACAGCGTGCAGGACTG<br>GGGGAGTTATGAGCCACGACTCCACCGCCGCGCCGGAAGCCGC<br>GGCCCGGAAGCTGTCCGGGCGACGCCGCAAGGAGATCGTCGCG<br>GTGCTGCTGTTCAGCGGCGGCCCCATCTTCGAGAGTTCCATACC<br>GCTGTCGGTGTTCGGGATCGACCGCCAGGACGCCGGCGTGCCG<br>CGCTACCGGCTGCTGGTGTGCGCCGGCGAGGACGGCCCGCTGC<br>GGACCACGGGGGGCCTGGAACTCACCGCGCCGCAGGGACTGG<br>AGGCGATCTCGCGCGCGGGCACGGTCGTCGTGCCGGCCTGGC<br>GGTCGATCACCTCGCCGCCGCCGGAGGAAGCACTCGACGCACT<br>GCGCAGGGCGCACGAGGAGGGGGCGCGCATAGTCGGACTGTG<br>CACGGGCGCCTTCGTCCTCGCGGCGGCCGGCCTGTTGGACGGC<br>CGCCCCGCCACCACGCACTGGATGTACGCGCCGACGCTGGCCA<br>AGCGCTATCCGTCGGTGCACGTCGACCCGCGCGAACTCTTCGTG<br>GACGACGGCGACGTGCTGACGTCCGCCGGCACCGCGGCCGGG<br>ATCGACCTGTGCCTGCACATCGTGCGCACGGACCACGGCAACGA<br>GGCGGCCGGTGCGCTGGCCCGCCGGCTGGTGGTCCCCCCGCG<br>CCGCAGCGGCGGCCAGGAGCGCTACCTCGACAGGTCTTTACCG<br>GAGGAGATCGGCGCCGACCCGCTCGCCGAGGTCGTCGCCTGGG<br>CGCTGGAGCACCTCCACGAGCAGTTCGACGTGGAGACGCTGGC<br>CGCGCGCGCCTACATGAGCCGCCGCACCTTCGACCGCCGGTTC<br>CGCTCGCTGACGGGCAGCGCCCCGCTCCAGTGGCTGATCACCC<br>AGCGGGTGCTCCAGGCGCAGCGCCTGCTGGAGACGTCGGACTA<br>CTCGGTGGACGAGGTCGCCGGGCGCTGCGGCTTCCGCTCCCCG<br>GTGGCCCTGCGCGGCCACTTCCGCCGCCAGCTGGGCTCGTCCC<br>CGGCCGCCTACCGGGCGGCCTACCGGGCGCGCCGTCCCCAGG<br>GCGACCGCCAGCCGGACCCGGACACCGCAGCCGCGGGCGCCA<br>CCCGCCCGCTGCCCCCGTCCGACCCGCCGGCCTCCCTCGCCCC<br>GGAGAACGCGGTCCCGTTCCAGACCCGCCGCACCGCCACCCCG<br>ATGCCGGCGGGCGCGGCCAGCGTCCCGGGCCAGCGCAGCGCG<br>CCGTGAGGTGAGCAGACGGCTCCCTCGAACGCCGGACGGGCCG<br>GAGCGTGCCCGTCCGGCGTTCCCGTCCGGTGCCGGGCGGGGC<br>ACACTCAGCCCGTCCGGCGTTCA
*kasO**p -*SCO3571* (Crp)	TGTTCACATTCGAACGGTCTCTGCTTTGACAACATGCTGTGCGGT<br>GTTGTAAAGTCGTGGCCAGGAGAATACGACAGCGTGCAGGACTG<br>GGGGAGTTATGGACGACGTTCTGCGGCGCAACCCGCTCTTCGC<br>GGCGCTCGACGACGAGCAATCCGCGGAGCTCCGCGCCTCCATG<br>AGTGAGGTGACCCTCGCCCGCGGCGACACCCTGTTCCACGAGG<br>GGGACCCCGGAGACCGCCTCTACGTGGTCACGGAAGGCAAGGT<br>CAAGCTCCACCGCACGTCCCCCGACGGGCGCGAGAACATGCTG<br>GCCGTCGTCGGCCCCAGCGAGCTGATCGGCGAGCTGTCGCTCT<br>TCGACCCGGGCCCGCGCACGGCGACCGGCACCGCGCTGACCG<br>AGGTCAAGCTGCTCGCCCTCGGCCACGGCGACCTCCAGCCCTG<br>GCTGAACGTCCGCCCCGAGGTGGCCACCGCGCTGCTGCGCGCC<br>GTCGCGCGCCGCCTGCGCAAGACCAACGACGCCATGTCGGACC

	TGGTCTTCTCGGACGTTCCCGGCCGTGTCGCCCGCGCCCTGCTC<br>GACCTCTCCCGCCGCTTCGGCGTGCAGTCGGAGGAGGGCATCC<br>ACGTGGTGCACGACCTGACGCAGGAGGAGCTGGCCCAGCTGGT<br>CGGCGCGTCCCGCGAGACGGTCAACAAGGCGCTGGCGGACTTC<br>GCCCAGCGCGGCTGGCTCCGCCTGGAGGCCCGCGCGGTGATC<br>CTCCTGGACGTGGAGCGACTGGCCAAGCGCTCCCGCTGA
*kasO**p -*SCO4069* (SarA)	TGTTCACATTCGAACGGTCTCTGCTTTGACAACATGCTGTGCGGT<br>GTTGTAAAGTCGTGGCCAGGAGAATACGACAGCGTGCAGGACTG<br>GGGGAGTTATGACCGGTATCGGTGGTGTGGAGATGGGTGCTCA<br>GGCCGCGCGTGCCCGGGCACTGGCGGTGCTGCGGGTCCGCAG<br>CCGCGCGCTGGCCGTGGCCCTGCTGCCCGCCGCCGTCGCCGT<br>GATCCTCCTCGTCGGCGGCTCCACCGGGCACCTGGCGGGCGCC<br>GCCTGGGACGTCCTGCGCTGGGTCGTGTCCGTCCTCGCGCTGC<br>TCGTCCTGCTGGCCGCCGCCGGAGTCGCCCTGGTCGTGGCCCG<br>CTCCCGGCCCGCCGTGACCCCCACGGTCACGGTCGCCGAGGAG<br>TCGGCGCCCGACCTCTACCGGATGGTGCGCGACCTGGCCGACC<br>GCCTCGACGTCCCCGCCCCCTCCGCGATAGCGCTCACCCCGGA<br>CTGCGACAGCTGGCTGGAGGACCGCACCCACCCGGCCCACGGC<br>CCGCCCCCGGCGGAGCGGCACGACGACGACCCGGCCGGCCTG<br>AGCGGCCCCGCCCACCGCCGGAGCCCGGCCGCCGCGCCCGTC<br>CTGGTCATCGGCTCACCGTTCCTGTGGTGGATGCGGGTGGGCG<br>AGCTGCGCGCCGTCCTCGCCCCGGTCGTCGCCGGTACGGGGCC<br>CTCGGCGCACCCCGACATAGCCGCGGCCCGGCGTTTCATACGG<br>GGCCTGGACGCCGCGGTGGCGGTGGCCTCGGCAGGCCGACGA<br>GATCCGCTGTCCCGCGTGGCGTGCGCGGGCCTCGGCTGGGCGT<br>CCCGGCTGCTGCTGCGCGGTTGCCGGGATCACGCGACCGAGAT<br>GGAGCGCGGGGTCGCGGCCGCGGCCGCCGAGCGTGCACAGGC<br>TGTGGACTACGGTCTGCGGATCGTCGCGCAGGAGCAGGTCGGG<br>CTCGCGTACGCGGGCTGGGACCGGCTGCTGACCCGGGTGGCG<br>CTGCCGGCCTGGCGGATGGGCCGCTGGCCCTCCCGGCTGGAC<br>GTCGGTGTCGTCGCCGCCCTCACGGAGCTGTCGCGCCGGGACC<br>GCCTGGCCGAGGGCTTCGCCTCCCGCCTCGGCGAGCGCCCCGC<br>CTGTGACCTGCTGGAGGAGCCCGGGGTGATGGACGAGGCGGTC<br>TCGCTCCTCGCCGCGCGTCTGTTCCACGGCGGGCCCGCCGAGA<br>CCGGCCCGGACTGGTCGCCGGTGGGGTGGGACGAGTATCCCGA<br>GGAGGTCGTCGACCGGACCTGGCGCACGGACGCGGCCCGCCT<br>GCACCGCGTGCTGGACACCCTGGGCGTCCGCCGCGCCGCCGG<br>GGGCGTCGCTCCGGGCACGGACGGCCCGACCCTGGCCCGCGT<br>CCTGGACCACCTCACGACCCCGGACAACGCGGTCACCGTCCCC<br>CCTGTCACCGGTGCCGGCACCGGCACCGAGGCGGCCGGCGAG<br>GCACGGACGGAGGCATCGGCCGGGGGCGGCACCCCGCATCCA<br>CACGTGCCGTACGCGGACGACGAAGCCCCGCATGCGCCGTACG<br>CGGACGACGAAGCCCTGCACGTGCCGGACGCCGACGACGAAGC<br>CCCACATGCGCCGTACGCCGACCACGCCGACCACGCCGCCTAC<br>GACGAGGGCGACGAGGACGACGAGGACGATCTCGCCGGCACCA<br>CCGCCCGTGGCGCCGCCCTGGCCGCCGGGCTCAGCGCGCAGC<br>TGGCCCGCGAGGAGGCTCGGGCGCGGCCCGACGCGGCCGGGG<br>CCACGTCGCTGACTCCGCAACCGGGAGCCGGAGCCGGTCCCGA<br>CGCGGCCCTCTGGGACGACCGCGTGCTCCCCCTCCTCCCGCTT<br>CAGCCCCCGCGCACCGGGCGCGAGATCCTCACCGCGCACATCA<br>CCGCGATGGTGTGCTGCGCGGCGATGGACACCGCCGGGGCGG

	CCCCCGGCCTCGACTGGCTCGACGGGCCCTCGCTGCTGTTCGA<br>CGGCGCCCGCACCGCGGACCTGGCGCCCAGCGTGCTCAGCCTC<br>GTCGAGACCGGCGACCCGGGGCCGCTGCGCGCCTGGATGACC<br>GACCTCGGCATACGTCCCGAGAAGCCGGTGCGTCTGGTCTGA
*kasO**p - *SCO6196* (FAS)	TGTTCACATTCGAACGGTCTCTGCTTTGACAACATGCTGTGCGGT<br>GTTGTAAAGTCGTGGCCAGGAGAATACGACAGCGTGCAGGACTG<br>GGGGAGTTATGACCGCACCCGCGCCCCAGCCGTCGTACGCGCA<br>CGGCACCAGCACCACCCCGCTGCTCGGCGACACCGTCGGCGCC<br>AACCTCGGCCGCGCGATCGCCGCCCACCCGGACCGCGAGGCCC<br>TCGTCGACGTCCCGTCCGGACGGCGCTGGACCTACGCCGAGTT<br>CGGCGCCGCCGTCGACGAGCTGGCCCGGGGGCTGCTCGCGAA<br>GGGCGTCACCAGGGGCGACCGGGTCGGCATCTGGGCGGTCAAC<br>TGCCCCGAGTGGGTCCTCGTCCAGTACGCCACCGCCCGCATCG<br>GCGTCATCATGGTGAACGTCAACCCGGCCTACCGGGCCCACGA<br>GTTGGAGTACGTTCTCCAGCAGTCCGGCATCAGCCTGCTGGTCG<br>CCTCCCTCGCCCACAAGAGCAGCGACTACCGGGCGATCGTGGA<br>GCAGGTCCGCGGCCGCTGCCCCGCGCTGCGCGAGACCGTCTAC<br>ATCGGCGACCCGTCCTGGGACGCGCTCACCGCGGGCGCCGCC<br>GCGGTGGAGCAGGACCGGGTCGACGCCCTCGCCGCCGAACTGA<br>GCTGCGACGACCCGGTCAACATCCAGTACACCTCCGGCACCACC<br>GGCTTCCCCAAGGGCGCCACCCTCTCCCACCACAACATCCTCAA<br>CAACGGCTACTGGGTGGGCCGCACGGTCGGCTACACGGAGCAG<br>GACCGGGTCTGTCTGCCGGTGCCCTTTTATCACTGCTTTGGCAT<br>GGTGATGGGGAATCTGGGTGCCACCTCCCACGGCGCCTGCATC<br>GTCATCCCCGCCCCGTCCTCCGAGCCGGCGGCCACGCTGGAGG<br>CGGTGCAGCGGGAGCGGTGCACGTCCCTGTACGGCGTCCCGAC<br>CATGTTCATCGCGGAGCTGAACCTGCCGGACTTCGCCTCCTACG<br>ACCTCACCTCCCTGCGCACCGGCATCATGGCGGGCTCGCCCTG<br>CCCGGTGGAGGTGATGAAGCGGGTGGTCGCCGAGATGCACATG<br>GAGCAGGTCTCCATCTGCTACGGCATGACCGAGACCTCCCCGGT<br>CTCCCTGCAGACCCGCATGGACGACGACCTCGAACACCGCACC<br>GGCACCGTCGGCCGCGTCCTGCCGCACATCGAGGTCAAGGTCG<br>TCGACCCGGTCACCGGCGTGACCCTGCCGCGCGGCGAGGCGG<br>GCGAACTGCGCACCCGCGGCTACAGCGTGATGCTCGGCTACTG<br>GGAGGAGCCCGGGAAGACCGCCGAGGCCATCGACCCGGGCCG<br>CTGGATGCACACCGGGGACCTCGCGGTGATGCGCGAGGACGGA<br>TACGTCGAGATCGTCGGCCGCATCAAGGACATGATCATCCGGGG<br>CGGCGAGAACATCTACCCGCGCGAGGTCGAGGAGTTCCTGTAC<br>GCCCACCCGAAGATCGCGGACGTCCAGGTCGTCGGCGTCCCGC<br>ACGAACGCTACGGCGAGGAGGTCCTGGCCTGCGTCGTCGTGCG<br>CGATGCCGCCGACCCGCTCACCCTGGAGGAACTGCGCGCCTAC<br>TGCGCCGGGCAGCTCGCCCACTACAAGGTGCCCAGCCGCCTCC<br>AGCTCCTCGACTCCTTCCCGATGACCGTCTCGGGGAAGGTGCGC<br>AAGGTGGAGTTGCGGGAGCGGTACGGAGCGCGCCCCTGA
*kasO**p - *SLIV09220* (RedD)	TGTTCACATTCGAACGGTCTCTGCTTTGACAACATGCTGTGCGGT<br>GTTGTAAAGTCGTGGCCAGGAGAATACGACAGCGTGCAGGACTG<br>GGGGAGTTATGACGGGTGGGGGAGTGCTTGCCACGATGGACCC<br>GGTTCGAAAACTGGTCCGGAGCCAGCCAAAGATCGGGCGGCAC<br>CCCGTCGCGGCCGGTCAGGACGGCCGCGACCGGCACCCCATCC<br>GCTCATGGGAGTGCGGAGAACGCGCGAGGACGGCCCGGACCG

	GCAGGACGGTCGGCCGCGCGGCCGACCCCTCGGACCACGGAC<br>CCAGCCTGTACAACTTCGGGGGATGCGTGGAAATCAACATATTG<br>GGACCCGTATCGATCGACACGTCGCACAGCGGCGGCGGCATCC<br>GGGCCGGCAAGGTCCGTACGCTGGTGGCGACGCTCGCCATCGA<br>CGCGGGCCGCGCCGTGTCGCTCGCGGACCTGGTGGACGAACTG<br>TGGGGCGCGACCCCGCCCGACAACGTCCTCAACGCCCTCCAGG<br>CGCATGCCGCTCGGGCCCGGAAAGTGCTCAACGAGCGTGCCTG<br>CCCCGAGCGGGCGGGCGGCATCCTGCGTTCCGTGCTCGGCGG<br>CTACCTGCTGGAGATCGACCCGCAGTGCGTGGACGGCAACCGC<br>TTCCTGAGACTCGTCTCGCAGGGCGCCGCCCTGCTCCCCGCCG<br>ACCCCACGCGCGCCGTCGAACTGCTGGAGACCGGTCTGCGGCT<br>GTGGCGCGGGCCCGCCCTCATCGACGCGGGCGAGGGCAGGCG<br>CTGCCGGGGCGCCGCGGCCCTGTTCGAGGAACGCAGGCTCACG<br>GCCCTGGAGGATCTCATCAGCGCGATGTTCCTGCGCGGCGGCG<br>AGGCCCAGGCGATCGCCATGCTCCAGCAGCTGGTGGCGCAGTA<br>CCCACTGCGCGAGCGGTTCTGCGAGCTGCTGATGGTCGGCCTC<br>TACCGGGTGGGACGTCAGGGAGACGCCCTGGAGTCGTACCGCC<br>TGGCCAGGAAGCGGCTCGACGACGAACTGGGGGTCCAGCCGGG<br>CGCGCTGCTGCGCCGACGGCACGCCGAGATCCTCGCGCAGGAC<br>CCGGTCCTCAAGGTGCCCTCCGCGCTGTGGCGCGAGCCGTACG<br>CGCCGGCCGACACCAGCCTGCTCAGCGCCTGA

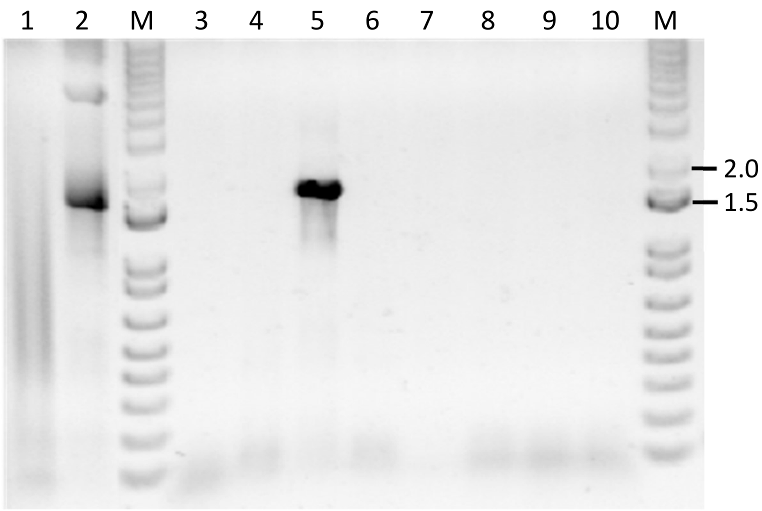

Figure S40. Representative screening of A1301 mutants integrated with *kasO**p-SCO6196 using primers ID 1 & 3 (Table S11); 1) Wild-type A1301 genome (negative control), 2) integrative plasmid (positive control), M) NEB 1 kb Plus DNA Ladder, 2.0 kb and 1.5 kb bands are annotated, 3-10) A1301 mutants. Mutants integrated with *kasO**p-SCO6196 cassette will have appearance of a band. Positive hits are further verified by sequencing.

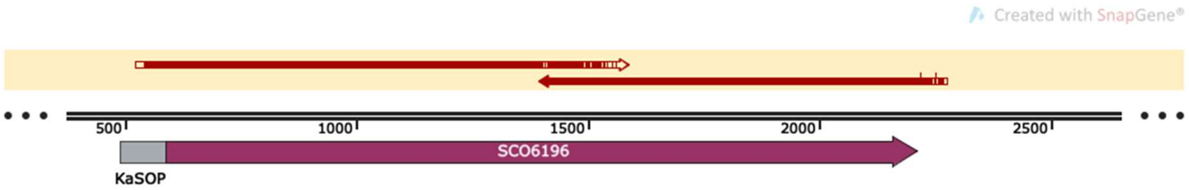

Figure S41. Representative sanger sequencing of PCR fragment to verify presence of *kasO**p and expression cassette *SCO6196* (FAS).

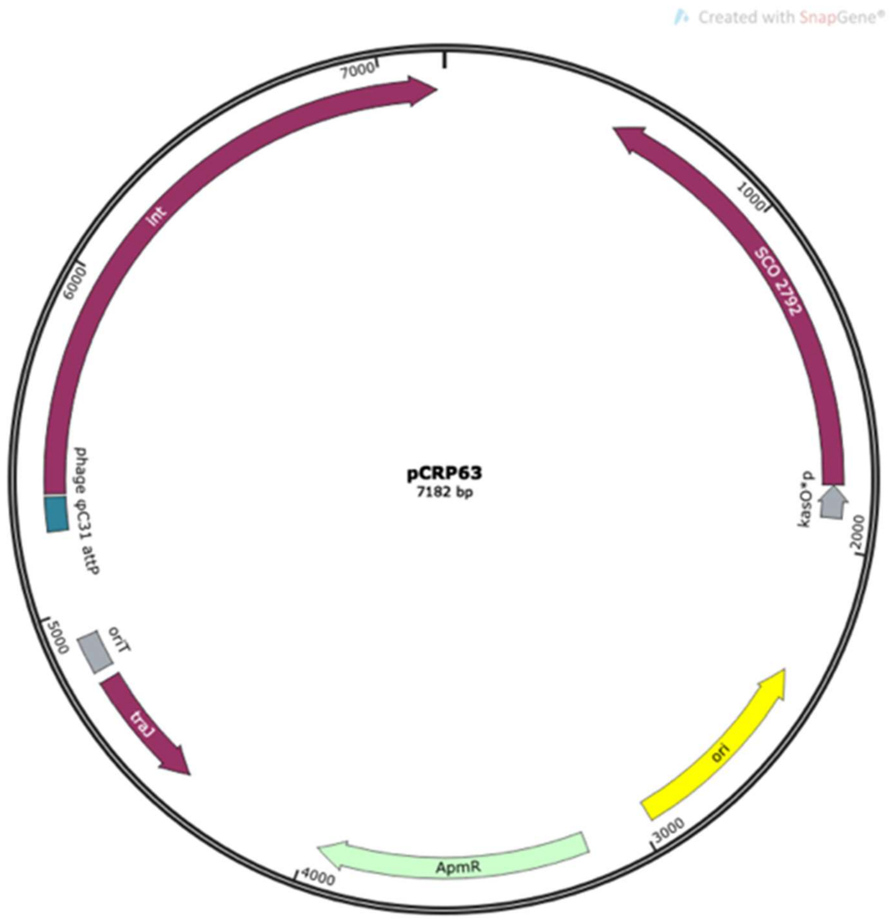

Figure S42. Plasmid map of pCRP63

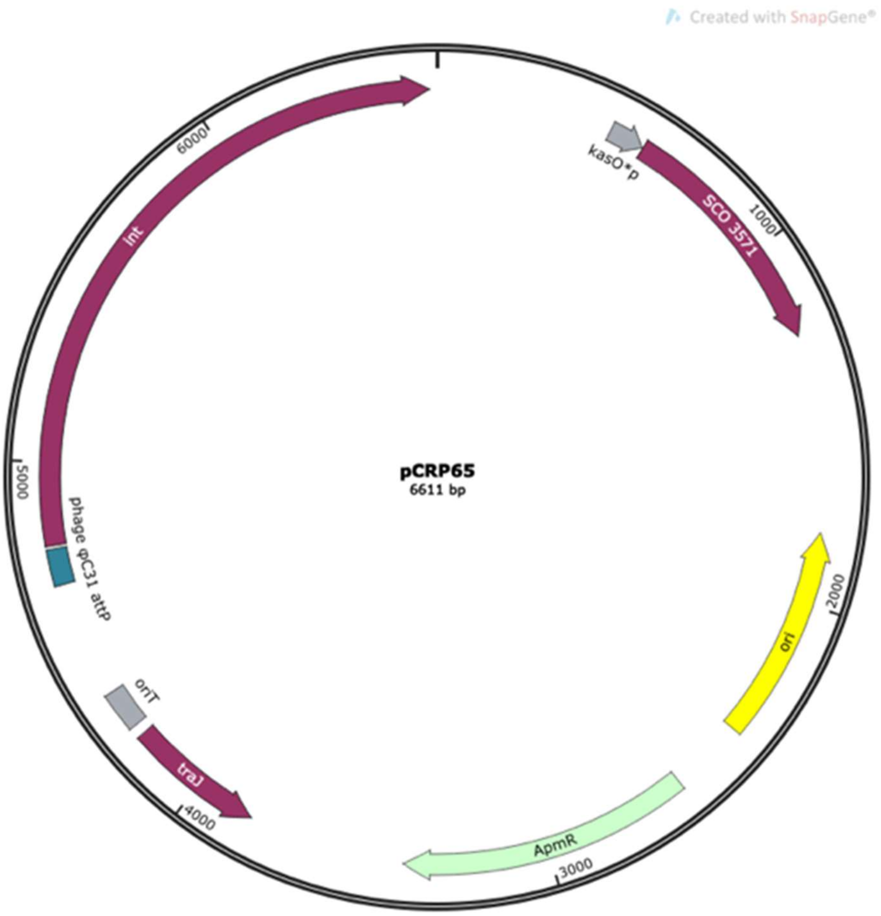

Figure S43. Plasmid map of pCRP65

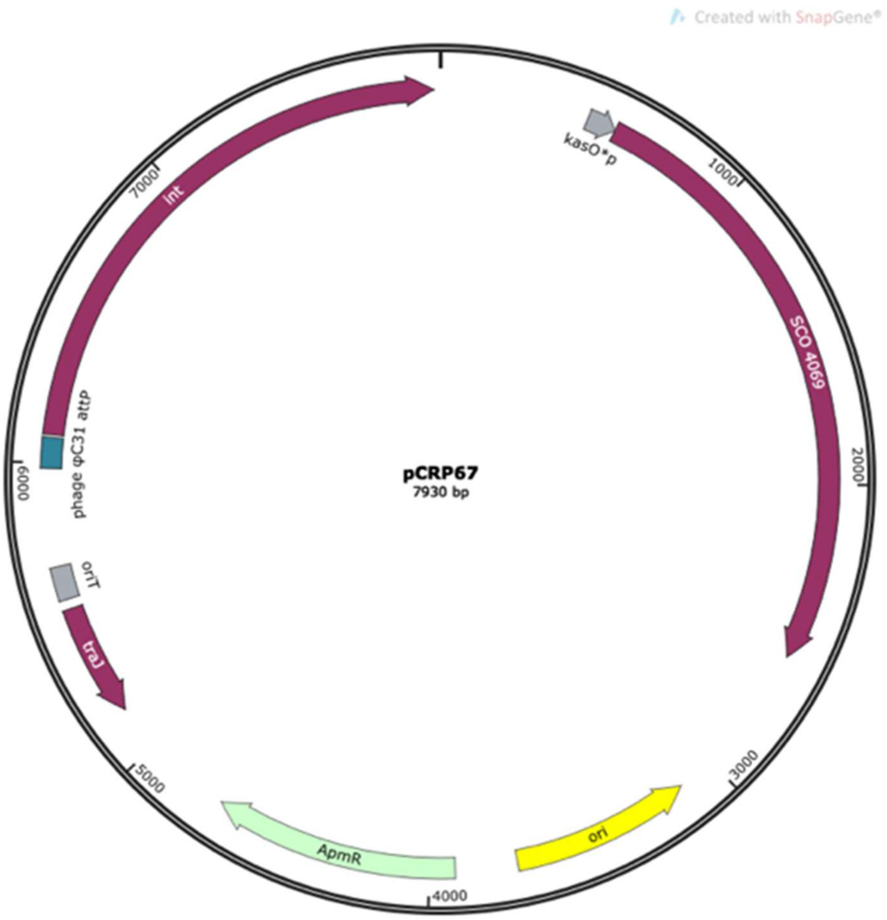

Figure S44. Plasmid map of pCRP67

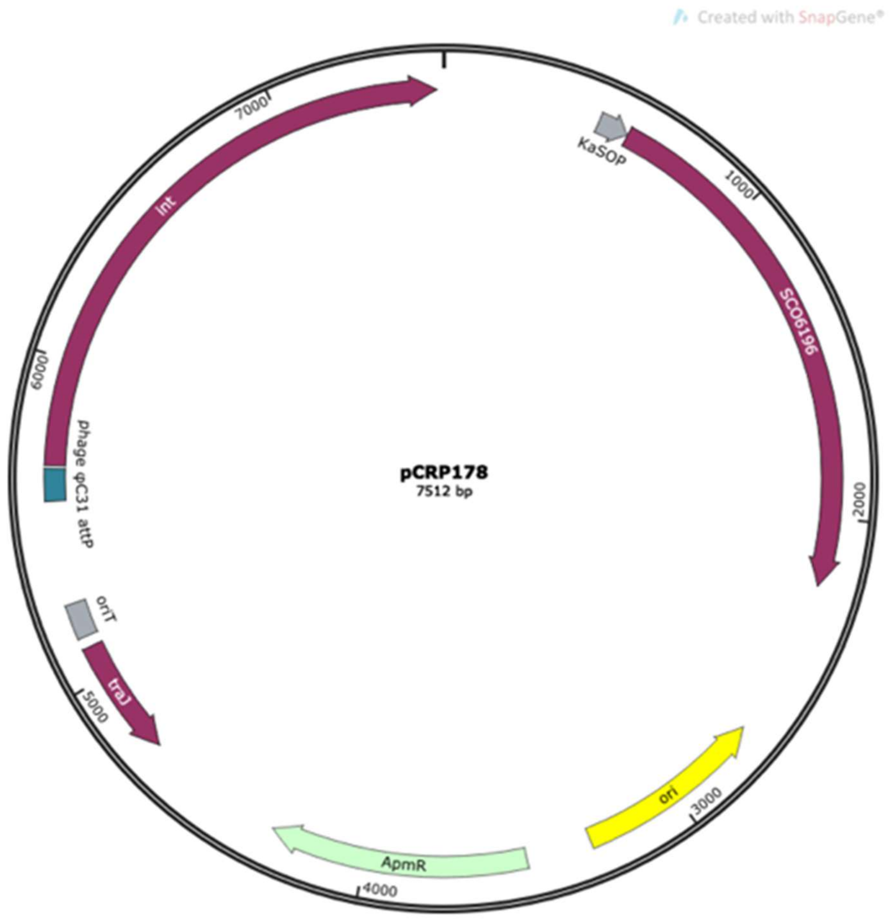

Figure S45. Plasmid map of pCRP178

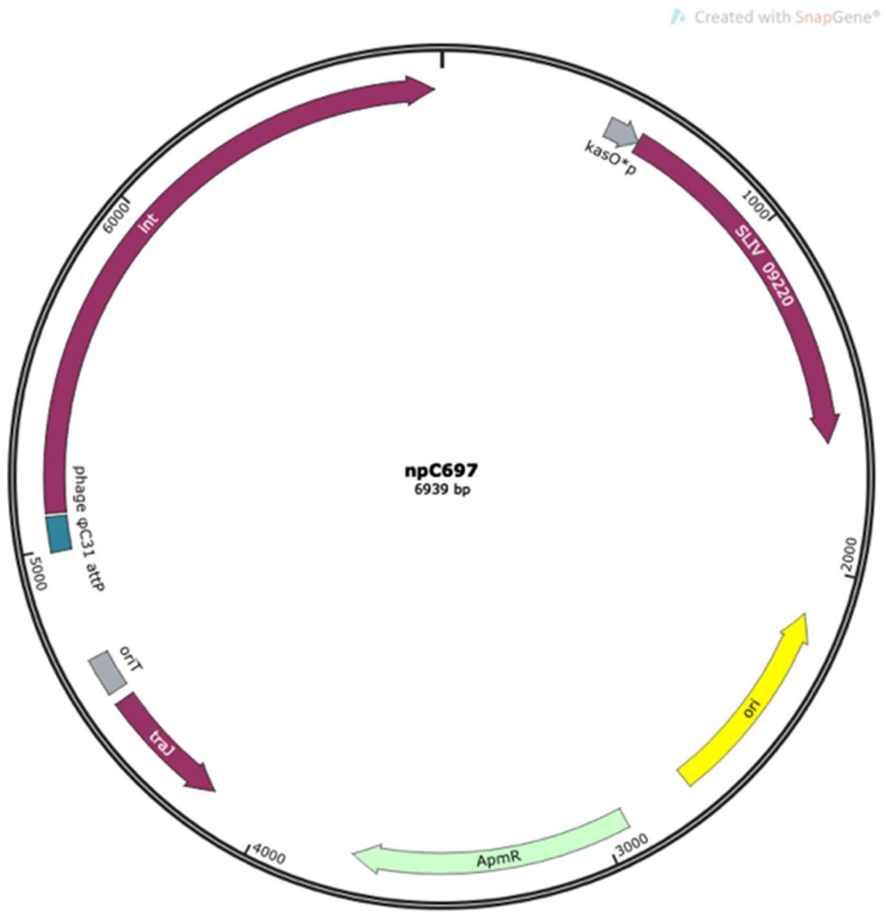

Figure S46. Plasmid map of npC697

Supplementary References

1 Whitt, J., Shipley, S. M., Newman, D. J. & Zuck, K. M. Tetramic Acid Analogues Produced by Coculture of Saccharopolyspora erythraea with Fusarium pallidoroseum. *J. Nat. Prod.* **77**, 173-177 (2014).

2 Osterhage, C., Kaminsky, R., König, G. M. & Wright, A. D. Ascosalipyrrolidinone A, an Antimicrobial Alkaloid, from the Obligate Marine Fungus Ascochyta salicorniae. *J. Org. Chem.* **65**, 6412-6417 (2000).

3 Toda, S. *et al.* A new neuritogenetic compound BU-4514N produced by Microtetraspora sp. *J. Antibiot.* **46**, 875-883 (1993).

4 Hellwig, V. *et al.* Altersetin, a new antibiotic from cultures of endophytic Alternaria spp. Taxonomy, fermentation, isolation, structure elucidation and biological activities. *J. Antibiot.* **55**, 881-892 (2002).

5 Tsukamoto, M. *et al.* Antibacterial Substance BE-54476 And Its Production. (1998).
