# Supplementary material for: Exploring a general multi-pronged activation strategy for natural product discovery in Actinomycetes
Source: Commun Biol. 2024 Jan 6;7:50. doi: 10.1038/s42003-023-05648-7 (PMC10771470; doi:10.1038/s42003-023-05648-7)
Supplement: Supplementary file 3 — Description of Additional Supplementary Data [file 42003_2023_5648_MOESM3_ESM.docx]

**Description of Additional Supplementary Files**

**File name:** Figure2_Data.xlsx

**Description:** Figure 2 Numerical Source Data

**File name:** Figure3_Data.xlsx

**Description:** Figure 3 Numerical Source Data

**File name:** Figure4_Data.xlsx

**Description:** Figure 4 Numerical Source Data

**File name:** Figure5_Data.xlsx

**Description:** Figure 5 Numerical Source Data

**File name:** Figure6_Data.xlsx

**Description:** Figure 6 Numerical Source Data

**File name:** Supplementary_Data_Tables_S1_to_S6.xlsx

**Description:** Supplementary data tables S1 to S6.
