# Supplementary material for: Exploring a general multi-pronged activation strategy for natural product discovery in Actinomycetes
Source: Commun Biol. 2024 Jan 6;7:50. doi: 10.1038/s42003-023-05648-7 (PMC10771470; doi:10.1038/s42003-023-05648-7)
Supplement: Supplementary file 9 — Reporting Summary [file 42003_2023_5648_MOESM9_ESM.pdf]

nature portfolio

COMMSBIO-23-2981-T
Corresponding author(s): Fong Tian Wong, Yee Hwee Lim

Last updated by author(s): Nov 15, 2023

Reporting Summary

Nature Portfolio wishes to improve the reproducibility of the work that we publish. This form provides structure for consistency and transparency in reporting. For further information on Nature Portfolio policies, see our Editorial Policies and the Editorial Policy Checklist.

Statistics

For all statistical analyses, confirm that the following items are present in the figure legend, table legend, main text, or Methods section.

n/a	Confirmed	
☐	☒	The exact sample size (*n*) for each experimental group/condition, given as a discrete number and unit of measurement
☐	☒	A statement on whether measurements were taken from distinct samples or whether the same sample was measured repeatedly
☐	☒	The statistical test(s) used AND whether they are one- or two-sided<br>*Only common tests should be described solely by name; describe more complex techniques in the Methods section.*
☐	☒	A description of all covariates tested
☐	☒	A description of any assumptions or corrections, such as tests of normality and adjustment for multiple comparisons
☐	☒	A full description of the statistical parameters including central tendency (e.g. means) or other basic estimates (e.g. regression coefficient) AND variation (e.g. standard deviation) or associated estimates of uncertainty (e.g. confidence intervals)
☒	☐	For null hypothesis testing, the test statistic (e.g. *F*, *t*, *r*) with confidence intervals, effect sizes, degrees of freedom and *P* value noted<br>*Give P values as exact values whenever suitable.*
☒	☐	For Bayesian analysis, information on the choice of priors and Markov chain Monte Carlo settings
☒	☐	For hierarchical and complex designs, identification of the appropriate level for tests and full reporting of outcomes
☒	☐	Estimates of effect sizes (e.g. Cohen's *d*, Pearson's *r*), indicating how they were calculated

Our web collection on statistics for biologists contains articles on many of the points above.

Software and code

Policy information about availability of computer code

Data collection	Analytical equipment control and experiment design for liquid chromatography mass spectrometry (LC-MS/MS) was carried out with Agilent MassHunter WorkStation LC/MS Data Acquisition for 6200 Series TOF / 6500 Series Q-TOF version 10.1.
Data analysis	Data manipulation, differential analysis and visualization was carried out in Microsoft Excel (Microsoft Office 365 – version 2203 – build 15028.20204). Raw liquid chromatography tandem mass spectrometry (LC-MS/MS) data was processed using MSConvert (version 3.0.22198-0867718) to the open-source mascot generic format (.mgf). Molecular networking was performed using the Global Natural Products Social Molecular Networking (GNPS) workflow on their website (https://gnps.ucsd.edu/ProteoSAFe/static/gnps-splash.jsp). Mafft (version 7.505) was used for alignment of 16S rRNA sequences. IQ-TREE (version 2.2.0.3) was used to construct phylogenetic trees. Tree visualizations were performed using iTOL webserver 9. The analysis of antimicrobial and cytotoxicity activity for their IC50 values was carried out with GraphPad Prism program (GraphPad Software, CA).

For manuscripts utilizing custom algorithms or software that are central to the research but not yet described in published literature, software must be made available to editors and reviewers. We strongly encourage code deposition in a community repository (e.g. GitHub). See the Nature Portfolio guidelines for submitting code & software for further information.

Data

Policy information about availability of data

All manuscripts must include a data availability statement. This statement should provide the following information, where applicable:

- Accession codes, unique identifiers, or web links for publicly available datasets
- A description of any restrictions on data availability
- For clinical datasets or third party data, please ensure that the statement adheres to our policy

Data that support the findings of this study are included in this paper. The full LC-MS/MS dataset has been deposited and is publicly available as a MassIVE Dataset with accession number MSV000092237.

Human research participants

Policy information about studies involving human research participants and Sex and Gender in Research.

Reporting on sex and gender	No human research was performed in this study.
Population characteristics	No human research was performed in this study.
Recruitment	No human research was performed in this study.
Ethics oversight	No human research was performed in this study.

Note that full information on the approval of the study protocol must also be provided in the manuscript.

Field-specific reporting

Please select the one below that is the best fit for your research. If you are not sure, read the appropriate sections before making your selection.

☒ Life sciences ☐ Behavioural & social sciences ☐ Ecological, evolutionary & environmental sciences

For a reference copy of the document with all sections, see nature.com/documents/nr-reporting-summary-flat.pdf

Life sciences study design

All studies must disclose on these points even when the disclosure is negative.

Sample size	Sample size were not predetermined statistically. All generated mutants and wild type strains were screened in this study.
Data exclusions	No data was excluded.
Replication	The average of two duplicated screens were used to determine primary bioactivity whilst assays were performed in triplicate for isolated compounds. Biological replicates underwent independent cell culture and fermentations, ensuring unbiased processing and reproducibility.
Randomization	Randomization is not relevant to this study.
Blinding	Blinding is not relevant to this study.

Reporting for specific materials, systems and methods

We require information from authors about some types of materials, experimental systems and methods used in many studies. Here, indicate whether each material, system or method listed is relevant to your study. If you are not sure if a list item applies to your research, read the appropriate section before selecting a response.

Materials & experimental systems

n/a	Involved in the study
☒	☐ Antibodies
☒	☐ Eukaryotic cell lines
☒	☐ Palaeontology and archaeology
☒	☐ Animals and other organisms
☒	☐ Clinical data
☒	☐ Dual use research of concern

Methods

n/a	Involved in the study
☒	☐ ChIP-seq
☒	☐ Flow cytometry
☒	☐ MRI-based neuroimaging
